# Characterization of Aminoacyl-tRNA Synthetases in Chromerids

**DOI:** 10.3390/genes10080582

**Published:** 2019-07-31

**Authors:** Abdoallah Sharaf, Ansgar Gruber, Kateřina Jiroutová, Miroslav Oborník

**Affiliations:** 1 Institute of Parasitology, Biology Centre, Czech Academy of Sciences, 370 05 České Budějovice, Czech Republic; 2 Genetics Department, Faculty of Agriculture, Ain Shams University, Cairo 11241, Egypt; 3 Faculty of Science, University of South Bohemia, 370 05 České Budějovice, Czech Republic

**Keywords:** *Chromera velia*, *Vitrella brassicaformis*, Aminoacyl tRNA synthetase (AaRS), evolution, protein localization, chloroplast, mitochondrion, nucleus

## Abstract

Aminoacyl-tRNA synthetases (AaRSs) are enzymes that catalyze the ligation of tRNAs to amino acids. There are AaRSs specific for each amino acid in the cell. Each cellular compartment in which translation takes place (the cytosol, mitochondria, and plastids in most cases), needs the full set of AaRSs; however, individual AaRSs can function in multiple compartments due to dual (or even multiple) targeting of nuclear-encoded proteins to various destinations in the cell. We searched the genomes of the chromerids, *Chromera velia* and *Vitrella brassicaformis*, for AaRS genes: 48 genes encoding AaRSs were identified in *C. velia*, while only 39 AaRS genes were found in *V. brassicaformis*. In the latter alga, ArgRS and GluRS were each encoded by a single gene occurring in a single copy; only PheRS was found in three genes, while the remaining AaRSs were encoded by two genes. In contrast, there were nine cases for which *C. velia* contained three genes of a given AaRS (45% of the AaRSs), all of them representing duplicated genes, except AsnRS and PheRS, which are more likely pseudoparalogs (acquired via horizontal or endosymbiotic gene transfer). Targeting predictions indicated that AaRSs are not (or not exclusively), in most cases, used in the cellular compartment from which their gene originates. The molecular phylogenies of the AaRSs are variable between the specific types, and similar between the two investigated chromerids. While genes with eukaryotic origin are more frequently retained, there is no clear pattern of orthologous pairs between *C. velia* and *V. brassicaformis*.

## 1. Introduction

Chromerids are single-celled photosynthetic apicomonads associated with corals [1,2,3,4,5]. Phylogenetic analyses have shown that these algae are closely related to Apicomplexa, confirming the hypothesis that apicomplexan parasites originate from a photosynthetic alga [1,6,7,8]. Only two chromerid species, *Chromera velia* and *Vitrella brassicaformis*, representing two distinct lineages, have been formally described so far [1,2]. They do not form sister species and they are considerably different in morphology, ultrastructure, pigmentation, organellar genomes, respiratory chains, and life cycles [1,2,5,8,9,10,11,12].

Chromerid genomes can be used to trace the evolutionary transition from a photosynthetic ancestor to one of the most successful groups of eukaryotic parasites [7,13,14]. Chromerid plastid genomes differ substantially in their topology, gene content, the variability of the genes they encode, and even in the genetic code, they use [4,5,10,11,15]. Chromerids possess highly reduced photosystems, which also contain several undescribed protein subunits [16]. Complete metabolic pathways, such as sugar metabolism, assimilation of nitrate and sulfite, and photosynthesis-related processes, take place in chromerid plastids [7,17]. Phylogenetic analyses [1,9,15,18] and the use of non-canonical coding for tryptophan in *C. velia* [1,15] suggest that chromerid plastids share a common ancestry with the apicomplexan relic plastid [10]. This relationship is also evident in the similar arrangement of heme biosynthesis in chromerids and apicomplexans: δ-aminolevulinic acid (ALA) is synthesized in the mitochondrion (C4 pathway) and then it is exported to the plastid [19,20]. This metabolic curiosity qualifies chromerids as the only known phototrophs that synthesize chlorophyll from glycine rather than from glutamate [19]. Chromerid mitochondrial genomes have been described as a set of heterogeneous linear molecules [8,10], like the ones found in dinoflagellates [10,21,22,23,24]. Another feature shared by chromerids and dinoflagellates is the oligoadenylation of short mito-rRNA fragments that do not seem to be spliced together [10,24]. In contrast with other myzozoans that have a conserved mitochondrial gene set consisting of three genes coding for *cox1*, *cox3*, and *cyb*, the mitochondrial genome of *C. velia* encodes only two of these proteins, with *cyb* missing. Consequently, *C. velia* has a reduced respiratory chain, lacking not only complex I like other myzozoans, but also complex III [8,10,25]. This means that the mitochondrial genome of *C. velia* has the most reduced coding capacity of those studied so far. Recently, a mitochondrion with virtually the same respiratory chain as that of *C. velia*, but lacking any genome, was discovered in the dinoflagellate *Amoebophyra* [26].

Aminoacyl-tRNA synthetases (AaRSs) ligate amino acids to specific tRNAs according to the genetic code [27]. Around 18–20 archaeal and bacterial types of AaRSs have been identified; additional AaRSs were identified in eukaryotes because they contain intracellular compartments, such as mitochondria and plastids, with their own translational activity [28,29,30,31]. The compartment-specific AaRS isoforms are often encoded by different nuclear genes [28]. Recently, several gene duplications, horizontal gene transfers, and gene losses of AaRSs have been described, as a result of the availability of the complete genome sequences [32]. Generally, each compartment in which translation takes place needs a complete set of AaRSs for the synthesis of proteins. 

Aminoacyl-tRNA synthetases (AaRSs) can be divided into two classes each with 10 enzymes, based on the conserved sequence motifs, quaternary structure, and aminoacylation function [33,34]. Class I enzymes have two consensus sequence motifs, while class II enzymes have three such motifs [33]. All enzymes show the characteristics typical for one of the above-mentioned classes, except Alanyl-tRNA synthetase (AlaRS), Phenylalanyl-tRNA synthetase (PheRS), and Glycyl-tRNA synthetase (GlyRS) (class II): in *Escherichia coli*, AlaRS contains only two of the motifs typical for class II AaRSs, while GlyRS contains only one. PheRSs possess the three typical class II motifs, but in contrast to all other class II AaRSs, they bind amino acids on the 2′-hydroxyl group (OH) of the tRNA terminal adenosine (A76) [35]. In general, AaRSs show divergent oligomeric structures: the prokaryotic GlyRSs are heterotetramers (α_2_β_2_) [35,36,37], while the eukaryotic enzymes display a homodimeric (α_2_) structure [35,38,39]. The evolutionary origins of these differences remain unclear.

There are alternative mechanisms known to be involved in the loading of tRNAs in addition to the direct loading of a specific amino acid onto its tRNA by AaRSs. Asparagine and glutamine could be misloaded onto tRNAs with the anticodons for aspartic acid and glutamic acid, respectively. When this occurs, amidotransferases can specifically convert the loaded tRNA to restore the amino acid-anticodon fit [40]. Interestingly, the absence of AaRS-encoding genes has also been reported [41,42,43,44,45]. 

Previously, AaRSs genes were automatically annotated in the genomes of *C. velia* and *V. brassicaformis* by Woo et al. [7]. Gile et al. [46] provided detailed annotations of AaRSs genes and the localization of the gene products in the diatoms *Phaeodactylum tricornutum* and *Thalassiosira pseudonana*, as well as in the cryptophyte *Guillardia theta*. Furthermore, annotations of AaRSs genes in protists are contained in the EuPathDB database [47]. The focus of this study was an exploration of the evolutionary origins of the genes encoding the chromerid Aminoacyl-tRNA synthetases. We employed a method based on Hidden Markov models (HMM) of conserved protein motifs [32] for the identification of the AaRS, and with this approach, corrected the previously published annotations for a number of genes (see results for details). The evolutionary histories of AaRSs could be used to trace the cellular evolution because of their metabolic universality and their essential role in protein synthesis [48]. AaRSs commonly have irregular evolutionary patterns due to gene duplications, high levels of sequence divergence, and horizontal gene transfers [49], but some AaRS trees show clear ancestral relationships [48]. Since in chromerids, no Aminoacyl-tRNA synthetases are plastid or mitochondrially encoded, they are all encoded by nuclear genes, and translated in the cytosol, with posttranslational targeting to the translationally active organelles.

## 2. Material and Methods

### 2.1. Gene Identification and Model Assessment

The genomes of the chromerids (*C. velia* and *V. brassicaformis*) and a closely related apicomplexan (*Toxoplasma gondii*) were searched for AaRSs with the BLASTp tool in the Eukaryotic Pathogen database (https://eupathdb.org) [7,47], using previously characterized AaRS amino acid sequences as queries [32]. We validated the selected gene models by checking the transcription of the 5’-end of the gene model by BLAST against the identified genes from *C. velia* and *V. brassicaformis* transcriptomes [7]. Conserved protein motifs in each of the AaRS loci were identified by the Hidden Markov model (HMM)-based tool for the AaRS coding sequence detection with a cut-off of 5 out of 10 motifs [32,50]. Applying the former analysis pipeline on the Swiss–Prot annotated AaRSs showed that this pipeline could capture and annotate 99.8% of the AaRSs [32].

### 2.2. Localization Prediction

We predicted the protein N-termini (starting with a methionine residue) preceding the conserved AaRS domain; also tested the gene model for possible gene fusion using the genome browser (JBrowse) in the CryptoDB database (https://cryptodb.org/cryptodb/) [47]. Targeting predictions of endoplasmic reticulum (ER) signal peptides were performed using SignalP 3.0 [51] and SignalP 4.1 [52], the bipartite targeting sequences (BTS) were searched by ASAFind [53], transit peptides by TargetP 1.1 [54], and mitochondrial transit peptides were predicted using iPSORT [55], WoLF PSORT [56], and Mitoprot [57] (Table 1 and Supplementary Table S1A). Dual targeting by an ambiguous targeting presequence was assumed for those AaRSs predicted to possess localization signals for several different subcellular compartments. 

### 2.3. Molecular Phylogeny

Amino acid sequences of all 20 Aminoacyl-tRNA synthetases from plants, chlorophytes, rhodophytes, alveolates (apicomplexans and ciliates), stramenopiles (oomycetes and diatoms), metazoans, fungi, kinetoplastids, amoebozoans, parabasalids, and diplomonads were downloaded from the NCBI GeneBank (www.ncbi.nlm.nih.gov) and specialized databases (www.genome.jgi-psf.org; www.paramecium.cgm.cnrs-gif.fr; www.merolae.biol.s.u-tokyo.ac.jp; www.ensembl.org; www.arabidopsis.org). The available bacterial homologs were obtained from the prokaryotic AaRS database (www.bioinf.bio.uth.gr/AaRS/#/), with special emphasis on those prokaryotes that are supposed to be involved in endosymbiotic events, such as cyanobacteria (the supposed ancestor of primary plastids) and α-proteobacteria (the supposed ancestor of mitochondria). The sequences were aligned using the MAFFT software [58]; ambiguously aligned sequences and gaps were excluded from further analysis. The alignments were tested using Prottest [59,60] to specify the appropriate amino acid substitution model. The maximum likelihood method, as implemented in PhyML (www.atgc-montpellier.fr/phyml) [61,62], and Bayesian inference (Mr. Bayes; www.mrbayes.sourceforge.net) [63], were used to construct the phylogenetic trees. 

## 3. Results and Discussion 

### Gene Identification and in Silico Localization of Gene Products

BLAST search and motif screening of the total predicted proteomes of the two chromerids (*C. velia* and *V. brassicaformis*) and their close relative, the apicomplexan *T. gondii*, resulted in the identification of 48, 39, and 31 AaRS genes, respectively, with some of the previous annotations corrected (Table 1 and Supplementary Table S1A). Certainly, this is substantially less than the expected number (roughly 60) necessary for providing AaRSs to all three translationally active compartments—the cytosol, plastid, and mitochondrion—via exclusive transport mechanisms that provide only one gene product to only one intracellular location. AaRSs are generally conserved; therefore, it is unlikely that any additional enzymes were missed during our search due to sequence diversity. In the diatoms *Thalassiosira pseudonana* and *Phaeodactylum tricornutum*, and in the cryptophyte *Guillardia theta*, our genome search and motif screening of previously identified AaRSs revealed 17.5% misidentified genes in the diatom *Thalassiosira pseudonana,* 5% in the diatom *Phaeodactylum tricornutum*, and 7% in the cryptophyte *Guillardia theta* compared to Gile et al. [46] (Table 1 and Supplementary Table S1A). For example, the genes Jgi_51430 and Jgi_42226 were identified as cytoplasmic Glu-tRNA synthetases (GluRSs) in *P. tricornutum* and *T. pseudonana* by Gile et al. [46]; however, our motif search revealed that these genes contain three GluRS specific motifs in *T. pseudonana* and only two such motifs in *P. tricornutum*, while they possess six (*T. pseudonana*) and five (*P. tricornutum*) GlnRS specific motifs instead (Supplementary Table S1A). Other examples include the plastid-localized Jgi_24163 and periplastidal Jgi_68323 proteins, identified as the α-subunit of PheRS (α-PheRS) in *T. pseudonana* and *G. theta* [46]. Again, our motif search revealed that they contain four and three motifs for *T. pseudonana* and *G. theta*, respectively, which is below the standard cut-off value [32], meaning the genes probably encode other proteins than AaRSs (Supplementary Table S1A).

**Figure 1:**
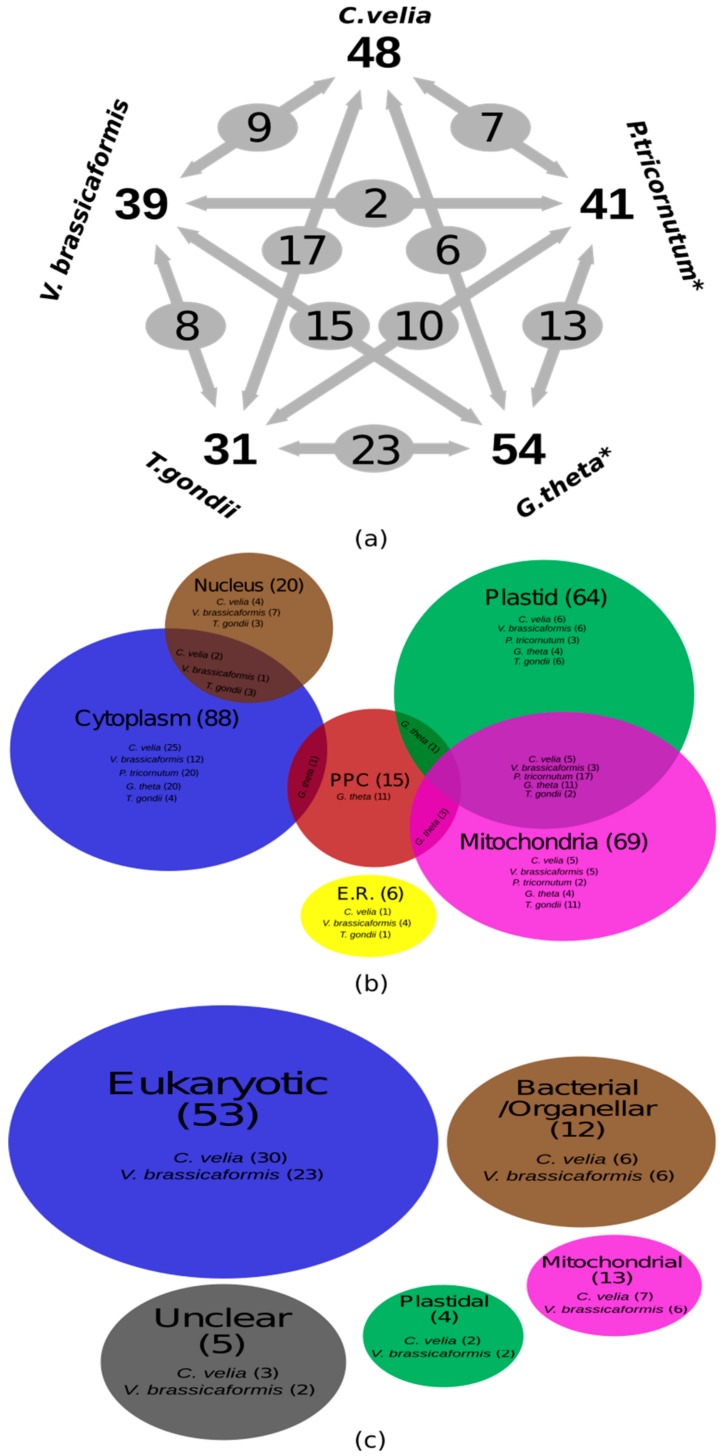
(**a**) Numbers of Aminoacyl-tRNA synthetases in the chromerids (*Chromera velia* and *Vitrella brassicaformis*), in comparison to a diatom (*Phaeodactylum tricornutum*), a cryptophyte (*Guillardia theta*), and the apicomplexan *Toxoplasma gondii*; (**b**) Euler diagram representing the protein subcellular localization results of Aminoacyl-tRNA synthetases (AaRS) in the chromerids, *P. tricornutum*, *G. theta* and *T. gondii*; (**c**) Euler diagram representing the evolutionary origins of the chromerid AaRSs.

The chromerids each have a different number of genes coding for AaRSs. Nine (45%) AaRSs in *C. velia* are encoded by three distinct genes; whereas, ten (45%) are encoded by two distinct genes. Interestingly, only GluRS was encoded by a single gene (Figure 1a and Table 1). Our premise assumes that each compartment with translational activity needs an AaRS for each amino acid. Since all AaRSs in chromerids are nuclear-encoded [7,8,15], and only nine AaRSs are present in three copies in the genome, as well as five that are targeted to all three compartments, the remaining AaRSs must be dually or even multiply targeted to the cytosol, the mitochondrion, and the plastid to satisfy the need for a complete set of AaRS for each of the three subcellular compartments in which translation takes place. 

Alternatively, the activity of some AaRSs could be replaced by a tRNA-dependent amino acid transformation mechanism [40]. In this study, we found that both *C. velia* and *V. brassicaformis* possess two genes coding for Glutamyl-tRNA^Gln^ amidotransferase (Glu-AdT). Both *Chromera*’s (cvel_23134) and *Vitrella*’s (vbra_8651) Glu-AdT were predicted to be targeted to the plastid (Supplementary Table S1B), in which Glu-tRNA^Gln^ is most likely transamidated to produce Gln-tRNA^Gln^. Moreover, two plastid-targeted Aspartyl/Glutamyl-tRNA^Asn/Gln^ amidotransferases (Asp/Glu-AdT) were identified in *C. velia* (cvel_28674 and cvel_12310), and one plastid-targeted Asp/Glu-AdT was identified in *V. brassicaformis* (vbra_10654). This could compensate for the lack of Asparaginyl-tRNA (AsnRS) and Glutaminyl-tRNA (GlnRS) synthetases in the plastids of both investigated chromerids (Supplementary Table S1B), producing Asn-tRNA^Asn^ or Gln-tRNA^Gln^ through the transamidation of misacylated Asp-tRNA^Asn^ or Glu-tRNA^Gln^, respectively.

We identified many AaRSs putatively localized to the nucleus. Since tRNAs are formed in the nucleus, these enzymes could be involved in the transport of tRNAs to the cytoplasm. The localization of AaRSs to the nucleus has been shown in numerous previous studies on nuclear aminoacylation of tRNAs [64,65,66]. Recently, at least 13 active AaRSs were found in purified mammalian cell nuclei [66,67]. In general, nucleus-targeted AaRSs are known to play a role in tRNA maturation and tRNA export control. The human nuclear enzyme, MetRS seems to be related to the biogenesis of rRNA in nucleoli, in addition to its catalytic activity in protein synthesis in the cytoplasm [66,68].

A quarter of the AaRSs (LeuRS, MetRS, TrpRS, PheRS and ThrRS) in *C. velia* were predicted to be targeted to all three compartments (Figure 1a and Table 1). In *C. velia*, GlyRS and ProRS lack any targeting presequence, which suggests they are only localized to the cytoplasm; the same has been experimentally proven for human GlnRS [69] (Table 1 and Supplementary Table S1A). Ten AaRSs (GluRS, AspRS, CysRS, GlyRS, HisRS, IleRS, ProRS, SerRS, TyrRS, and ValRS) do not possess a mitochondrial transit peptide in *C. velia* (Figure 1a). This is a common scenario known from ArgRS and LeuRS in *Arabidopsis* [70,71], GluRS and GlnRS in *T. pseudonana,* and GlnRS in both *P. tricornutum* and trypanosomatids [46,72,73] (Supplementary Table S1). We did not detect the BTS necessary for plastid targeting in 45% of the AaRSs in *C. velia*; it was missing from AlaRS, ArgRS, AsnRS, LysRS, GluRS, ValRS, GlyRS, ProRS, and SerRS (Figure 1a and Table 1). The same has been observed in *Arabidopsis*, with GluRS, GlyRS, TrpRS, and TyrRS lacking chloroplast transit peptides [70] (Supplementary Table S1A). BTS presequences are also absent in AlaRS, AspRS, IleRS, and ValRS in *Guillardia theta* (Figure 1a). On the other hand, almost complete sets of plastid-targeted AaRSs were found in the diatoms, *T. pseudonana* and *P. tricornutum* [46], except PheRS in *T. pseudonana* (Figure 1a and Supplementary Table S1A). Cytoplasmic AlaRS and GluRS were absent from both chromerids, only plastid GluRS was identified. Finally, SerRS was predicted to be targeted to the cytoplasm, nucleus, and endoplasmic reticulum (Endo.) (Figure 1a and Supplementary Table S1).

In *V. brassicaformis*, 17 (85%) of the AaRSs were encoded by two genes; whereas, ArgRS and GluRS were only encoded by a single gene (Figure 1a and Table 1). PheRS was encoded by three genes, two codings for the α-subunit and one for the β-subunit. Dual targeting by an ambiguous targeting sequence was identified in AsnRS, GlyRS, LeuRS, and α-PheRS. Like in *C. velia*, nucleus-localized AlaRS, ArgRS, LeuRS, LysRS, α-PheRS, SerRS, and ThrRS were also found in *V. brassicaformis*. Only GlyRS was predicted to be targeted to all three compartments, employing a dual-targeting mechanism in one of the two predicted genes (vbra_14962) (Figure 1a and Table 1). In contrast to *C. velia’s* GlnRS, GlyRS, ProRS, and TrpRS, none of the *V. brassicaformis* AaRSs were targeted only to the cytoplasm (Figure 1a and Table 1). Interestingly, in *V. brassicaformis*, AlaRS, AsnRS, AspRS, CysRS, GlnRS, GlyRS, LeuRS, and TrpRS were identified as mitochondrial-targeted proteins, while AlaRS, ArgRS, CysRS, GlnRS, IleRS, LysRS, PheRS, ProRS, SerRS, TrpRS, and TyrRS do not seem to have plastid-targeting signals (Figure 1a and Table 1). Furthermore, AlaRS, ArgRS, AsnRS, AspRS, GluRS, LeuRS, ThrRS, and TrpRS were identified to be localized in the organelles. Finally, IleRS, ProRS, TrpRS, and TyrRS were predicted to be localized to the endoplasmic reticulum (Figure 1a and Table 1). 

GlyRSs are a special class of Aminoacyl-tRNA synthetases because they have variable functional properties and divergent oligomeric structures [35,74]. Prokaryotic GlyRSs recognize tRNA molecules with a U73 discriminator base; whereas, the eukaryotic ones recognize them with A73 [35,39,74,75]. This suggests that the interaction between the structure and function differs between prokaryotic and eukaryotic glycation systems. The oligomeric structure of most AaRSs is conserved, and sequence comparisons reveal significant similarities. In contrast, GlyRSs show high structural divergence, which leads to the non-conservation of the oligomeric structure [35]. The subunit structure of GlyRSs is not conserved in prokaryotes: two oligomeric types of GlyRS are found in nature (see above). The α_2_ type has been identified in all three kingdoms of life, while the α_2_β_2_ type is only found in bacteria and chloroplasts [35].

Only two α_2_-type enzymes were identified in *C. velia*, both providing cytoplasmic functions. Conversely, *V. brassicaformis* contained enzymes of the α_2_-type (vbra_22902) and the fused (αβ)_2_-type (vbra_14962), functioning in all three compartments (Figure 1a and Table 1). The fused (αβ)_2_-type enzyme was previously found in *Arabidopsis thaliana* and functions in both mitochondria and chloroplasts [76]. GlyRSs α2-type (vbra_22902) and the fused (αβ)2-type (vbra_14962) in *V. brassicaformis* show low amino acid sequences (17.3 %), but both genes contain 10 GlyRS functional motifs, which support the importance of motif scanning during the gene identification pipeline [32]. 

## 4. Phylogenetic Analyses

**Figure 2:**
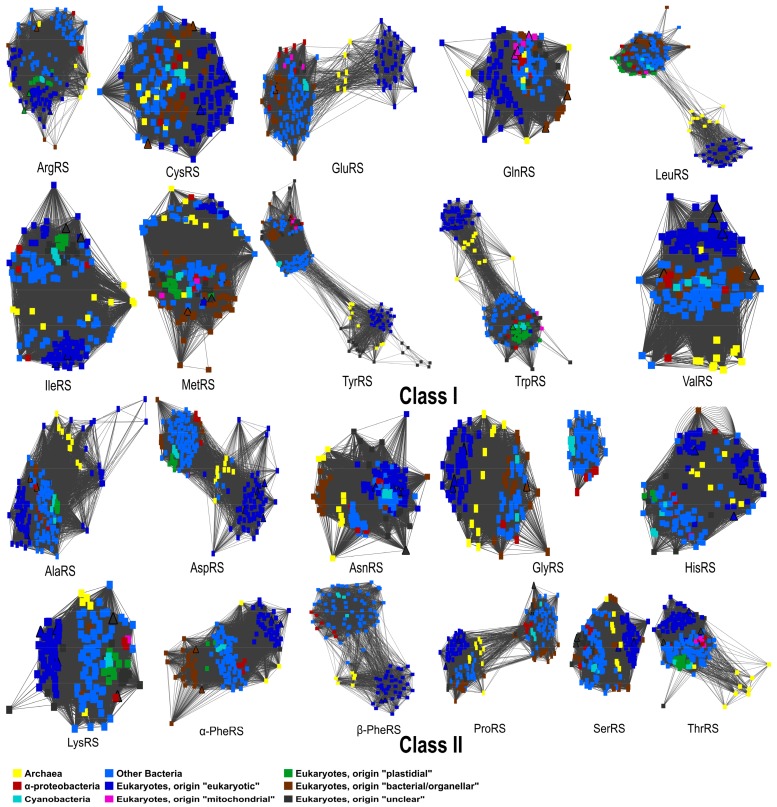
A similarity network for each sequence dataset of the 20 different Aminoacyl-tRNA synthetases shows the sequence similarity and reflects the phylogenetic tree topology of each Aminoacyl-tRNA enzyme.

We performed phylogenetic analyses of all 20 AaRSs from chromerid algae. Similarity networks for the whole set of Aminoacyl-tRNA synthetases reflect the tree topology of each Aminoacyl-tRNA synthetase [77] (Figure 2). Out of the 21 computed trees (PheRS has two subunits), only the β-PheRS tree fully corresponds to the classical three-domain pattern that is postulated for eukaryotic enzymes. The archaeal and eukaryotic AaRSs were well separated from the bacterial group, in agreement with previous studies [48]. The remaining trees show two or more divergent bacterial clades (Figure 2). Furthermore, in both chromerids, endosymbiotic gene transfer events were only observed in ArgRS and TrpRS. Interestingly, for LeuRS and TyrRS there are two unrelated groups, likely the result of independent evolution of the eukaryotic and prokaryotic versions of these two enzymes (Figure 2). The remarkable dissimilarities of various regions in bacterial and eukaryotic LeuRS and TyrRS have also been observed in previous studies [48,78]. 

AaRSs represents an example of the true ancestral paralogous evolved from gene duplication, but it is also a Horizontal Gene Transfer (HGT) leading to pseudoparalogy [79]. Out of nine cases for which *C. velia* contained three genes of a given AaRS (Table 1), we identify gene duplication in seven *C. velia*’s AaRSs (GlnRS, ValRS, AlaRS, AspRS, GlyRS, LysRS, and SerRS) while AsnRS and PheRS are more likely pseudoparalogs (Supplementary Figure S1 and File sf1). In contrast, no gene duplication was identified in *V. brassicaformis*’s AaRSs.

Bacterial PheRS consists of an α and β subunit encoded by the pheS and pheT genes, canonically located on the same operon [80]. Mitochondrial PheRS is a fusion of the N-terminal part of the α-subunit and the C-terminal part of the β-subunit [81]. We concatenated the two subunits into one sequence. Nevertheless, we constructed phylogenetic trees for the α- and β-subunits separately, to ensure that the PheRS tree topology is not affected by mixed signals from the two PheRS subunits. Analyzing the subunits individually results in the same topology as for the concatenated sequences (Supplementary Figure S1). We also constructed a phylogenetic tree from the combined sequences, representing both GlyRSs, types α_2_ and α_2_β_2_. The similarity network and the phylogenetic tree confirmed that the enzymes of type α_2_β_2_ are only present in bacteria, resulting in a disconnected network and the formation of a clade that is separate from enzyme type α_2_ (Figure 2, Supplementary Figure S1 and File sf1). All three domains of life have the α_2_-type enzyme, including both chromerids (Figure 2, Supplementary Figure S1 and File sf1).

In general, *C. velia* and *V. brassicaformis* AaRSs show similar evolutionary patterns (Figure 1b and Table 1), with the exception of AgrRS, AsnRS, and GlyRS. Seven (14.6%) of the identified genes in *C. velia* and six (15.4%) genes in *V. brassicaformis* are mitochondrial in origin (Figure 1b and Table 1). In *C. velia* and *V. brassicaformis respectively*, 30 out of the 48 (62.5%) and 23 out of the 39 (59%) identified AaRSs originate from the eukaryotic nucleus. Six AaRSs show bacterial/organellar origin in both chromerids (Table 1). We were unable to specify the origin of three (6.2%) and two (5.1%) of the AaRSs from *C. velia* and *V. brassicaformis*, respectively (Table 1 and Supplementary File sf1). Since chromerids appear to be closely related to apicomplexan parasites, we also looked at the origins of AaRSs in the apicomplexan *T. gondii*. In this parasite, 21 (67.7%) of the identified AaRS genes originate from the eukaryotic nucleus, while 3 and 4 AaRSs display bacterial/organellar and mitochondrial origin, respectively. We were unable to specify the origin of two (6.4%) AaRS genes from *T. gondii* (Supplementary Figure S1 and File sf1). This apicomplexan and the chromerids have homologous evolutionary patterns in all of their AaRSs except GluRS, supporting the close evolutionary relationship between apicomplexans and chromerids (Supplementary Figure S1 and File sf1).

## 5. Conclusions

This study identified sequences of Aminoacyl-tRNA synthetases (AaRSs) in chromerids, along with their putative protein localization and evolutionary origins. We identified 48 and 39 AaRS genes in *C. velia* and *V. brassicaformis*, respectively, representing the full set of 20 AaRS types. Five AaRSs were predicted to be targeted to all three compartments (mitochondrion, plastid, and cytosol) by an ambiguous targeting sequence that likely leads to the dual-targeting of these enzymes in *C. velia*; whereas, only two such AaRSs were predicted in *V. brassicaformis.* We identified five (*C. velia*) and eight (*V. brassicaformis*) nucleus-localized AaRSs in the chromerids, which we assume to be first targeted to the nucleus and then to the cytoplasm. GlnRS and AsnRS were absent from the plastids of both chromerids; their activity is likely restored by tRNA-dependent amino acid transformation mechanisms.

The α_2_-type of GlyRS was identified in *C. velia*; whereas, both the α_2_-type and the fused (αβ)_2_-type of GlyRS were found in *V. brassicaformis*. We identified gene duplications of seven AaRSs (GlnRS, ValRS, AlaRS, AspRS, GlyRS, LysRS, and SerRS) in *C. velia*, while no AaRS gene duplication was found in *V. brassicaformis*. Both LeuRS and TyrRS have two disconnected eukaryotic and prokaryotic groups, which could be a result of the independent evolution of the two versions of these enzymes. In both chromerids, ArgRS and TrpRS were shown to be acquired from endosymbionts by endosymbiotic gene transfer; all other genes are of eukaryotic origin with proteins targeted to the various compartments. The tree topologies suggest that numerous gradual losses of pseudoparalogs occurred in eight enzymes (ArgRS, CysRS, IleRS, MetRS, AlaRS, HisRS, LysRS, and ThrRS). 

Given that *C. velia* and *V. brassicaformis* are such closely related organisms, the number of differences in their AaRS genes is much higher than we would expect. The intracellular targeting of AaRSs is independent of their evolutionary origin. There is no clear pattern of orthologous pairs that are retained in both organisms. Instead, gene duplications, gene losses, and changes in targeting signals account for the required activities of tRNA synthesis in the different cellular compartments.

## Figures and Tables

**Table 1:** Summary of the subcellular localization and origin of the identified Aminoacyl-tRNA synthetases (AaRS) in chromerids (*Chromera velia* and *Vitrella brassicaformis*). Four letters are used to describe the predicted localization of AaRSs (Cyto. = Cytoplasm; Nucl. = Nucleus; Mito. = Mitochondria; Plas. = Plastid; Endo. =E ndoplasmic reticulum) and their origins (Euka. = Eukaryotic; Mito. = Mitochondrial; Plas. = Plastidal; Bact./Orga. = Bacterial/Organellar; Uncl. = Unclear), while the number of transcripts is between parenthesis if it was found.

AaRSs	*Chromera velia*			*Vitrella brassicaformis*		
	48			39		
	Gene	Localization	Origin	Gene	Localization	Origin
**ArgRS**	cvel_7363	Cyto.|Nucl.	Bact./Orga.	vbra_19912	Nucl.	Plas.
	cvel_22194	Cyto.	Euka.			
	cvel_3008	Mito.	Plas.			
**CysRS**	cvel_18990	Cyto	Euka.	vbra_21787	Cyto.	Euka.
	cvel_1412	Plas.	Bact./Orga.	vbra_7006	Mito.	Bact./Orga.
**GluRS**	cvel_31597	Plas.	Bact./Orga.	vbra_21912	Plas.	Bact./Orga.
**GlnRS**	cvel_8110	Cyto.	Mito.	vbra_11768	Cyto.	Mito.
	cvel_1112	Cyto.	Mito.	vbra_19782	Mito.	Bact./Orga.
	cvel_874	Mito.	Bact./Orga.			
**LeuRS**	cvel_7809	Plas.|Mito.	Bact./Orga.	vbra_9619	Plas.|Mito.	Bact./Orga.
	cvel_16973	Cyto.	Euka.	vbra_9824	Nucl.	Euka.
**IleRS**	cvel_28423	Plas.	Euka.	vbra_7770	Cyto.	Euka.
	cvel_28875	Cyto.	Euka.	vbra_11931	Endo.	Euka.
**MetRS**	cvel_21221	Cyto.	Euka.	vbra_1113	Plas.	Mito.
	cvel_7165	Plas.|Mito.	Mito.	vbra_13101	Cyto.	Euka.
**TrpRS**	cvel_7641	Cyto.	Euka.	vbra_16713	Mito.	Euka.
	cvel_20758	Plas.|Mito.	Plas.	vbra_20646	Endo.	Plas.
**TyrRS**	cvel_7146	Plas.	Mito.	vbra_8727	Endo.	Mito.
	cvel_4070	Cyto.	Euka.	vbra_13490	Cyto.	Euka.
**ValRS**	cvel_6889	Nucl.	Euka.	vbra_14431	Plas.	Euka.
	cvel_16754	Cyto.	Euka.	vbra_19209	Cyto.	Euka.
	cvel_9091	Cyto.	Euka.			
**AlaRS**	cvel_11640	Nucl.	Bact./Orga.	vbra_6631	Nucl.	Euka.
	cvel_19371	Mito.	Euka.	vbra_13386	Mito.	Bact./Orga.
	cvel_21018	Nucl.	Euka.			
**AspRS**	cvel_29834	Cyto.	Euka.	vbra_13004	Mito.	Euka.
	cvel_4486	Cyto.	Euka.	vbra_6695	Plas.	Euka.
	cvel_13271	Plas.	Euka.			
**AsnRS**	cvel_14360	Cyto.	Uncl.	vbra_5336	Plas.|Mito.	Euka.
	cvel_7405	Mito.	Euka.	vbra_5330	Mito.	Euka.
	cvel_23534	Cyto.	Euka.			
**GlyRS**	cvel_30153	Cyto.	Euka.	vbra_22902	Cyto.	Euka.
	cvel_8554	Cyto.	Euka.	vbra_14962	Plas.|Mito.	Bact./Orga.
**HisRS**	cvel_21705	Cyto.	Euka.	vbra_20528	Plas.	Euka.
	cvel_3311(2)	(2) Plas.	Euka.	vbra_6931	Cyto.	Euka.
**LysRS**	cvel_9397	Mito.	Mito.	vbra_6201	Cyto.	Euka.
	cvel_20258	Cyto.	Euka.	vbra_4678	Nucl.	Mito.
	cvel_7175	Cyto.|Nul.	Euka.			
**α-PheRS**	cvel_9563	Plas.|Mito.	Uncl.	vbra_15842	Cyto.|Nucl.	Uncl.
	cvel_5984 (3)	(3) Cyto.	Euka.	vbra_3529	Nucl.	Euka.
**β-PheRS**	cvel_22325	Cyto.	Euka.	vbra_20531	Cyto.	Euka.
**ProRS**	cvel_26651	Cyto.	Uncl.	vbra_17540	Cyto.	Euka.
	cvel_17161	Cyto.	Euka.	vbra_8238	Endo.	Uncl.
**SerRS**	cvel_28365	Cyto.	Euka.	vbra_2397	Cyto.	Euka.
	cvel_4888	Nucl.	Euka.	vbra_7841	Nucl.	Mito.
	cvel_18368	Endo.	Mito.			
**ThrRS**	cvel_8549	Plas.|Mito.	Mito.	vbra_13128	Nucl.	Euka.
	cvel_11445	Cyto.	Euka.	vbra_14881	Plas.	Mito.

## Supplementary Materials

The following supplementary materials are available online at https://www.mdpi.com/2073-4425/10/8/582/s1. Table S1: (A) Summary of the protein subcellular localization of the identified Aminoacyl-tRNA synthetases (AaRSs) in the chromerids (*Chromera velia* and *Vitrella brassicaformis*), diatoms (*Phaeodactylum tricornutum* and *Thalassiosira pseudonana*), cryptophyte (*Guillardia theta*), apicomplexan (*Toxoplasma gondii*), archeplastida (*Arabidopsis thaliana*), and opisthokont (*Homo sapiens*), previous gene identification for (*) chromerids by Woo et al., 2015 [7], (**) diatoms and the cryptophyte *Guillardia theta* by Gile et al., 2015 [46] and (***) *Toxoplasma gondii* in EuPathDB [47] were inserted. (B) Summary of the subcellular protein localization of the identified amidotransferase genes in the chromerids, Figure S1: Phylogenetic supertrees of all identified Aminoacyl-tRNA synthetases (AaRSs) from chromerids and other domains of life, based on protein sequences, branch support values are given in % (maximum likelihood/Bayesian inference), Supplementary File sf1: Interpretation of all identified Aminoacyl-tRNA synthetases (AaRS) phylogenetic supertrees.
