# Supplementary material for: Characterization of Aminoacyl-tRNA Synthetases in Chromerids
Source: Genes (Basel). 2019 Jul 31;10(8):582. doi: 10.3390/genes10080582 (PMC6723311; doi:10.3390/genes10080582)
Supplement: Supplementary file 1 [file genes-10-00582-s001.zip › Supplementary Materials/Figure S1.pdf]

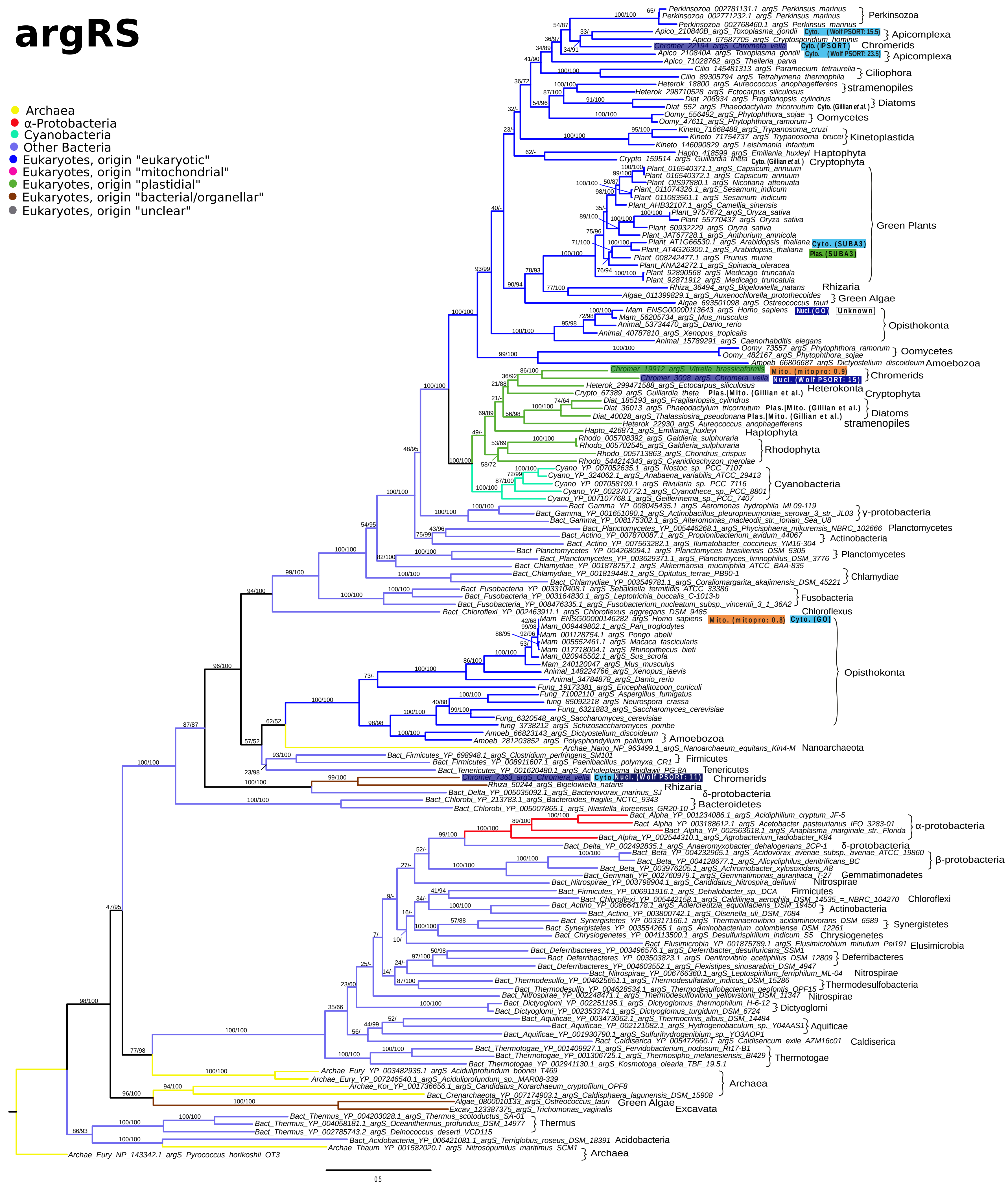
argRS
Archaea
α-Protobacteria
Cyanobacteria
Other Bacteria
Eukaryotes, origin "eukaryotic"
Eukaryotes, origin "mitochondrial"
Eukaryotes, origin "plastidial"
Eukaryotes, origin "bacterial/organellar"
Eukaryotes, origin "unclear"
Perkinsozoa_002781131.1_argS_Perkinsus_marinus
Perkinsozoa_002771232.1_argS_Perkinsus_marinus
Perkinsozoa_002768460.1_argS_Perkinsus_marinus
Perkinsozoa
Apico_210840B_argS_Toxoplasma_gondii
Cyto. (Wolf PSORT: 15.5)
Apico_67587705_argS_Cryptosporidium_hominis
Apicomplexa
Chromer_22194_argS_Chromera_velia
Cyto. (iPSORT)
Chromerids
Apico_210840A_argS_Toxoplasma_gondii
Cyto. (Wolf PSORT: 23.5)
Apico_71028762_argS_Theileria_parva
Apicomplexa
Cilio_145481313_argS_Paramecium_tetraurelia
Cilio_89305794_argS_Tetrahymena_thermophila
Ciliophora
Heterok_18800_argS_Aureococcus_anophagefferens
Heterok_298710528_argS_Ectocarpus_siliculosus
stramenopiles
Diat_206934_argS_Fragilariopsis_cylindrus
Diat_552_argS_Phaeodactylum_tricornutum
Cyto. (Gillian et al.)
Diatoms
Oomy_556492_argS_Phytophthora_sojae
Oomy_47611_argS_Phytophthora_ramorum
Oomycetes
Kineto_71668488_argS_Trypanosoma_cruzi
Kineto_71754737_argS_Trypanosoma_brucei
Kineto_146090829_argS_Leishmania_infantum
Kinetoplastida
Hapto_418599_argS_Emiliania_huxleyi
Haptophyta
Crypto_159514_argS_Guillardia_theta
Cyto. (Gillian et al.)
Cryptophyta
Plant_016540371.1_argS_Capsicum_annuum
Plant_016540372.1_argS_Capsicum_annuum
Plant_OIS97880.1_argS_Nicotiana_attenuata
Plant_011074326.1_argS_Sesamum_indicum
Plant_011083561.1_argS_Sesamum_indicum
Plant_AHB32107.1_argS_Camellia_sinensis
Plant_9757672_argS_Oryza_sativa
Plant_55770437_argS_Oryza_sativa
Plant_50932229_argS_Oryza_sativa
Plant_JAT67728.1_argS_Anthurium_amnicola
Plant_AT1G66530.1_argS_Arabidopsis_thaliana
Cyto. (SUBA3)
Plant_AT4G26300.1_argS_Arabidopsis_thaliana
Plas. (SUBA3)
Plant_008242477.1_argS_Prunus_mume
Plant_KNA24272.1_argS_Spinacia_oleracea
Plant_92890568_argS_Medicago_truncatula
Plant_92871912_argS_Medicago_truncatula
Green Plants
Rhiza_36494_argS_Bigelowiella_natans
Rhizaria
Algae_011399829.1_argS_Auxenochlorella_protothecoides
Algae_693501098_argS_Ostreococcus_tauri
Green Algae
Mam_ENSG00000113643_argS_Homo_sapiens
Nucl. (GO)
Unknown
Mam_56205734_argS_Mus_musculus
Animal_53734470_argS_Danio_rerio
Animal_40787810_argS_Xenopus_tropicalis
Animal_15789291_argS_Caenorhabditis_elegans
Opisthokonta
Oomy_73557_argS_Phytophthora_ramorum
Oomy_482167_argS_Phytophthora_sojae
Oomycetes
Amoeb_66806687_argS_Dictyostelium_discoideum
Amoebozoa
Chromer_19912_argS_Vitrella_brassicaformis
Mito. (mitopro: 0.9)
Chromer_3008_argS_Chromera_velia
Nucl. (Wolf PSORT: 15)
Chromerids
Heterok_299471588_argS_Ectocarpus_siliculosus
Heterokonta
Crypto_67389_argS_Guillardia_theta
Plas.|Mito. (Gillian et al.)
Cryptophyta
Diat_185193_argS_Fragilariopsis_cylindrus
Diat_36013_argS_Phaeodactylum_tricornutum
Plas.|Mito. (Gillian et al.)
Diat_40028_argS_Thalassiosira_pseudonana
Plas.|Mito. (Gillian et al.)
Diatoms
Heterok_22930_argS_Aureococcus_anophagefferens
stramenopiles
Hapto_426871_argS_Emiliania_huxleyi
Haptophyta
Rhodo_005708392_argS_Galdieria_sulphuraria
Rhodo_005702545_argS_Galdieria_sulphuraria
Rhodo_005713863_argS_Chondrus_crispus
Rhodo_544214343_argS_Cyanidioschyzon_merolae
Rhodophyta
Cyano_YP_007052635.1_argS_Nostoc_sp._PCC_7107
Cyano_YP_324062.1_argS_Anabaena_variabilis_ATCC_29413
Cyano_YP_007058199.1_argS_Rivularia_sp._PCC_7116
Cyano_YP_002370772.1_argS_Cyanothece_sp._PCC_8801
Cyano_YP_007107768.1_argS_Geitlerinema_sp._PCC_7407
Cyanobacteria
Bact_Gamma_YP_008045435.1_argS_Aeromonas_hydrophila_ML09-119
Bact_Gamma_YP_001651090.1_argS_Actinobacillus_pleuropneumoniae_serovar_3_str._JL03
Bact_Gamma_YP_008175302.1_argS_Alteromonas_macleodii_str._Ionian_Sea_U8
γ-protobacteria
Bact_Planctomycetes_YP_005446268.1_argS_Phycisphaera_mikurensis_NBRC_102666
Planctomycetes
Bact_Actino_YP_007870087.1_argS_Propionibacterium_avidum_44067
Bact_Actino_YP_007563282.1_argS_Ilumatobacter_coccineus_YM16-304
Actinobacteria
Bact_Planctomycetes_YP_004268094.1_argS_Planctomyces_brasiliensis_DSM_5305
Bact_Planctomycetes_YP_003629371.1_argS_Planctomyces_limnophilus_DSM_3776
Planctomycetes
Bact_Chlamydiae_YP_001878757.1_argS_Akkermansia_muciniphila_ATCC_BAA-835
Bact_Chlamydiae_YP_001819448.1_argS_Opitutus_terrae_PB90-1
Bact_Chlamydiae_YP_003549781.1_argS_Coraliomargarita_akajimensis_DSM_45221
Chlamydiae
Bact_Fusobacteria_YP_003310408.1_argS_Sebaldella_termitidis_ATCC_33386
Bact_Fusobacteria_YP_003164830.1_argS_Leptotrichia_buccalis_C-1013-b
Bact_Fusobacteria_YP_008476335.1_argS_Fusobacterium_nucleatum_subsp._vincentii_3_1_36A2
Fusobacteria
Bact_Chloroflexi_YP_002463911.1_argS_Chloroflexus_aggregans_DSM_9485
Chloroflexus
Mam_ENSG00000146282_argS_Homo_sapiens
Mito. (mitopro: 0.8)
Cyto. (GO)
Mam_009449802.1_argS_Pan_troglodytes
Mam_001128754.1_argS_Pongo_abelii
Mam_005552461.1_argS_Macaca_fascicularis
Mam_017718004.1_argS_Rhinopithecus_bieti
Mam_020945502.1_argS_Sus_scrofa
Mam_240120047_argS_Mus_musculus
Animal_148224766_argS_Xenopus_laevis
Animal_34784878_argS_Danio_rerio
Fung_19173381_argS_Encephalitozoon_cuniculi
Fung_71002110_argS_Aspergillus_fumigatus
fung_85092218_argS_Neurospora_crassa
Fung_6321883_argS_Saccharomyces_cerevisiae
Fung_6320548_argS_Saccharomyces_cerevisiae
fung_3738212_argS_Schizosaccharomyces_pombe
Opisthokonta
Amoeb_66823143_argS_Dictyostelium_discoideum
Amoeb_281203852_argS_Polysphondylium_pallidum
Amoebozoa
Archae_Nano_NP_963499.1_argS_Nanoarchaeum_equitans_Kin4-M
Nanoarchaeota
Bact_Firmicutes_YP_698948.1_argS_Clostridium_perfringens_SM101
Bact_Firmicutes_YP_008911607.1_argS_Paenibacillus_polymyxa_CR1
Firmicutes
Bact_Tenericutes_YP_001620480.1_argS_Acholeplasma_laidlawii_PG-8A
Tenericutes
Chromer_7363_argS_Chromera_velia
Cyto.|Nucl. (Wolf PSORT: 11)
Chromerids
Rhiza_50244_argS_Bigelowiella_natans
Rhizaria
Bact_Delta_YP_005035092.1_argS_Bacteriovorax_marinus_SJ
δ-protobacteria
Bact_Chlorobi_YP_213783.1_argS_Bacteroides_fragilis_NCTC_9343
Bact_Chlorobi_YP_005007865.1_argS_Niastella_koreensis_GR20-10
Bacteroidetes
Bact_Alpha_YP_001234086.1_argS_Acidiphilium_cryptum_JF-5
Bact_Alpha_YP_003188612.1_argS_Acetobacter_pasteurianus_IFO_3283-01
Bact_Alpha_YP_002563618.1_argS_Anaplasma_marginale_str._Florida
Bact_Alpha_YP_002544310.1_argS_Agrobacterium_radiobacter_K84
α-protobacteria
Bact_Delta_YP_002492835.1_argS_Anaeromyxobacter_dehalogenans_2CP-1
δ-protobacteria
Bact_Beta_YP_004232965.1_argS_Acidovorax_avenae_subsp._avenae_ATCC_19860
Bact_Beta_YP_004128677.1_argS_Alicycliphilus_denitrificans_BC
Bact_Beta_YP_003976205.1_argS_Achromobacter_xylosoxidans_A8
β-protobacteria
Bact_Gemmati_YP_002760979.1_argS_Gemmatimonas_aurantiaca_T-27
Gemmatimonadetes
Bact_Nitrospirae_YP_003798904.1_argS_Candidatus_Nitrospira_defluvii
Nitrospirae
Bact_Firmicutes_YP_006911916.1_argS_Dehalobacter_sp._DCA
Firmicutes
Bact_Chloroflexi_YP_005442158.1_argS_Caldilinea_aerophila_DSM_14535_=_NBRC_104270
Chloroflexi
Bact_Actino_YP_008664178.1_argS_Adlercreutzia_equolifaciens_DSM_19450
Bact_Actino_YP_003800742.1_argS_Olsenella_uli_DSM_7084
Actinobacteria
Bact_Synergistetes_YP_003317166.1_argS_Thermanaerovibrio_acidaminovorans_DSM_6589
Bact_Synergistetes_YP_003554265.1_argS_Aminobacterium_colombiense_DSM_12261
Synergistetes
Bact_Chrysiogenetes_YP_004113500.1_argS_Desulfurispirillum_indicum_S5
Chrysiogenetes
Bact_Elusimicrobia_YP_001875789.1_argS_Elusimicrobium_minutum_Pei191
Elusimicrobia
Bact_Deferribacteres_YP_003496576.1_argS_Deferribacter_desulfuricans_SSM1
Bact_Deferribacteres_YP_003503823.1_argS_Denitrovibrio_acetiphilus_DSM_12809
Bact_Deferribacteres_YP_004603552.1_argS_Flexistipes_sinusarabici_DSM_4947
Deferribacteres
Bact_Nitrospirae_YP_006766360.1_argS_Leptospirillum_ferriphilum_ML-04
Nitrospirae
Bact_Thermodesulfo_YP_004625651.1_argS_Thermodesulfatator_indicus_DSM_15286
Bact_Thermodesulfo_YP_004628534.1_argS_Thermodesulfobacterium_geofontis_OPF15
Thermodesulfobacteria
Bact_Nitrospirae_YP_002248471.1_argS_Thermodesulfovibrio_yellowstonii_DSM_11347
Nitrospirae
Bact_Dictyoglomi_YP_002251195.1_argS_Dictyoglomus_thermophilum_H-6-12
Bact_Dictyoglomi_YP_002353374.1_argS_Dictyoglomus_turgidum_DSM_6724
Dictyoglomi
Bact_Aquificae_YP_003473062.1_argS_Thermocrinis_albus_DSM_14484
Bact_Aquificae_YP_002121082.1_argS_Hydrogenobaculum_sp._Y04AAS1
Bact_Aquificae_YP_001930790.1_argS_Sulfurihydrogenibium_sp._YO3AOP1
Aquificae
Bact_Caldiserica_YP_005472660.1_argS_Caldisericum_exile_AZM16c01
Caldiserica
Bact_Thermotogae_YP_001409927.1_argS_Fervidobacterium_nodosum_Rt17-B1
Bact_Thermotogae_YP_001306725.1_argS_Thermosipho_melanesiensis_BI429
Bact_Thermotogae_YP_002941130.1_argS_Kosmotoga_olearia_TBF_19.5.1
Thermotogae
Archae_Eury_YP_003482935.1_argS_Aciduliprofundum_boonei_T469
Archae_Eury_YP_007246540.1_argS_Aciduliprofundum_sp._MAR08-339
Archae_Kor_YP_001736656.1_argS_Candidatus_Korarchaeum_cryptofilum_OPF8
Bact_Crenarchaeota_YP_007174903.1_argS_Caldisphaera_lagunensis_DSM_15908
Archaea
Algae_0800010133_argS_Ostreococcus_tauri
Green Algae
Excav_123387375_argS_Trichomonas_vaginalis
Excavata
Bact_Thermus_YP_004203028.1_argS_Thermus_scotoductus_SA-01
Bact_Thermus_YP_004058181.1_argS_Oceanithermus_profundus_DSM_14977
Bact_Thermus_YP_002785743.2_argS_Deinococcus_deserti_VCD115
Thermus
Bact_Acidobacteria_YP_006421081.1_argS_Terriglobus_roseus_DSM_18391
Acidobacteria
Archae_Thaum_YP_001582020.1_argS_Nitrosopumilus_maritimus_SCM1
Archae_Eury_NP_143342.1_argS_Pyrococcus_horikoshii_OT3
Archaea
0.5

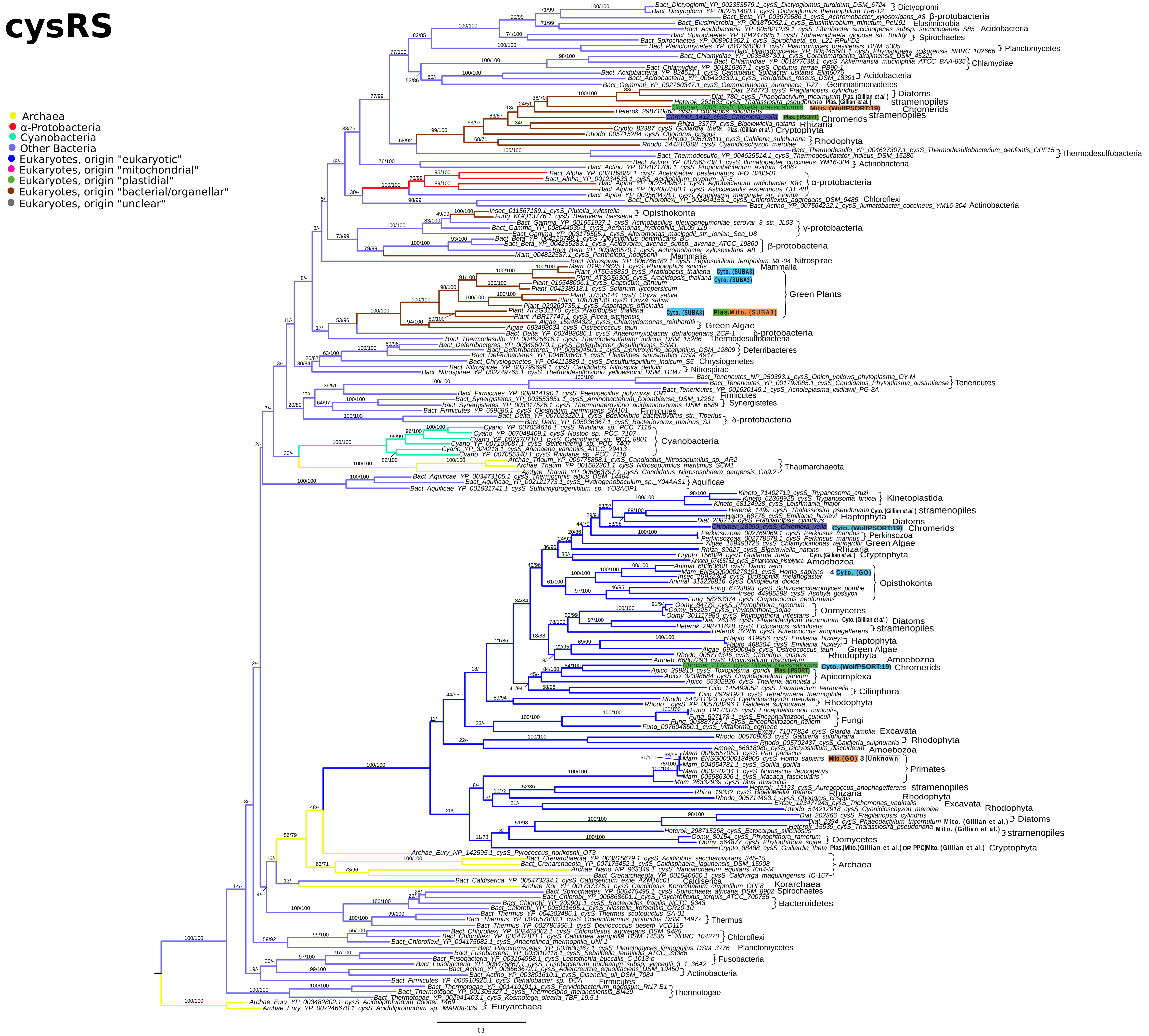
cysRS
Archaea
α-Protobacteria
Cyanobacteria
Other Bacteria
Eukaryotes, origin "eukaryotic"
Eukaryotes, origin "mitochondrial"
Eukaryotes, origin "plastidial"
Eukaryotes, origin "bacterial/organellar"
Eukaryotes, origin "unclear"
Dictyoglomi
β-protobacteria
Elusimicrobia
Acidobacteria
Spirochaetes
Planctomycetes
Chlamydiae
Gemmatimonadetes
Diatoms
stramenopiles
Chromerids
Rhizaria
Cryptophyta
Rhodophyta
Thermodesulfobacteria
Actinobacteria
α-protobacteria
Chloroflexi
Opisthokonta
γ-protobacteria
Mammalia
Nitrospirae
Green Plants
Green Algae
δ-protobacteria
Deferribacteres
Chrysiogenetes
Tenericutes
Firmicutes
Synergistetes
Cyanobacteria
Thaumarchaeota
Aquificae
Kinetoplastida
Haptophyta
Perkinsozoa
Amoebozoa
Oomycetes
Apicomplexa
Ciliophora
Fungi
Excavata
Primates
Archaea
Korarchaeota
Caldiserica
Bacteroidetes
Thermus
Fusobacteria
Thermotogae
Euryarchaea
Cyto. (SUBA3)
Plas. Mito. (SUBA3)
Mito. (WolfPSORT:19)
Cyto. (WolfPSORT:19)
Plas. (iPSORT)
Cyto. (GO)
Mito. (GO)
Unknown
Mito. (Gillian et al.)
Plas. (Gillian et al.)
Cyto. (Gillian et al.)
0.3

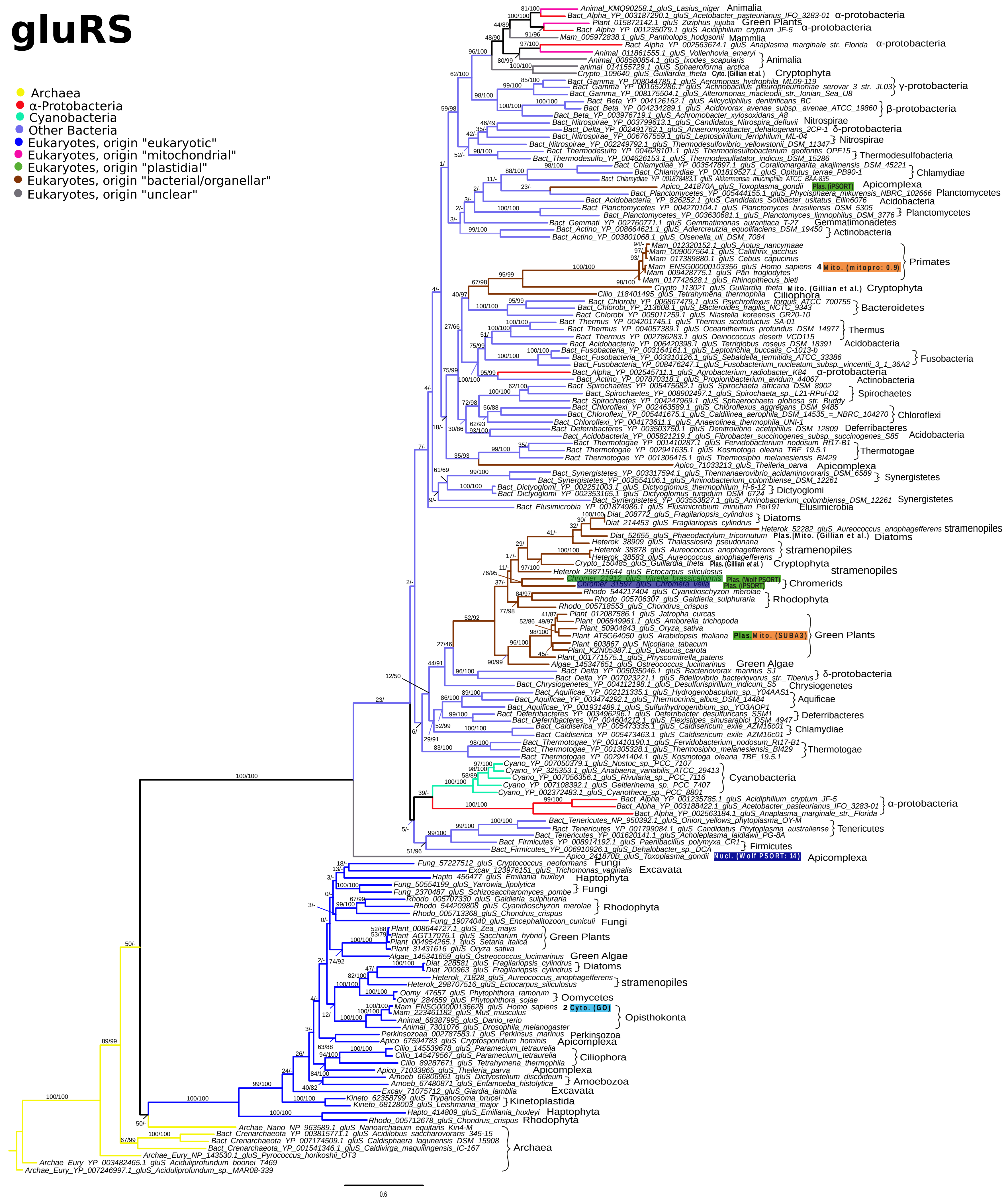
gluRS
Archaea
α-Protobacteria
Cyanobacteria
Other Bacteria
Eukaryotes, origin "eukaryotic"
Eukaryotes, origin "mitochondrial"
Eukaryotes, origin "plastidial"
Eukaryotes, origin "bacterial/organellar"
Eukaryotes, origin "unclear"
Animalia
α-protobacteria
Green Plants
Mammlia
Cryptophyta
γ-protobacteria
β-protobacteria
Nitrospirae
δ-protobacteria
Thermodesulfobacteria
Chlamydiae
Apicomplexa
Planctomycetes
Acidobacteria
Gemmatimonadetes
Actinobacteria
Primates
Ciliophora
Bacteroidetes
Thermus
Fusobacteria
Spirochaetes
Chloroflexi
Deferribacteres
Thermotogae
Synergistetes
Dictyoglomi
Elusimicrobia
Diatoms
stramenopiles
Chromerids
Rhodophyta
Green Algae
Chrysiogenetes
Aquificae
Cyanobacteria
Tenericutes
Firmicutes
Fungi
Excavata
Haptophyta
Oomycetes
Opisthokonta
Perkinsozoa
Amoebozoa
Kinetoplastida
Archaea
Cyto. (Gillian et al.)
Mito. (Gillian et al.)
Plas. (iPSORT)
Plas. (Wolf PSORT)
Plas.|Mito. (Gillian et al.)
Plas. (Gillian et al.)
Plas.Mito. (SUBA3)
4 Mito. (mitopro: 0.9)
Nucl. (Wolf PSORT: 14)
2 Cyto. (GO)
0.6

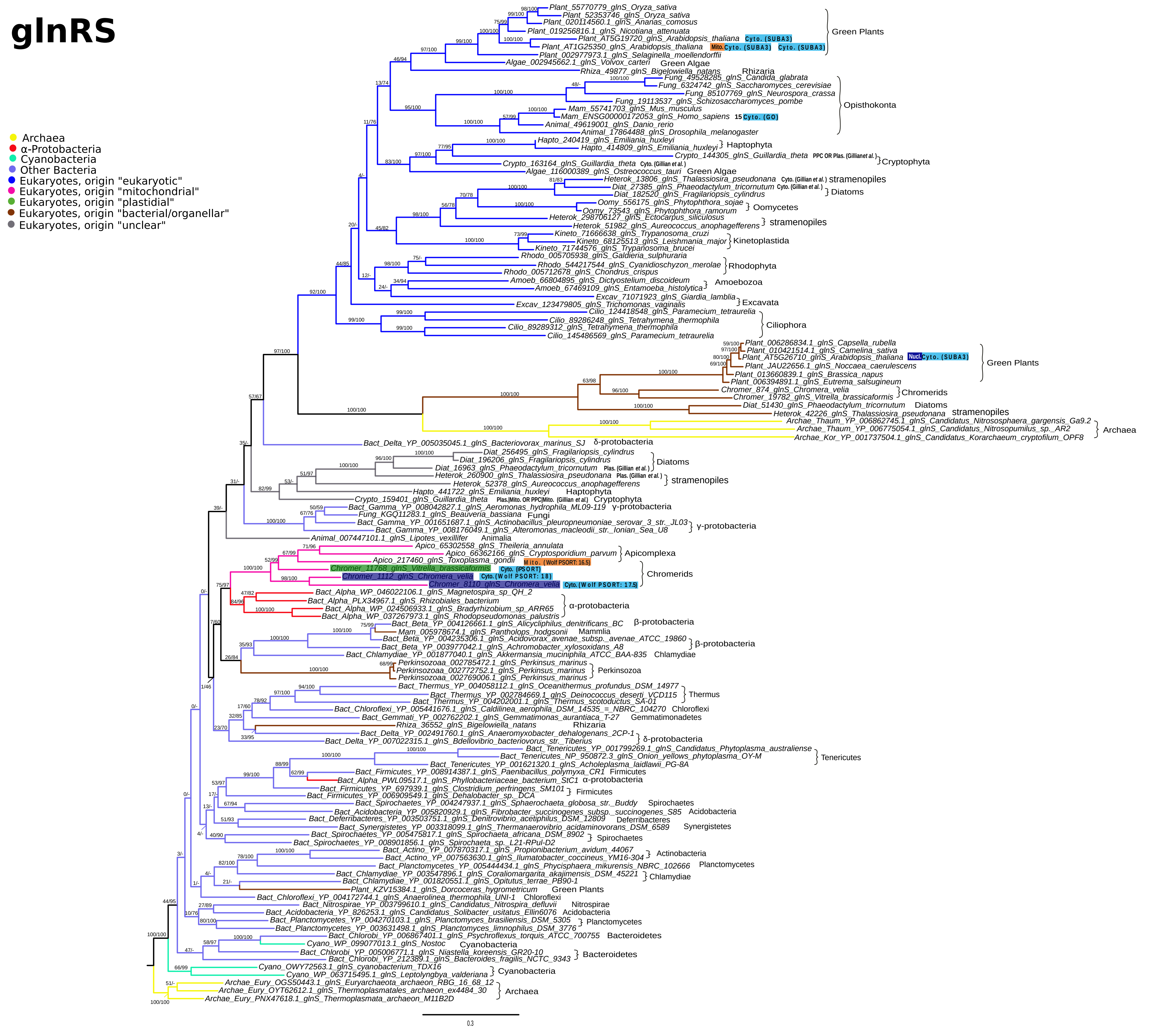

glnRS
Archaea
α-Protobacteria
Cyanobacteria
Other Bacteria
Eukaryotes, origin "eukaryotic"
Eukaryotes, origin "mitochondrial"
Eukaryotes, origin "plastidial"
Eukaryotes, origin "bacterial/organellar"
Eukaryotes, origin "unclear"
Plant_55770779_glnS_Oryza_sativa
Plant_52353746_glnS_Oryza_sativa
Plant_020114560.1_glnS_Ananas_comosus
Plant_019256816.1_glnS_Nicotiana_attenuata
Plant_AT5G19720_glnS_Arabidopsis_thaliana Cyto. (SUBA3)
Plant_AT1G25350_glnS_Arabidopsis_thaliana Mito. Cyto. (SUBA3) Cyto. (SUBA3)
Plant_002977973.1_glnS_Selaginella_moellendorffii
Green Plants
Algae_002945662.1_glnS_Volvox_carteri
Green Algae
Rhiza_49877_glnS_Bigelowiella_natans
Rhizaria
Fung_49528285_glnS_Candida_glabrata
Fung_6324742_glnS_Saccharomyces_cerevisiae
Fung_85107769_glnS_Neurospora_crassa
Fung_19113537_glnS_Schizosaccharomyces_pombe
Mam_55741703_glnS_Mus_musculus
Mam_ENSG00000172053_glnS_Homo_sapiens 15 Cyto. (GO)
Animal_49619001_glnS_Danio_rerio
Animal_17864488_glnS_Drosophila_melanogaster
Opisthokonta
Hapto_240419_glnS_Emiliania_huxleyi
Hapto_414809_glnS_Emiliania_huxleyi
Haptophyta
Crypto_144305_glnS_Guillardia_theta PPC OR Plas. (Gillian et al.)
Crypto_163164_glnS_Guillardia_theta Cyto. (Gillian et al.)
Cryptophyta
Algae_116000389_glnS_Ostreococcus_tauri
Green Algae
Heterok_13806_glnS_Thalassiosira_pseudonana Cyto. (Gillian et al.)
stramenopiles
Diat_27385_glnS_Phaeodactylum_tricornutum Cyto. (Gillian et al.)
Diat_182520_glnS_Fragilariopsis_cylindrus
Diatoms
Oomy_556175_glnS_Phytophthora_sojae
Oomy_73543_glnS_Phytophthora_ramorum
Oomycetes
Heterok_298706127_glnS_Ectocarpus_siliculosus
Heterok_51982_glnS_Aureococcus_anophagefferens
stramenopiles
Kineto_71666638_glnS_Trypanosoma_cruzi
Kineto_68125513_glnS_Leishmania_major
Kineto_71744576_glnS_Trypanosoma_brucei
Kinetoplastida
Rhodo_005705938_glnS_Galdieria_sulphuraria
Rhodo_544217544_glnS_Cyanidioschyzon_merolae
Rhodo_005712678_glnS_Chondrus_crispus
Rhodophyta
Amoeb_66804895_glnS_Dictyostelium_discoideum
Amoeb_67469109_glnS_Entamoeba_histolytica
Amoebozoa
Excav_71071923_glnS_Giardia_lamblia
Excav_123479805_glnS_Trichomonas_vaginalis
Excavata
Cilio_124418548_glnS_Paramecium_tetraurelia
Cilio_89286248_glnS_Tetrahymena_thermophila
Cilio_89289312_glnS_Tetrahymena_thermophila
Cilio_145486569_glnS_Paramecium_tetraurelia
Ciliophora
Plant_006286834.1_glnS_Capsella_rubella
Plant_010421514.1_glnS_Camelina_sativa
Plant_AT5G26710_glnS_Arabidopsis_thaliana Nucl. Cyto. (SUBA3)
Plant_JAU22656.1_glnS_Noccaea_caerulescens
Plant_013660839.1_glnS_Brassica_napus
Plant_006394891.1_glnS_Eutrema_salsugineum
Green Plants
Chromer_874_glnS_Chromera_velia
Chromer_19782_glnS_Vitrella_brassicaformis
Chromerids
Diat_51430_glnS_Phaeodactylum_tricornutum
Diatoms
Heterok_42226_glnS_Thalassiosira_pseudonana
stramenopiles
Archae_Thaum_YP_006862745.1_glnS_Candidatus_Nitrososphaera_gargensis_Ga9.2
Archae_Thaum_YP_006775054.1_glnS_Candidatus_Nitrosopumilus_sp._AR2
Archae_Kor_YP_001737504.1_glnS_Candidatus_Korarchaeum_cryptofilum_OPF8
Archaea
Bact_Delta_YP_005035045.1_glnS_Bacteriovorax_marinus_SJ
δ-protobacteria
Diat_256495_glnS_Fragilariopsis_cylindrus
Diat_196206_glnS_Fragilariopsis_cylindrus
Diat_16963_glnS_Phaeodactylum_tricornutum Plas. (Gillian et al.)
Diatoms
Heterok_260900_glnS_Thalassiosira_pseudonana Plas. (Gillian et al.)
Heterok_52378_glnS_Aureococcus_anophagefferens
stramenopiles
Hapto_441722_glnS_Emiliania_huxleyi
Haptophyta
Crypto_159401_glnS_Guillardia_theta Plas.|Mito. OR PPC|Mito. (Gillian et al.)
Cryptophyta
Bact_Gamma_YP_008042827.1_glnS_Aeromonas_hydrophila_ML09-119
γ-protobacteria
Fung_KGQ11283.1_glnS_Beauveria_bassiana
Fungi
Bact_Gamma_YP_001651687.1_glnS_Actinobacillus_pleuropneumoniae_serovar_3_str._JL03
Bact_Gamma_YP_008176049.1_glnS_Alteromonas_macleodii_str._Ionian_Sea_U8
γ-protobacteria
Animal_007447101.1_glnS_Lipotes_vexillifer
Animalia
Apico_65302558_glnS_Theileria_annulata
Apico_66362166_glnS_Cryptosporidium_parvum
Apico_217460_glnS_Toxoplasma_gondii
Apicomplexa
Chromer_11768_glnS_Vitrella_brassicaformis Mito. (Wolf PSORT: 16.5) Cyto. (iPSORT)
Chromer_1112_glnS_Chromera_velia Cyto. (Wolf PSORT: 18)
Chromer_8110_glnS_Chromera_velia Cyto. (Wolf PSORT: 17.5)
Chromerids
Bact_Alpha_WP_046022106.1_glnS_Magnetospira_sp_QH_2
Bact_Alpha_PLX34967.1_glnS_Rhizobiales_bacterium
Bact_Alpha_WP_024506933.1_glnS_Bradyrhizobium_sp_ARR65
Bact_Alpha_WP_037267973.1_glnS_Rhodopseudomonas_palustris
α-protobacteria
Bact_Beta_YP_004126661.1_glnS_Alicycliphilus_denitrificans_BC
β-protobacteria
Mam_005978674.1_glnS_Pantholops_hodgsonii
Mammlia
Bact_Beta_YP_004235306.1_glnS_Acidovorax_avenae_subsp._avenae_ATCC_19860
Bact_Beta_YP_003977042.1_glnS_Achromobacter_xylosoxidans_A8
β-protobacteria
Bact_Chlamydiae_YP_001877040.1_glnS_Akkermansia_muciniphila_ATCC_BAA-835
Chlamydiae
Perkinsozoaa_002785472.1_glnS_Perkinsus_marinus
Perkinsozoaa_002772752.1_glnS_Perkinsus_marinus
Perkinsozoaa_002769006.1_glnS_Perkinsus_marinus
Perkinsozoa
Bact_Thermus_YP_004058112.1_glnS_Oceanithermus_profundus_DSM_14977
Bact_Thermus_YP_002784669.1_glnS_Deinococcus_deserti_VCD115
Bact_Thermus_YP_004202001.1_glnS_Thermus_scotoductus_SA-01
Thermus
Bact_Chloroflexi_YP_005441676.1_glnS_Caldilinea_aerophila_DSM_14535_=_NBRC_104270
Chloroflexi
Bact_Gemmati_YP_002762202.1_glnS_Gemmatimonas_aurantiaca_T-27
Gemmatimonadetes
Rhiza_36552_glnS_Bigelowiella_natans
Rhizaria
Bact_Delta_YP_002491760.1_glnS_Anaeromyxobacter_dehalogenans_2CP-1
Bact_Delta_YP_007022315.1_glnS_Bdellovibrio_bacteriovorus_str._Tiberius
δ-protobacteria
Bact_Tenericutes_YP_001799269.1_glnS_Candidatus_Phytoplasma_australiense
Bact_Tenericutes_NP_950872.3_glnS_Onion_yellows_phytoplasma_OY-M
Bact_Tenericutes_YP_001621320.1_glnS_Acholeplasma_laidlawii_PG-8A
Tenericutes
Bact_Firmicutes_YP_008914387.1_glnS_Paenibacillus_polymyxa_CR1
Firmicutes
Bact_Alpha_PWL09517.1_glnS_Phyllobacteriaceae_bacterium_StC1
α-protobacteria
Bact_Firmicutes_YP_697939.1_glnS_Clostridium_perfringens_SM101
Bact_Firmicutes_YP_006909549.1_glnS_Dehalobacter_sp._DCA
Firmicutes
Bact_Spirochaetes_YP_004247937.1_glnS_Sphaerochaeta_globosa_str._Buddy
Spirochaetes
Bact_Acidobacteria_YP_005820929.1_glnS_Fibrobacter_succinogenes_subsp._succinogenes_S85
Acidobacteria
Bact_Deferribacteres_YP_003503751.1_glnS_Denitrovibrio_acetiphilus_DSM_12809
Deferribacteres
Bact_Synergistetes_YP_003318099.1_glnS_Thermanaerovibrio_acidaminovorans_DSM_6589
Synergistetes
Bact_Spirochaetes_YP_005475817.1_glnS_Spirochaeta_africana_DSM_8902
Bact_Spirochaetes_YP_008901856.1_glnS_Spirochaeta_sp._L21-RPul-D2
Spirochaetes
Bact_Actino_YP_007870317.1_glnS_Propionibacterium_avidum_44067
Bact_Actino_YP_007563630.1_glnS_Ilumatobacter_coccineus_YM16-304
Actinobacteria
Bact_Planctomycetes_YP_005444434.1_glnS_Phycisphaera_mikurensis_NBRC_102666
Planctomycetes
Bact_Chlamydiae_YP_003547896.1_glnS_Coraliomargarita_akajimensis_DSM_45221
Bact_Chlamydiae_YP_001820551.1_glnS_Opitutus_terrae_PB90-1
Chlamydiae
Plant_KZV15384.1_glnS_Dorcoceras_hygrometricum
Green Plants
Bact_Chloroflexi_YP_004172744.1_glnS_Anaerolinea_thermophila_UNI-1
Chloroflexi
Bact_Nitrospirae_YP_003799610.1_glnS_Candidatus_Nitrospira_defluvii
Nitrospirae
Bact_Acidobacteria_YP_826253.1_glnS_Candidatus_Solibacter_usitatus_Ellin6076
Acidobacteria
Bact_Planctomycetes_YP_004270103.1_glnS_Planctomyces_brasiliensis_DSM_5305
Bact_Planctomycetes_YP_003631498.1_glnS_Planctomyces_limnophilus_DSM_3776
Planctomycetes
Bact_Chlorobi_YP_006867401.1_glnS_Psychroflexus_torquis_ATCC_700755
Bacteroidetes
Cyano_WP_099077013.1_glnS_Nostoc
Cyanobacteria
Bact_Chlorobi_YP_005006771.1_glnS_Niastella_koreensis_GR20-10
Bact_Chlorobi_YP_212389.1_glnS_Bacteroides_fragilis_NCTC_9343
Bacteroidetes
Cyano_OWY72563.1_glnS_cyanobacterium_TDX16
Cyano_WP_063715495.1_glnS_Leptolyngbya_valderiana
Cyanobacteria
Archae_Eury_OGS50443.1_glnS_Euryarchaeota_archaeon_RBG_16_68_12
Archae_Eury_OYT62612.1_glnS_Thermoplasmatales_archaeon_ex4484_30
Archae_Eury_PNX47618.1_glnS_Thermoplasmata_archaeon_M11B2D
Archaea
0.3

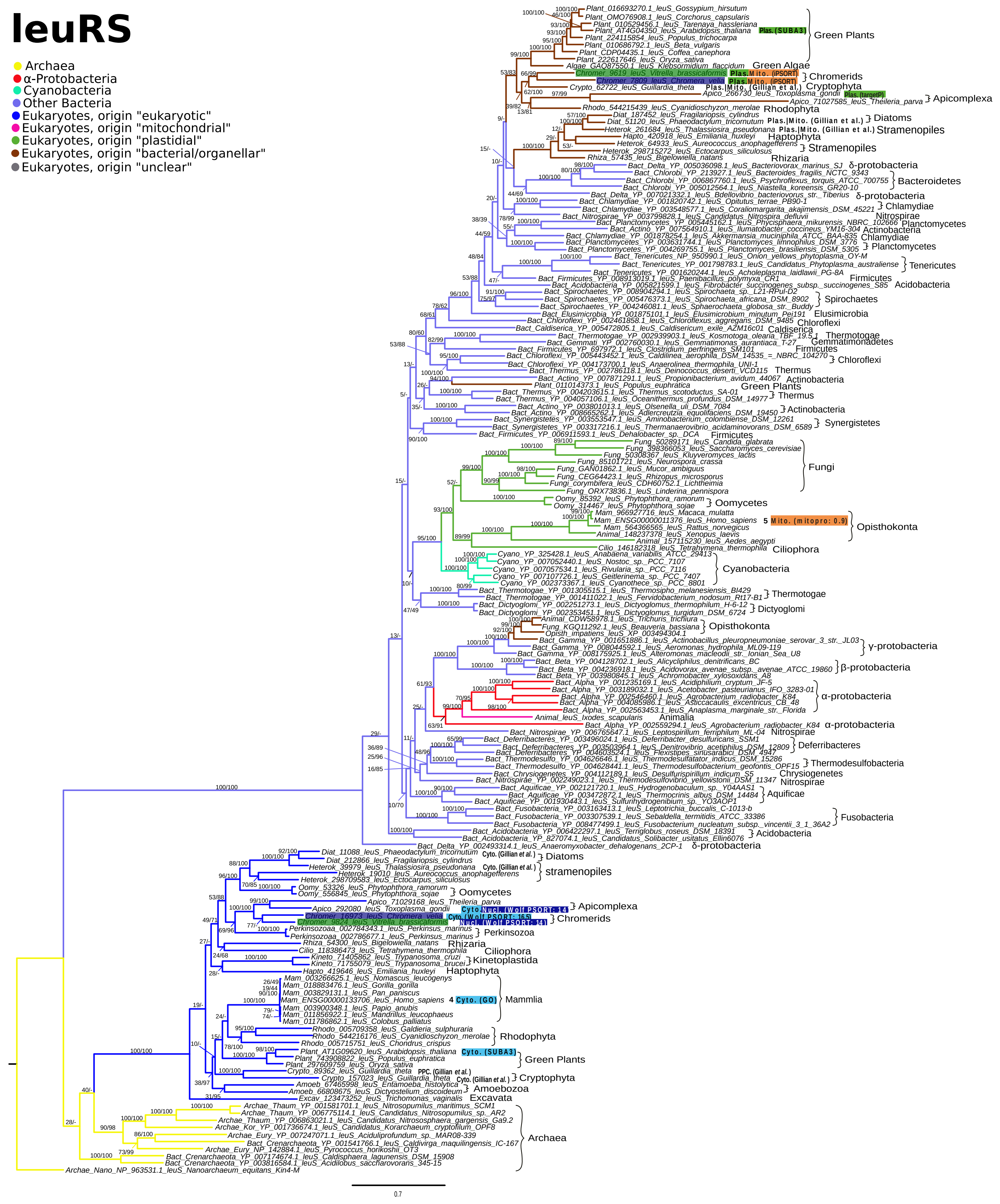

leuRS
Archaea
α-Protobacteria
Cyanobacteria
Other Bacteria
Eukaryotes, origin "eukaryotic"
Eukaryotes, origin "mitochondrial"
Eukaryotes, origin "plastidial"
Eukaryotes, origin "bacterial/organellar"
Eukaryotes, origin "unclear"
Green Plants
Green Algae
Chromerids
Cryptophyta
Apicomplexa
Rhodophyta
Diatoms
Stramenopiles
Haptophyta
Rhizaria
δ-protobacteria
Bacteroidetes
Chlamydiae
Nitrospirae
Planctomycetes
Actinobacteria
Tenericutes
Firmicutes
Acidobacteria
Spirochaetes
Elusimicrobia
Chloroflexi
Caldiserica
Thermotogae
Gemmatimonadetes
Thermus
Synergistetes
Fungi
Oomycetes
Opisthokonta
Ciliophora
Cyanobacteria
Dictyoglomi
γ-protobacteria
β-protobacteria
α-protobacteria
Animalia
Deferribacteres
Thermodesulfobacteria
Chrysiogenetes
Aquificae
Fusobacteria
Perkinsozoa
Kinetoplastida
Mammlia
Amoebozoa
Excavata
Archaea
0.7

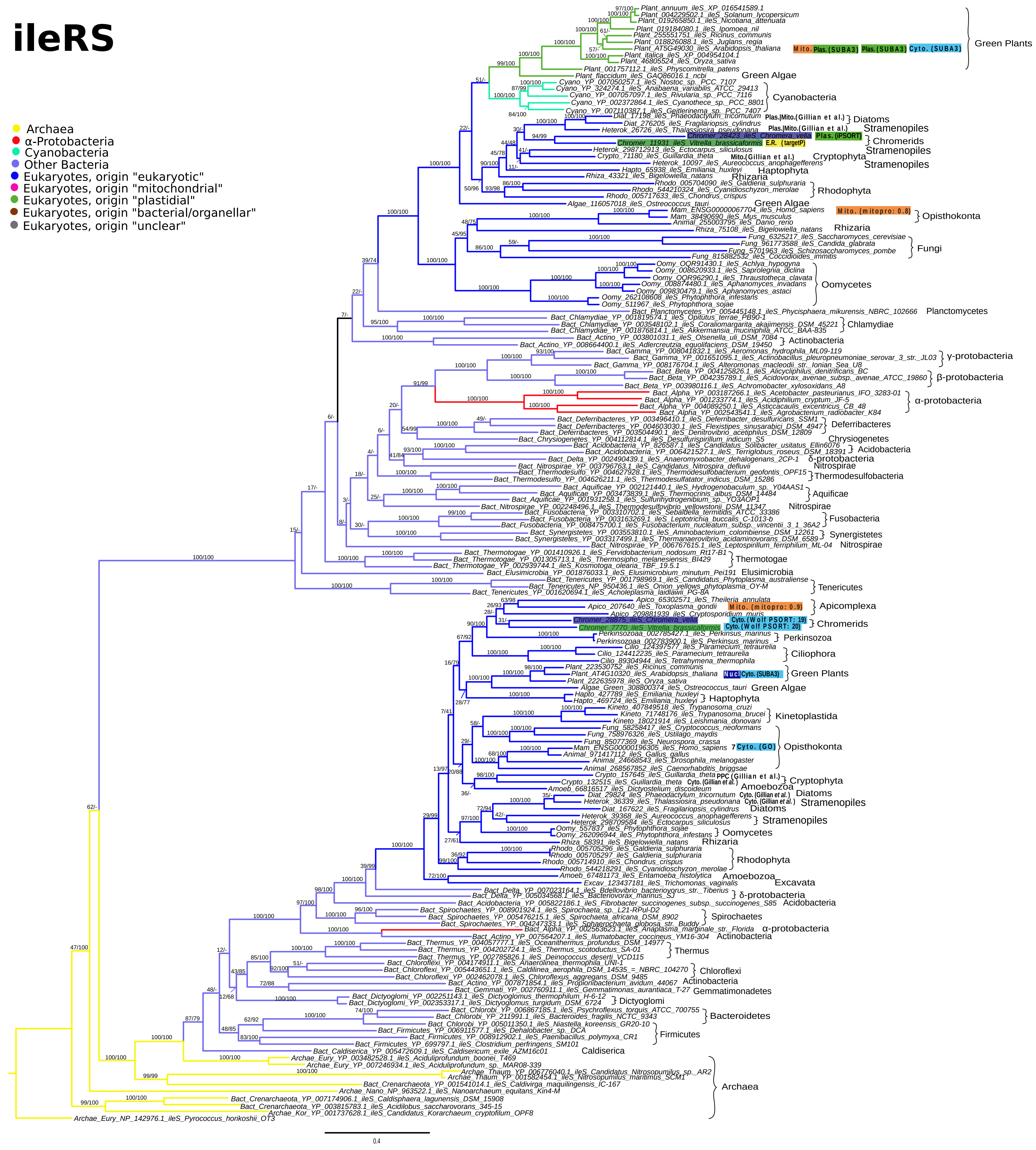
ileRS
Archaea
α-Protobacteria
Cyanobacteria
Other Bacteria
Eukaryotes, origin "eukaryotic"
Eukaryotes, origin "mitochondrial"
Eukaryotes, origin "plastidial"
Eukaryotes, origin "bacterial/organellar"
Eukaryotes, origin "unclear"
Green Plants
Green Algae
Cyanobacteria
Diatoms
Stramenopiles
Chromerids
Cryptophyta
Haptophyta
Rhizaria
Rhodophyta
Opisthokonta
Fungi
Oomycetes
Planctomycetes
Chlamydiae
Actinobacteria
γ-protobacteria
β-protobacteria
α-protobacteria
Deferribacteres
Chrysiogenetes
Acidobacteria
δ-protobacteria
Nitrospirae
Thermodesulfobacteria
Aquificae
Fusobacteria
Synergistetes
Thermotogae
Elusimicrobia
Tenericutes
Apicomplexa
Perkinsozoa
Ciliophora
Kinetoplastida
Amoebozoa
Excavata
Spirochaetes
Thermus
Chloroflexi
Gemmatimonadetes
Dictyoglomi
Bacteroidetes
Firmicutes
Caldiserica
Archaea
0.4

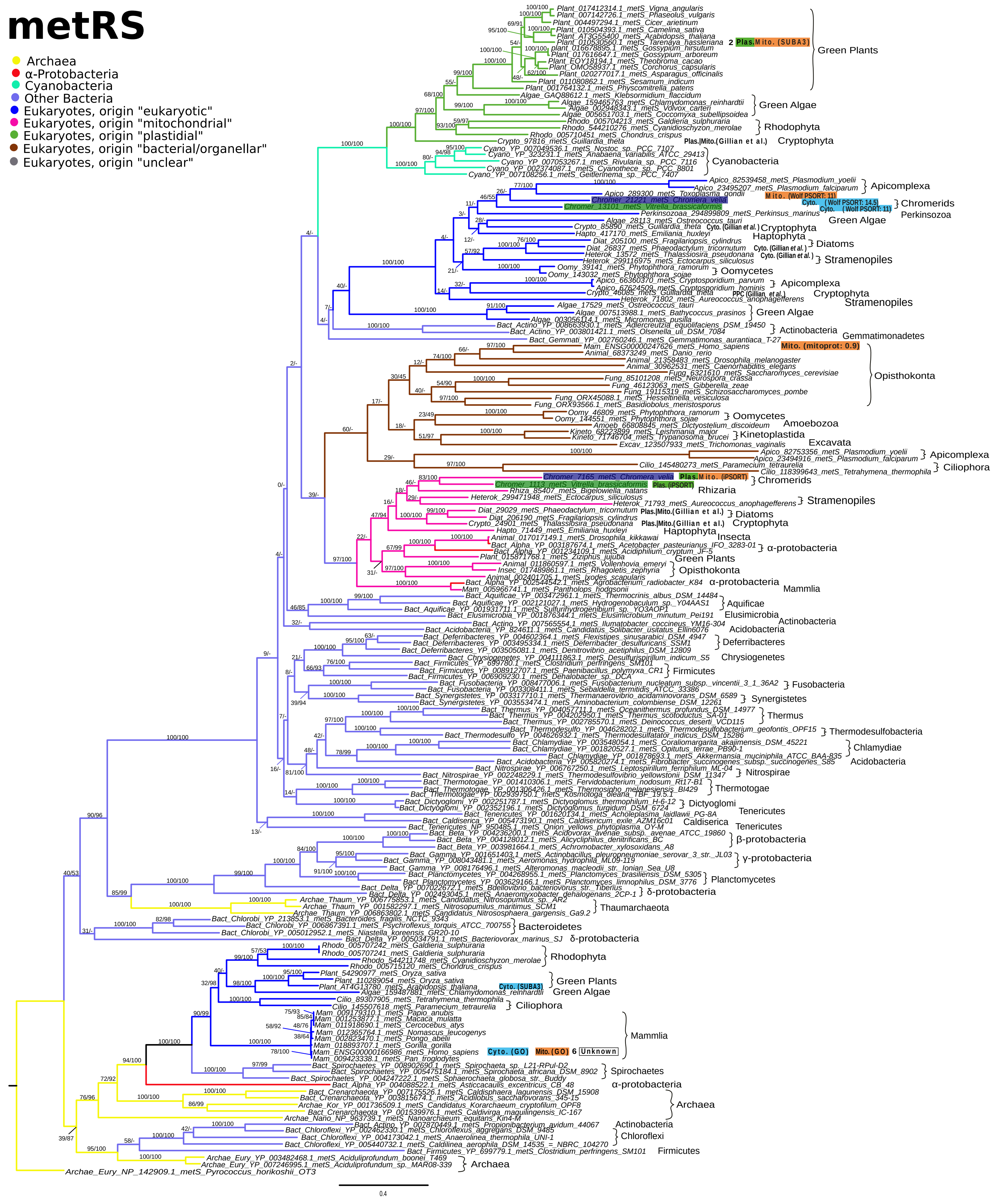

metRS
Archaea
α-Protobacteria
Cyanobacteria
Other Bacteria
Eukaryotes, origin "eukaryotic"
Eukaryotes, origin "mitochondrial"
Eukaryotes, origin "plastidial"
Eukaryotes, origin "bacterial/organellar"
Eukaryotes, origin "unclear"
Plant_017412314.1_metS_Vigna_angularis
Plant_007142726.1_metS_Phaseolus_vulgaris
Plant_004497294.1_metS_Cicer_arietinum
Plant_010504393.1_metS_Camelina_sativa
Plant_AT3G55400_metS_Arabidopsis_thaliana
Plant_010530560.1_metS_Tarenaya_hassleriana
2 Plas.Mito. (SUBA3)
plant_016678895.1_metS_Gossypium_hirsutum
Plant_017616647.1_metS_Gossypium_arboreum
Plant_EOY18194.1_metS_Theobroma_cacao
Plant_OMO58937.1_metS_Corchorus_capsularis
Plant_020277017.1_metS_Asparagus_officinalis
Plant_011080862.1_metS_Sesamum_indicum
Plant_001764132.1_metS_Physcomitrella_patens
Green Plants
Algae_GAQ88612.1_metS_Klebsormidium_flaccidum
Algae_159465763_metS_Chlamydomonas_reinhardtii
Algae_002948343.1_metS_Volvox_carteri
Algae_005651703.1_metS_Coccomyxa_subellipsoidea
Green Algae
Rhodo_005704213_metS_Galdieria_sulphuraria
Rhodo_544210276_metS_Cyanidioschyzon_merolae
Rhodo_005710451_metS_Chondrus_crispus
Rhodophyta
Crypto_97816_metS_Guillardia_theta
Plas.|Mito.(Gillian et al.)
Cryptophyta
Cyano_YP_007049536.1_metS_Nostoc_sp._PCC_7107
Cyano_YP_323231.1_metS_Anabaena_variabilis_ATCC_29413
Cyano_YP_007053267.1_metS_Rivularia_sp._PCC_7116
Cyano_YP_002374087.1_metS_Cyanothece_sp._PCC_8801
Cyano_YP_007108256.1_metS_Geitlerinema_sp._PCC_7407
Cyanobacteria
Apico_82539458_metS_Plasmodium_yoelii
Apico_23495207_metS_Plasmodium_falciparum
Apicomplexa
Apico_289300_metS_Toxoplasma_gondii
Mito. (Wolf PSORT: 11)
Chromer_21221_metS_Chromera_velia
Cyto. (Wolf PSORT: 14.5)
Chromer_13101_metS_Vitrella_brassicaformis
Cyto. (Wolf PSORT: 11)
Chromerids
Perkinsozoa_294899809_metS_Perkinsus_marinus
Perkinsozoa
Algae_28113_metS_Ostreococcus_tauri
Green Algae
Crypto_85890_metS_Guillardia_theta
Cyto. (Gillian et al.)
Cryptophyta
Hapto_417170_metS_Emiliania_huxleyi
Haptophyta
Diat_205100_metS_Fragilariopsis_cylindrus
Diat_26837_metS_Phaeodactylum_tricornutum
Cyto. (Gillian et al.)
Diatoms
Heterok_13572_metS_Thalassiosira_pseudonana
Cyto. (Gillian et al.)
Heterok_299116975_metS_Ectocarpus_siliculosus
Stramenopiles
Oomy_39141_metS_Phytophthora_ramorum
Oomy_143032_metS_Phytophthora_sojae
Oomycetes
Apico_66360370_metS_Cryptosporidium_parvum
Apico_67624509_metS_Cryptosporidium_hominis
Apicomplexa
Crypto_46085_metS_Guillardia_theta
PPC (Gillian et al.)
Cryptophyta
Heterok_71802_metS_Aureococcus_anophagefferens
Stramenopiles
Algae_17529_metS_Ostreococcus_tauri
Algae_007513988.1_metS_Bathycoccus_prasinos
Algae_003056114.1_metS_Micromonas_pusilla
Green Algae
Bact_Actino_YP_008663930.1_metS_Adlercreutzia_equolifaciens_DSM_19450
Bact_Actino_YP_003801421.1_metS_Olsenella_uli_DSM_7084
Actinobacteria
Bact_Gemmati_YP_002760246.1_metS_Gemmatimonas_aurantiaca_T-27
Gemmatimonadetes
Mam_ENSG00000247626_metS_Homo_sapiens
Mito. (mitoprot: 0.9)
Animal_68373249_metS_Danio_rerio
Animal_21358483_metS_Drosophila_melanogaster
Animal_30962531_metS_Caenorhabditis_elegans
Fung_6321610_metS_Saccharomyces_cerevisiae
Fung_85101208_metS_Neurospora_crassa
Fung_46123063_metS_Gibberella_zeae
Fung_19115319_metS_Schizosaccharomyces_pombe
Fung_ORX45088.1_metS_Hesseltinella_vesiculosa
Fung_ORX93566.1_metS_Basidiobolus_meristosporus
Opisthokonta
Oomy_46809_metS_Phytophthora_ramorum
Oomy_144551_metS_Phytophthora_sojae
Oomycetes
Amoeb_66808845_metS_Dictyostelium_discoideum
Amoebozoa
Kineto_68223899_metS_Leishmania_major
Kineto_71746704_metS_Trypanosoma_brucei
Kinetoplastida
Excav_123507933_metS_Trichomonas_vaginalis
Excavata
Apico_82753356_metS_Plasmodium_yoelii
Apico_23494916_metS_Plasmodium_falciparum
Apicomplexa
Cilio_145480273_metS_Paramecium_tetraurelia
Cilio_118399643_metS_Tetrahymena_thermophila
Ciliophora
Chromer_7165_metS_Chromera_velia
Plas.Mito. (iPSORT)
Chromer_1113_metS_Vitrella_brassicaformis
Plas. (iPSORT)
Chromerids
Rhiza_85407_metS_Bigelowiella_natans
Rhizaria
Heterok_299471948_metS_Ectocarpus_siliculosus
Heterok_71793_metS_Aureococcus_anophagefferens
Stramenopiles
Diat_29029_metS_Phaeodactylum_tricornutum
Plas.|Mito.(Gillian et al.)
Diat_206190_metS_Fragilariopsis_cylindrus
Diatoms
Crypto_24901_metS_Thalassiosira_pseudonana
Plas.|Mito.(Gillian et al.)
Cryptophyta
Hapto_71449_metS_Emiliania_huxleyi
Haptophyta
Animal_017017149.1_metS_Drosophila_kikkawai
Insecta
Bact_Alpha_YP_003187674.1_metS_Acetobacter_pasteurianus_IFO_3283-01
Bact_Alpha_YP_001234109.1_metS_Acidiphilium_cryptum_JF-5
α-protobacteria
Plant_015871768.1_metS_Ziziphus_jujuba
Green Plants
Animal_011860597.1_metS_Vollenhovia_emeryi
Insec_017489861.1_metS_Rhagoletis_zephyria
Animal_002401705.1_metS_Ixodes_scapularis
Opisthokonta
Bact_Alpha_YP_002544542.1_metS_Agrobacterium_radiobacter_K84
α-protobacteria
Mam_005966741.1_metS_Pantholops_hodgsonii
Mammlia
Bact_Aquificae_YP_003472961.1_metS_Thermocrinis_albus_DSM_14484
Bact_Aquificae_YP_002121027.1_metS_Hydrogenobaculum_sp._Y04AAS1
Bact_Aquificae_YP_001931711.1_metS_Sulfurihydrogenibium_sp._YO3AOP1
Aquificae
Bact_Elusimicrobia_YP_001876344.1_metS_Elusimicrobium_minutum_Pei191
Elusimicrobia
Bact_Actino_YP_007565554.1_metS_Ilumatobacter_coccineus_YM16-304
Actinobacteria
Bact_Acidobacteria_YP_824611.1_metS_Candidatus_Solibacter_usitatus_Ellin6076
Acidobacteria
Bact_Deferribacteres_YP_004602364.1_metS_Flexistipes_sinusarabici_DSM_4947
Bact_Deferribacteres_YP_003495334.1_metS_Deferribacter_desulfuricans_SSM1
Bact_Deferribacteres_YP_003505081.1_metS_Denitrovibrio_acetiphilus_DSM_12809
Deferribacteres
Bact_Chrysiogenetes_YP_004111863.1_metS_Desulfurispirillum_indicum_S5
Chrysiogenetes
Bact_Firmicutes_YP_699780.1_metS_Clostridium_perfringens_SM101
Bact_Firmicutes_YP_008912707.1_metS_Paenibacillus_polymyxa_CR1
Bact_Firmicutes_YP_006909230.1_metS_Dehalobacter_sp._DCA
Firmicutes
Bact_Fusobacteria_YP_008477006.1_metS_Fusobacterium_nucleatum_subsp._vincentii_3_1_36A2
Bact_Fusobacteria_YP_003308411.1_metS_Sebaldella_termitidis_ATCC_33386
Fusobacteria
Bact_Synergistetes_YP_003317710.1_metS_Thermanaerovibrio_acidaminovorans_DSM_6589
Bact_Synergistetes_YP_003553474.1_metS_Aminobacterium_colombiense_DSM_12261
Synergistetes
Bact_Thermus_YP_004057711.1_metS_Oceanithermus_profundus_DSM_14977
Bact_Thermus_YP_004202950.1_metS_Thermus_scotoductus_SA-01
Bact_Thermus_YP_002785570.1_metS_Deinococcus_deserti_VCD115
Thermus
Bact_Thermodesulfo_YP_004628202.1_metS_Thermodesulfobacterium_geofontis_OPF15
Bact_Thermodesulfo_YP_004626932.1_metS_Thermodesulfatator_indicus_DSM_15286
Thermodesulfobacteria
Bact_Chlamydiae_YP_003548054.1_metS_Coraliomargarita_akajimensis_DSM_45221
Bact_Chlamydiae_YP_001820527.1_metS_Opitutus_terrae_PB90-1
Bact_Chlamydiae_YP_001878693.1_metS_Akkermansia_muciniphila_ATCC_BAA-835
Chlamydiae
Bact_Acidobacteria_YP_005820274.1_metS_Fibrobacter_succinogenes_subsp._succinogenes_S85
Acidobacteria
Bact_Nitrospirae_YP_006767250.1_metS_Leptospirillum_ferriphilum_ML-04
Bact_Nitrospirae_YP_002248229.1_metS_Thermodesulfovibrio_yellowstonii_DSM_11347
Nitrospirae
Bact_Thermotogae_YP_001410306.1_metS_Fervidobacterium_nodosum_Rt17-B1
Bact_Thermotogae_YP_001306426.1_metS_Thermosipho_melanesiensis_BI429
Bact_Thermotogae_YP_002939750.1_metS_Kosmotoga_olearia_TBF_19.5.1
Thermotogae
Bact_Dictyoglomi_YP_002251787.1_metS_Dictyoglomus_thermophilum_H-6-12
Bact_Dictyoglomi_YP_002352196.1_metS_Dictyoglomus_turgidum_DSM_6724
Dictyoglomi
Bact_Tenericutes_YP_001620134.1_metS_Acholeplasma_laidlawii_PG-8A
Tenericutes
Bact_Caldiserica_YP_005473190.1_metS_Caldisericum_exile_AZM16c01
Caldiserica
Bact_Tenericutes_NP_950485.1_metS_Onion_yellows_phytoplasma_OY-M
Tenericutes
Bact_Beta_YP_004236200.1_metS_Acidovorax_avenae_subsp._avenae_ATCC_19860
Bact_Beta_YP_004128012.1_metS_Alicycliphilus_denitrificans_BC
Bact_Beta_YP_003981664.1_metS_Achromobacter_xylosoxidans_A8
β-protobacteria
Bact_Gamma_YP_001651403.1_metS_Actinobacillus_pleuropneumoniae_serovar_3_str._JL03
Bact_Gamma_YP_008043481.1_metS_Aeromonas_hydrophila_ML09-119
Bact_Gamma_YP_008176496.1_metS_Alteromonas_macleodii_str._Ionian_Sea_U8
γ-protobacteria
Bact_Planctomycetes_YP_004268955.1_metS_Planctomyces_brasiliensis_DSM_5305
Bact_Planctomycetes_YP_003629166.1_metS_Planctomyces_limnophilus_DSM_3776
Planctomycetes
Bact_Delta_YP_007022672.1_metS_Bdellovibrio_bacteriovorus_str._Tiberius
Bact_Delta_YP_002493045.1_metS_Anaeromyxobacter_dehalogenans_2CP-1
δ-protobacteria
Archae_Thaum_YP_006775853.1_metS_Candidatus_Nitrosopumilus_sp._AR2
Archae_Thaum_YP_001582297.1_metS_Nitrosopumilus_maritimus_SCM1
Archae_Thaum_YP_006863802.1_metS_Candidatus_Nitrososphaera_gargensis_Ga9.2
Thaumarchaeota
Bact_Chlorobi_YP_213853.1_metS_Bacteroides_fragilis_NCTC_9343
Bact_Chlorobi_YP_006867391.1_metS_Psychroflexus_torquis_ATCC_700755
Bact_Chlorobi_YP_005012952.1_metS_Niastella_koreensis_GR20-10
Bacteroidetes
Bact_Delta_YP_005034791.1_metS_Bacteriovorax_marinus_SJ
δ-protobacteria
Rhodo_005707242_metS_Galdieria_sulphuraria
Rhodo_005707241_metS_Galdieria_sulphuraria
Rhodo_544211748_metS_Cyanidioschyzon_merolae
Rhodo_005715120_metS_Chondrus_crispus
Rhodophyta
Plant_54290977_metS_Oryza_sativa
Plant_110289054_metS_Oryza_sativa
Plant_AT4G13780_metS_Arabidopsis_thaliana
Cyto. (SUBA3)
Green Plants
Algae_159487881_metS_Chlamydomonas_reinhardtii
Green Algae
Cilio_89307905_metS_Tetrahymena_thermophila
Cilio_145507618_metS_Paramecium_tetraurelia
Ciliophora
Mam_009179310.1_metS_Papio_anubis
Mam_001253877.1_metS_Macaca_mulatta
Mam_011918690.1_metS_Cercocebus_atys
Mam_012365764.1_metS_Nomascus_leucogenys
Mam_002823470.1_metS_Pongo_abelii
Mam_018893707.1_metS_Gorilla_gorilla
Mam_ENSG00000166986_metS_Homo_sapiens
Cyto. (GO) Mito. (GO) 6 Unknown
Mam_009423338.1_metS_Pan_troglodytes
Mammlia
Bact_Spirochaetes_YP_008902690.1_metS_Spirochaeta_sp._L21-RPul-D2
Bact_Spirochaetes_YP_005475184.1_metS_Spirochaeta_africana_DSM_8902
Bact_Spirochaetes_YP_004247222.1_metS_Sphaerochaeta_globosa_str._Buddy
Spirochaetes
Bact_Alpha_YP_004088522.1_metS_Asticcacaulis_excentricus_CB_48
α-protobacteria
Bact_Crenarchaeota_YP_007175526.1_metS_Caldisphaera_lagunensis_DSM_15908
Bact_Crenarchaeota_YP_003815674.1_metS_Acidilobus_saccharovorans_345-15
Archae_Kor_YP_001736509.1_metS_Candidatus_Korarchaeum_cryptofilum_OPF8
Bact_Crenarchaeota_YP_001539976.1_metS_Caldivirga_maquilingensis_IC-167
Archae_Nano_NP_963739.1_metS_Nanoarchaeum_equitans_Kin4-M
Archaea
Bact_Actino_YP_007870449.1_metS_Propionibacterium_avidum_44067
Actinobacteria
Bact_Chloroflexi_YP_002462330.1_metS_Chloroflexus_aggregans_DSM_9485
Bact_Chloroflexi_YP_004173042.1_metS_Anaerolinea_thermophila_UNI-1
Bact_Chloroflexi_YP_005440732.1_metS_Caldilinea_aerophila_DSM_14535_=_NBRC_104270
Chloroflexi
Bact_Firmicutes_YP_699779.1_metS_Clostridium_perfringens_SM101
Firmicutes
Archae_Eury_YP_003482468.1_metS_Aciduliprofundum_boonei_T469
Archae_Eury_YP_007246995.1_metS_Aciduliprofundum_sp._MAR08-339
Archaea
Archae_Eury_NP_142909.1_metS_Pyrococcus_horikoshii_OT3
0.4

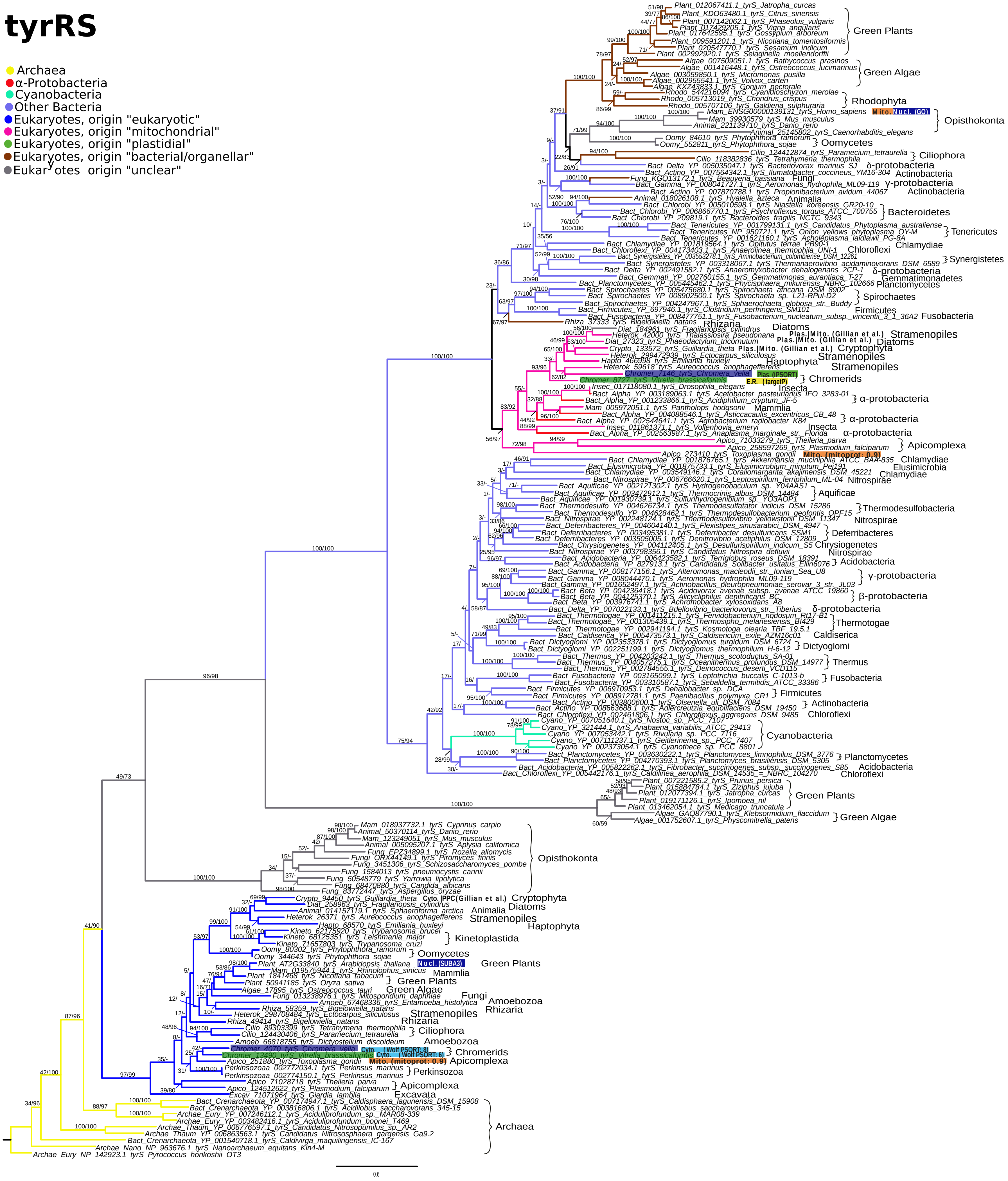
tyrRS
Archaea
α-Protobacteria
Cyanobacteria
Other Bacteria
Eukaryotes, origin "eukaryotic"
Eukaryotes, origin "mitochondrial"
Eukaryotes, origin "plastidial"
Eukaryotes, origin "bacterial/organellar"
Eukaryotes origin "unclear"
Plant_012067411.1_tyrS_Jatropha_curcas
Plant_KDO63480.1_tyrS_Citrus_sinensis
Plant_007142062.1_tyrS_Phaseolus_vulgaris
Plant_017429205.1_tyrS_Vigna_angularis
Plant_017642595.1_tyrS_Gossypium_arboreum
Plant_009591201.1_tyrS_Nicotiana_tomentosiformis
Plant_020547770.1_tyrS_Sesamum_indicum
Plant_002992920.1_tyrS_Selaginella_moellendorffii
Green Plants
Algae_007509051.1_tyrS_Bathycoccus_prasinos
Algae_001416448.1_tyrS_Ostreococcus_lucimarinus
Algae_003059850.1_tyrS_Micromonas_pusilla
Algae_002955541.1_tyrS_Volvox_carteri
Algae_KXZ43833.1_tyrS_Gonium_pectorale
Green Algae
Rhodo_544216094_tyrS_Cyanidioschyzon_merolae
Rhodo_005713019_tyrS_Chondrus_crispus
Rhodo_005707106_tyrS_Galdieria_sulphuraria
Rhodophyta
Mam_ENSG00000139131_tyrS_Homo_sapiens
Mito. Nucl. (GO)
Mam_39930579_tyrS_Mus_musculus
Animal_221139710_tyrS_Danio_rerio
Animal_25145802_tyrS_Caenorhabditis_elegans
Opisthokonta
Oomy_84610_tyrS_Phytophthora_ramorum
Oomy_552811_tyrS_Phytophthora_sojae
Oomycetes
Cilio_124412874_tyrS_Paramecium_tetraurelia
Cilio_118382836_tyrS_Tetrahymena_thermophila
Ciliophora
Bact_Delta_YP_005035047.1_tyrS_Bacteriovorax_marinus_SJ
δ-protobacteria
Bact_Actino_YP_007564342.1_tyrS_Ilumatobacter_coccineus_YM16-304
Actinobacteria
Fung_KGQ13172.1_tyrS_Beauveria_bassiana
Fungi
Bact_Gamma_YP_008041727.1_tyrS_Aeromonas_hydrophila_ML09-119
γ-protobacteria
Bact_Actino_YP_007870788.1_tyrS_Propionibacterium_avidum_44067
Actinobacteria
Animal_018026108.1_tyrS_Hyalella_azteca
Animalia
Bact_Chlorobi_YP_005010598.1_tyrS_Niastella_koreensis_GR20-10
Bact_Chlorobi_YP_006866770.1_tyrS_Psychroflexus_torquis_ATCC_700755
Bact_Chlorobi_YP_209819.1_tyrS_Bacteroides_fragilis_NCTC_9343
Bacteroidetes
Bact_Tenericutes_YP_001799131.1_tyrS_Candidatus_Phytoplasma_australiense
Bact_Tenericutes_NP_950721.1_tyrS_Onion_yellows_phytoplasma_OY-M
Bact_Tenericutes_YP_001621160.1_tyrS_Acholeplasma_laidlawii_PG-8A
Tenericutes
Bact_Chlamydiae_YP_001819564.1_tyrS_Opitutus_terrae_PB90-1
Chlamydiae
Bact_Chloroflexi_YP_004173403.1_tyrS_Anaerolinea_thermophila_UNI-1
Chloroflexi
Bact_Synergistetes_YP_003553278.1_tyrS_Aminobacterium_colombiense_DSM_12261
Bact_Synergistetes_YP_003318067.1_tyrS_Thermanaerovibrio_acidaminovorans_DSM_6589
Synergistetes
Bact_Delta_YP_002491582.1_tyrS_Anaeromyxobacter_dehalogenans_2CP-1
δ-protobacteria
Bact_Gemmati_YP_002760155.1_tyrS_Gemmatimonas_aurantiaca_T-27
Gemmatimonadetes
Bact_Planctomycetes_YP_005445462.1_tyrS_Phycisphaera_mikurensis_NBRC_102666
Planctomycetes
Bact_Spirochaetes_YP_005475680.1_tyrS_Spirochaeta_africana_DSM_8902
Bact_Spirochaetes_YP_008902500.1_tyrS_Spirochaeta_sp._L21-RPul-D2
Bact_Spirochaetes_YP_004247967.1_tyrS_Sphaerochaeta_globosa_str._Buddy
Spirochaetes
Bact_Firmicutes_YP_697946.1_tyrS_Clostridium_perfringens_SM101
Firmicutes
Bact_Fusobacteria_YP_008477751.1_tyrS_Fusobacterium_nucleatum_subsp._vincentii_3_1_36A2
Fusobacteria
Rhiza_37333_tyrS_Bigelowiella_natans
Rhizaria
Diat_184961_tyrS_Fragilariopsis_cylindrus
Diatoms
Heterok_42000_tyrS_Thalassiosira_pseudonana
Plas.|Mito. (Gillian et al.)
Stramenopiles
Diat_27323_tyrS_Phaeodactylum_tricornutum
Plas.|Mito. (Gillian et al.)
Diatoms
Crypto_133572_tyrS_Guillardia_theta
Plas.|Mito. (Gillian et al.)
Cryptophyta
Heterok_299472939_tyrS_Ectocarpus_siliculosus
Stramenopiles
Hapto_466998_tyrS_Emiliania_huxleyi
Haptophyta
Heterok_59618_tyrS_Aureococcus_anophagefferens
Stramenopiles
Chromer_7146_tyrS_Chromera_velia
Plas. (iPSORT)
Chromer_8727_tyrS_Vitrella_brassicaformis
E.R. (targetP)
Chromerids
Insec_017118080.1_tyrS_Drosophila_elegans
Insecta
Bact_Alpha_YP_003189063.1_tyrS_Acetobacter_pasteurianus_IFO_3283-01
Bact_Alpha_YP_001233866.1_tyrS_Acidiphilium_cryptum_JF-5
α-protobacteria
Mam_005972051.1_tyrS_Pantholops_hodgsonii
Mammlia
Bact_Alpha_YP_004088546.1_tyrS_Asticcacaulis_excentricus_CB_48
Bact_Alpha_YP_002544641.1_tyrS_Agrobacterium_radiobacter_K84
α-protobacteria
Insec_011861371.1_tyrS_Vollenhovia_emeryi
Insecta
Bact_Alpha_YP_002563987.1_tyrS_Anaplasma_marginale_str._Florida
α-protobacteria
Apico_71033279_tyrS_Theileria_parva
Apico_258597269_tyrS_Plasmodium_falciparum
Apicomplexa
Apico_273410_tyrS_Toxoplasma_gondii
Mito. (mitoprot: 0.9)
Bact_Chlamydiae_YP_001876765.1_tyrS_Akkermansia_muciniphila_ATCC_BAA-835
Chlamydiae
Bact_Elusimicrobia_YP_001875733.1_tyrS_Elusimicrobium_minutum_Pei191
Elusimicrobia
Bact_Chlamydiae_YP_003549146.1_tyrS_Coraliomargarita_akajimensis_DSM_45221
Chlamydiae
Bact_Nitrospirae_YP_006766620.1_tyrS_Leptospirillum_ferriphilum_ML-04
Nitrospirae
Bact_Aquificae_YP_002121302.1_tyrS_Hydrogenobaculum_sp._Y04AAS1
Bact_Aquificae_YP_003472912.1_tyrS_Thermocrinis_albus_DSM_14484
Bact_Aquificae_YP_001930739.1_tyrS_Sulfurihydrogenibium_sp._YO3AOP1
Aquificae
Bact_Thermodesulfo_YP_004626734.1_tyrS_Thermodesulfatator_indicus_DSM_15286
Bact_Thermodesulfo_YP_004628462.1_tyrS_Thermodesulfobacterium_geofontis_OPF15
Thermodesulfobacteria
Bact_Nitrospirae_YP_002248124.1_tyrS_Thermodesulfovibrio_yellowstonii_DSM_11347
Nitrospirae
Bact_Deferribacteres_YP_004604140.1_tyrS_Flexistipes_sinusarabici_DSM_4947
Bact_Deferribacteres_YP_003495381.1_tyrS_Deferribacter_desulfuricans_SSM1
Bact_Deferribacteres_YP_003505005.1_tyrS_Denitrovibrio_acetiphilus_DSM_12809
Deferribacteres
Bact_Chrysiogenetes_YP_004112405.1_tyrS_Desulfurispirillum_indicum_S5
Chrysiogenetes
Bact_Nitrospirae_YP_003798356.1_tyrS_Candidatus_Nitrospira_defluvii
Nitrospirae
Bact_Acidobacteria_YP_006423582.1_tyrS_Terriglobus_roseus_DSM_18391
Bact_Acidobacteria_YP_827913.1_tyrS_Candidatus_Solibacter_usitatus_Ellin6076
Acidobacteria
Bact_Gamma_YP_008177156.1_tyrS_Alteromonas_macleodii_str._Ionian_Sea_U8
Bact_Gamma_YP_008044470.1_tyrS_Aeromonas_hydrophila_ML09-119
Bact_Gamma_YP_001652497.1_tyrS_Actinobacillus_pleuropneumoniae_serovar_3_str._JL03
γ-protobacteria
Bact_Beta_YP_004236418.1_tyrS_Acidovorax_avenae_subsp._avenae_ATCC_19860
Bact_Beta_YP_004125370.1_tyrS_Alicycliphilus_denitrificans_BC
Bact_Beta_YP_003976741.1_tyrS_Achromobacter_xylosoxidans_A8
β-protobacteria
Bact_Delta_YP_007022133.1_tyrS_Bdellovibrio_bacteriovorus_str._Tiberius
δ-protobacteria
Bact_Thermotogae_YP_001411215.1_tyrS_Fervidobacterium_nodosum_Rt17-B1
Bact_Thermotogae_YP_001305439.1_tyrS_Thermosipho_melanesiensis_BI429
Bact_Thermotogae_YP_002941194.1_tyrS_Kosmotoga_olearia_TBF_19.5.1
Thermotogae
Bact_Caldiserica_YP_005473573.1_tyrS_Caldisericum_exile_AZM16c01
Caldiserica
Bact_Dictyoglomi_YP_002353378.1_tyrS_Dictyoglomus_turgidum_DSM_6724
Bact_Dictyoglomi_YP_002251199.1_tyrS_Dictyoglomus_thermophilum_H-6-12
Dictyoglomi
Bact_Thermus_YP_004203242.1_tyrS_Thermus_scotoductus_SA-01
Bact_Thermus_YP_004057275.1_tyrS_Oceanithermus_profundus_DSM_14977
Bact_Thermus_YP_002784555.1_tyrS_Deinococcus_deserti_VCD115
Thermus
Bact_Fusobacteria_YP_003165099.1_tyrS_Leptotrichia_buccalis_C-1013-b
Bact_Fusobacteria_YP_003310587.1_tyrS_Sebaldella_termitidis_ATCC_33386
Fusobacteria
Bact_Firmicutes_YP_006910953.1_tyrS_Dehalobacter_sp._DCA
Bact_Firmicutes_YP_008912781.1_tyrS_Paenibacillus_polymyxa_CR1
Firmicutes
Bact_Actino_YP_003800600.1_tyrS_Olsenella_uli_DSM_7084
Bact_Actino_YP_008663688.1_tyrS_Adlercreutzia_equolifaciens_DSM_19450
Actinobacteria
Bact_Chloroflexi_YP_002461806.1_tyrS_Chloroflexus_aggregans_DSM_9485
Chloroflexi
Cyano_YP_007051640.1_tyrS_Nostoc_sp._PCC_7107
Cyano_YP_321444.1_tyrS_Anabaena_variabilis_ATCC_29413
Cyano_YP_007053442.1_tyrS_Rivularia_sp._PCC_7116
Cyano_YP_007111237.1_tyrS_Geitlerinema_sp._PCC_7407
Cyano_YP_002373054.1_tyrS_Cyanothece_sp._PCC_8801
Cyanobacteria
Bact_Planctomycetes_YP_003630222.1_tyrS_Planctomyces_limnophilus_DSM_3776
Bact_Planctomycetes_YP_004270393.1_tyrS_Planctomyces_brasiliensis_DSM_5305
Planctomycetes
Bact_Acidobacteria_YP_005822262.1_tyrS_Fibrobacter_succinogenes_subsp._succinogenes_S85
Acidobacteria
Bact_Chloroflexi_YP_005442176.1_tyrS_Caldilinea_aerophila_DSM_14535_=_NBRC_104270
Chloroflexi
Plant_007221585.2_tyrS_Prunus_persica
Plant_015884784.1_tyrS_Ziziphus_jujuba
Plant_012077394.1_tyrS_Jatropha_curcas
Plant_019171126.1_tyrS_Ipomoea_nil
Plant_013462054.1_tyrS_Medicago_truncatula
Green Plants
Algae_GAQ87790.1_tyrS_Klebsormidium_flaccidum
Algae_001752607.1_tyrS_Physcomitrella_patens
Green Algae
Mam_018937732.1_tyrS_Cyprinus_carpio
Animal_50370114_tyrS_Danio_rerio
Mam_123249051_tyrS_Mus_musculus
Animal_005095207.1_tyrS_Aplysia_californica
Fung_EPZ34899.1_tyrS_Rozella_allomycis
Fungi_ORX44149.1_tyrS_Piromyces_finnis
Fung_3451306_tyrS_Schizosaccharomyces_pombe
Fung_1584013_tyrS_pneumocystis_carinii
Fung_50548779_tyrS_Yarrowia_lipolytica
Fung_68470880_tyrS_Candida_albicans
Fung_83772447_tyrS_Aspergillus_oryzae
Opisthokonta
Crypto_94450_tyrS_Guillardia_theta
Cyto.|PPC(Gillian et al.)
Cryptophyta
Diat_258963_tyrS_Fragilariopsis_cylindrus
Diatoms
Animal_014157119.1_tyrS_Sphaeroforma_arctica
Animalia
Heterok_26371_tyrS_Aureococcus_anophagefferens
Stramenopiles
Hapto_68570_tyrS_Emiliania_huxleyi
Haptophyta
Kineto_62175920_tyrS_Trypanosoma_brucei
Kineto_68125351_tyrS_Leishmania_major
Kineto_71657803_tyrS_Trypanosoma_cruzi
Kinetoplastida
Oomy_80302_tyrS_Phytophthora_ramorum
Oomy_344643_tyrS_Phytophthora_sojae
Oomycetes
Plant_AT2G33840_tyrS_Arabidopsis_thaliana
Nucl. (SUBA3)
Green Plants
Mam_019575944.1_tyrS_Rhinolophus_sinicus
Mammlia
Plant_1841468_tyrS_Nicotiana_tabacum
Plant_50941185_tyrS_Oryza_sativa
Green Plants
Algae_17895_tyrS_Ostreococcus_tauri
Green Algae
Fung_013238976.1_tyrS_Mitosporidium_daphniae
Fungi
Amoeb_67468336_tyrS_Entamoeba_histolytica
Amoebozoa
Rhiza_58359_tyrS_Bigelowiella_natans
Rhizaria
Heterok_298708484_tyrS_Ectocarpus_siliculosus
Stramenopiles
Rhiza_49414_tyrS_Bigelowiella_natans
Rhizaria
Cilio_89303399_tyrS_Tetrahymena_thermophila
Cilio_124430406_tyrS_Paramecium_tetraurelia
Ciliophora
Amoeb_66818755_tyrS_Dictyostelium_discoideum
Amoebozoa
Chromer_4070_tyrS_Chromera_velia
Cyto. (Wolf PSORT: 8)
Chromer_13490_tyrS_Vitrella_brassicaformis
Cyto. (Wolf PSORT: 6)
Chromerids
Apico_251880_tyrS_Toxoplasma_gondii
Mito. (mitoprot: 0.9)
Apicomplexa
Perkinsozoa_002772034.1_tyrS_Perkinsus_marinus
Perkinsozoa_002774150.1_tyrS_Perkinsus_marinus
Perkinsozoa
Apico_71028718_tyrS_Theileria_parva
Apico_124512622_tyrS_Plasmodium_falciparum
Apicomplexa
Excav_71071964_tyrS_Giardia_lamblia
Excavata
Bact_Crenarchaeota_YP_007174947.1_tyrS_Caldisphaera_lagunensis_DSM_15908
Bact_Crenarchaeota_YP_003816806.1_tyrS_Acidilobus_saccharovorans_345-15
Archae_Eury_YP_007246112.1_tyrS_Aciduliprofundum_sp._MAR08-339
Archae_Eury_YP_003482416.1_tyrS_Aciduliprofundum_boonei_T469
Archae_Thaum_YP_006776597.1_tyrS_Candidatus_Nitrosopumilus_sp._AR2
Archae_Thaum_YP_006863563.1_tyrS_Candidatus_Nitrososphaera_gargensis_Ga9.2
Bact_Crenarchaeota_YP_001540718.1_tyrS_Caldivirga_maquilingensis_IC-167
Archae_Nano_NP_963676.1_tyrS_Nanoarchaeum_equitans_Kin4-M
Archae_Eury_NP_142923.1_tyrS_Pyrococcus_horikoshii_OT3
Archaea
0.6

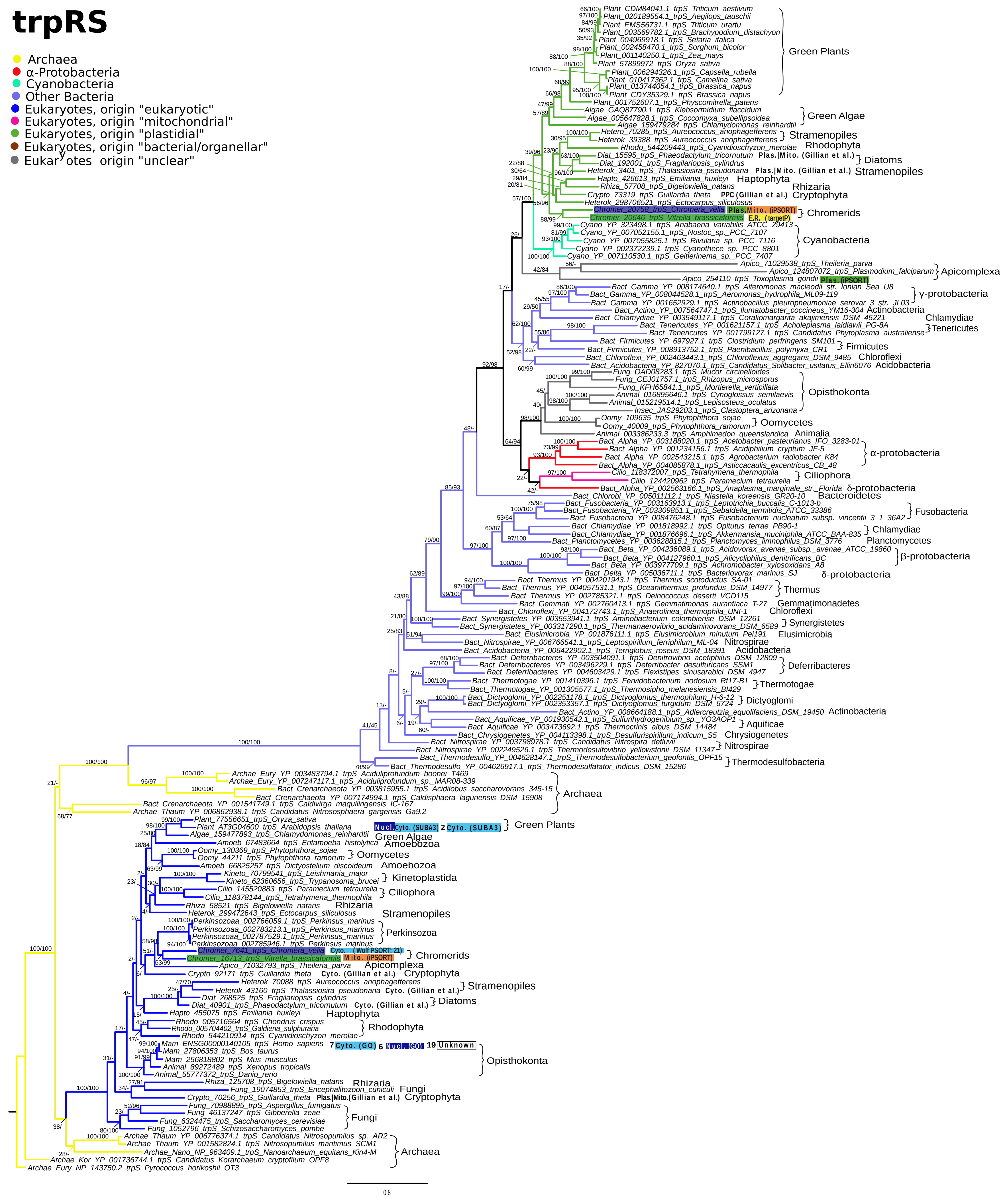

trpRS
Archaea
α-Protobacteria
Cyanobacteria
Other Bacteria
Eukaryotes, origin "eukaryotic"
Eukaryotes, origin "mitochondrial"
Eukaryotes, origin "plastidial"
Eukaryotes, origin "bacterial/organellar"
Eukaryotes origin "unclear"
Green Plants
Green Algae
Stramenopiles
Rhodophyta
Diatoms
Haptophyta
Rhizaria
Cryptophyta
Chromerids
Cyanobacteria
Apicomplexa
γ-protobacteria
Actinobacteria
Chlamydiae
Tenericutes
Firmicutes
Chloroflexi
Acidobacteria
Opisthokonta
Oomycetes
Animalia
α-protobacteria
Ciliophora
δ-protobacteria
Bacteroidetes
Fusobacteria
Planctomycetes
β-protobacteria
Thermus
Gemmatimonadetes
Synergistetes
Elusimicrobia
Nitrospirae
Deferribacteres
Thermotogae
Dictyoglomi
Aquificae
Chrysiogenetes
Thermodesulfobacteria
Amoebozoa
Kinetoplastida
Perkinsozoa
Fungi
0.8

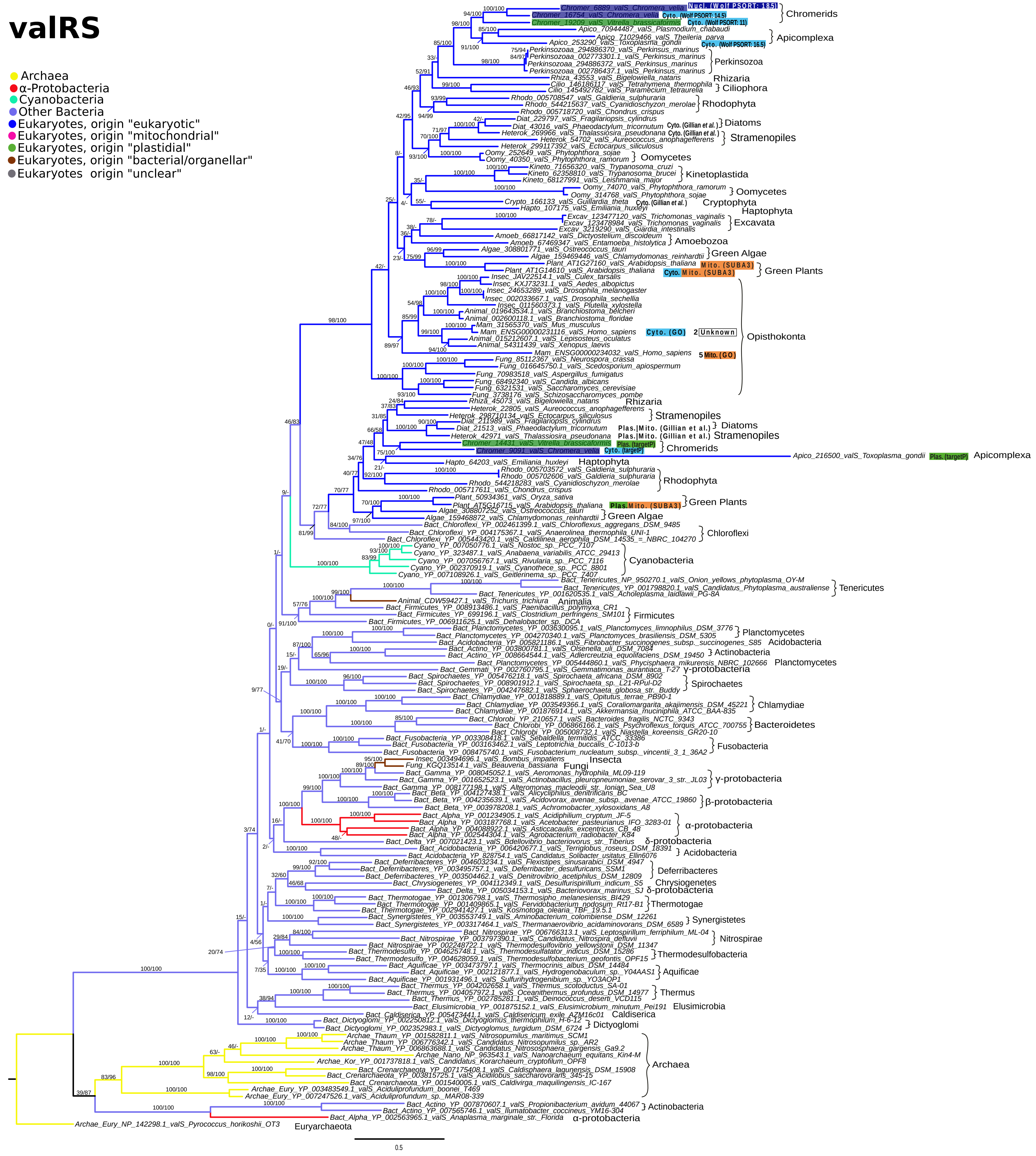
valRS
Archaea
α-Protobacteria
Cyanobacteria
Other Bacteria
Eukaryotes, origin "eukaryotic"
Eukaryotes, origin "mitochondrial"
Eukaryotes, origin "plastidial"
Eukaryotes, origin "bacterial/organellar"
Eukaryotes origin "unclear"
Chromer_6889_valS_Chromera_velia
Nucl. (Wolf PSORT: 18.5)
Chromer_16754_valS_Chromera_velia
Cyto. (Wolf PSORT: 14.5)
Chromer_19209_valS_Vitrella_brassicaformis
Cyto. (Wolf PSORT: 11)
Chromerids
Apico_70944487_valS_Plasmodium_chabaudi
Apico_71029466_valS_Theileria_parva
Apico_253290_valS_Toxoplasma_gondii
Cyto. (Wolf PSORT: 16.5)
Apicomplexa
Perkinsozoa_294886370_valS_Perkinsus_marinus
Perkinsozoa_002773301.1_valS_Perkinsus_marinus
Perkinsozoa_294886372_valS_Perkinsus_marinus
Perkinsozoa_002786437.1_valS_Perkinsus_marinus
Perkinsozoa
Rhiza_43553_valS_Bigelowiella_natans
Rhizaria
Cilio_146186117_valS_Tetrahymena_thermophila
Cilio_145492782_valS_Paramecium_tetraurelia
Ciliophora
Rhodo_005708547_valS_Galdieria_sulphuraria
Rhodo_544215637_valS_Cyanidioschyzon_merolae
Rhodo_005718720_valS_Chondrus_crispus
Rhodophyta
Diat_229797_valS_Fragilariopsis_cylindrus
Diat_43016_valS_Phaeodactylum_tricornutum
Cyto. (Gillian et al.)
Diatoms
Heterok_269966_valS_Thalassiosira_pseudonana
Cyto. (Gillian et al.)
Heterok_54702_valS_Aureococcus_anophagefferens
Heterok_299117392_valS_Ectocarpus_siliculosus
Stramenopiles
Oomy_252649_valS_Phytophthora_sojae
Oomy_40350_valS_Phytophthora_ramorum
Oomycetes
Kineto_71656320_valS_Trypanosoma_cruzi
Kineto_62358810_valS_Trypanosoma_brucei
Kineto_68127991_valS_Leishmania_major
Kinetoplastida
Oomy_74070_valS_Phytophthora_ramorum
Oomy_314768_valS_Phytophthora_sojae
Oomycetes
Crypto_166133_valS_Guillardia_theta
Cyto. (Gillian et al.)
Cryptophyta
Hapto_107175_valS_Emiliania_huxleyi
Haptophyta
Excav_123477120_valS_Trichomonas_vaginalis
Excav_123478984_valS_Trichomonas_vaginalis
Excav_3219290_valS_Giardia_intestinalis
Excavata
Amoeb_66817142_valS_Dictyostelium_discoideum
Amoeb_67469347_valS_Entamoeba_histolytica
Amoebozoa
Algae_308801771_valS_Ostreococcus_tauri
Algae_159469446_valS_Chlamydomonas_reinhardtii
Green Algae
Plant_AT1G27160_valS_Arabidopsis_thaliana
Mito. (SUBA3)
Plant_AT1G14610_valS_Arabidopsis_thaliana
Cyto. Mito. (SUBA3)
Green Plants
Insec_JAV22514.1_valS_Culex_tarsalis
Insec_KXJ73231.1_valS_Aedes_albopictus
Insec_24653289_valS_Drosophila_melanogaster
Insec_002033667.1_valS_Drosophila_sechellia
Insec_011560373.1_valS_Plutella_xylostella
Animal_019643534.1_valS_Branchiostoma_belcheri
Animal_002600118.1_valS_Branchiostoma_floridae
Mam_31565370_valS_Mus_musculus
Mam_ENSG00000231116_valS_Homo_sapiens
Cyto. (GO)
2 Unknown
Animal_015212607.1_valS_Lepisosteus_oculatus
Animal_54311439_valS_Xenopus_laevis
Mam_ENSG00000234032_valS_Homo_sapiens
5 Mito. (GO)
Opisthokonta
Fung_85112367_valS_Neurospora_crassa
Fung_016645750.1_valS_Scedosporium_apiospermum
Fung_70983518_valS_Aspergillus_fumigatus
Fung_68492340_valS_Candida_albicans
Fung_6321531_valS_Saccharomyces_cerevisiae
Fung_3738176_valS_Schizosaccharomyces_pombe
Rhiza_45073_valS_Bigelowiella_natans
Rhizaria
Heterok_22805_valS_Aureococcus_anophagefferens
Heterok_298710134_valS_Ectocarpus_siliculosus
Stramenopiles
Diat_211989_valS_Fragilariopsis_cylindrus
Diat_21513_valS_Phaeodactylum_tricornutum
Plas.|Mito. (Gillian et al.)
Diatoms
Heterok_42971_valS_Thalassiosira_pseudonana
Plas.|Mito. (Gillian et al.)
Stramenopiles
Chromer_14431_valS_Vitrella_brassicaformis
Plas. (targetP)
Chromer_9091_valS_Chromera_velia
Cyto. (targetP)
Chromerids
Apico_216500_valS_Toxoplasma_gondii
Plas. (targetP)
Apicomplexa
Hapto_64203_valS_Emiliania_huxleyi
Haptophyta
Rhodo_005703572_valS_Galdieria_sulphuraria
Rhodo_005702606_valS_Galdieria_sulphuraria
Rhodo_544218283_valS_Cyanidioschyzon_merolae
Rhodo_005717611_valS_Chondrus_crispus
Rhodophyta
Plant_50934361_valS_Oryza_sativa
Plant_AT5G16715_valS_Arabidopsis_thaliana
Plas. Mito. (SUBA3)
Green Plants
Algae_308807252_valS_Ostreococcus_tauri
Algae_159468872_valS_Chlamydomonas_reinhardtii
Green Algae
Bact_Chloroflexi_YP_002461399.1_valS_Chloroflexus_aggregans_DSM_9485
Bact_Chloroflexi_YP_004175367.1_valS_Anaerolinea_thermophila_UNI-1
Bact_Chloroflexi_YP_005443420.1_valS_Caldilinea_aerophila_DSM_14535_=_NBRC_104270
Chloroflexi
Cyano_YP_007050776.1_valS_Nostoc_sp._PCC_7107
Cyano_YP_323487.1_valS_Anabaena_variabilis_ATCC_29413
Cyano_YP_007056767.1_valS_Rivularia_sp._PCC_7116
Cyano_YP_002370919.1_valS_Cyanothece_sp._PCC_8801
Cyano_YP_007108926.1_valS_Geitlerinema_sp._PCC_7407
Cyanobacteria
Bact_Tenericutes_NP_950270.1_valS_Onion_yellows_phytoplasma_OY-M
Bact_Tenericutes_YP_001798820.1_valS_Candidatus_Phytoplasma_australiense
Bact_Tenericutes_YP_001620535.1_valS_Acholeplasma_laidlawii_PG-8A
Tenericutes
Animal_CDW59427.1_valS_Trichuris_trichiura
Animalia
Bact_Firmicutes_YP_008913486.1_valS_Paenibacillus_polymyxa_CR1
Bact_Firmicutes_YP_699196.1_valS_Clostridium_perfringens_SM101
Bact_Firmicutes_YP_006911625.1_valS_Dehalobacter_sp._DCA
Firmicutes
Bact_Planctomycetes_YP_003630095.1_valS_Planctomyces_limnophilus_DSM_3776
Bact_Planctomycetes_YP_004270340.1_valS_Planctomyces_brasiliensis_DSM_5305
Planctomycetes
Bact_Acidobacteria_YP_005821186.1_valS_Fibrobacter_succinogenes_subsp._succinogenes_S85
Acidobacteria
Bact_Actino_YP_003800781.1_valS_Olsenella_uli_DSM_7084
Bact_Actino_YP_008664544.1_valS_Adlercreutzia_equolifaciens_DSM_19450
Actinobacteria
Bact_Planctomycetes_YP_005444860.1_valS_Phycisphaera_mikurensis_NBRC_102666
Planctomycetes
Bact_Gemmati_YP_002760795.1_valS_Gemmatimonas_aurantiaca_T-27
γ-protobacteria
Bact_Spirochaetes_YP_005476218.1_valS_Treponema_africana_DSM_8902
Bact_Spirochaetes_YP_008901912.1_valS_Spirochaeta_sp._L21-RPul-D2
Bact_Spirochaetes_YP_004247682.1_valS_Sphaerochaeta_globosa_str._Buddy
Spirochaetes
Bact_Chlamydiae_YP_001818889.1_valS_Opitutus_terrae_PB90-1
Bact_Chlamydiae_YP_003549366.1_valS_Coraliomargarita_akajimensis_DSM_45221
Bact_Chlamydiae_YP_001876914.1_valS_Akkermansia_muciniphila_ATCC_BAA-835
Chlamydiae
Bact_Chlorobi_YP_210657.1_valS_Bacteroides_fragilis_NCTC_9343
Bact_Chlorobi_YP_006866166.1_valS_Psychroflexus_torquis_ATCC_700755
Bact_Chlorobi_YP_005008732.1_valS_Niastella_koreensis_GR20-10
Bacteroidetes
Bact_Fusobacteria_YP_003308418.1_valS_Sebaldella_termitidis_ATCC_33386
Bact_Fusobacteria_YP_003163462.1_valS_Leptotrichia_buccalis_C-1013-b
Bact_Fusobacteria_YP_008475740.1_valS_Fusobacterium_nucleatum_subsp._vincentii_3_1_36A2
Fusobacteria
Insec_003494696.1_valS_Bombus_impatiens
Insecta
Fung_KGQ13514.1_valS_Beauveria_bassiana
Fungi
Bact_Gamma_YP_008045052.1_valS_Aeromonas_hydrophila_ML09-119
Bact_Gamma_YP_001652523.1_valS_Actinobacillus_pleuropneumoniae_serovar_3_str._JL03
γ-protobacteria
Bact_Gamma_YP_008177198.1_valS_Alteromonas_macleodii_str._Ionian_Sea_U8
Bact_Beta_YP_004127438.1_valS_Alicycliphilus_denitrificans_BC
Bact_Beta_YP_004235639.1_valS_Acidovorax_avenae_subsp._avenae_ATCC_19860
Bact_Beta_YP_003978208.1_valS_Achromobacter_xylosoxidans_A8
β-protobacteria
Bact_Alpha_YP_001234905.1_valS_Acidiphilium_cryptum_JF-5
Bact_Alpha_YP_003187768.1_valS_Acetobacter_pasteurianus_IFO_3283-01
Bact_Alpha_YP_004088922.1_valS_Asticcacaulis_excentricus_CB_48
Bact_Alpha_YP_002544304.1_valS_Agrobacterium_radiobacter_K84
α-protobacteria
Bact_Delta_YP_007021423.1_valS_Bdellovibrio_bacteriovorus_str._Tiberius
δ-protobacteria
Bact_Acidobacteria_YP_006420677.1_valS_Terriglobus_roseus_DSM_18391
Bact_Acidobacteria_YP_828754.1_valS_Candidatus_Solibacter_usitatus_Ellin6076
Acidobacteria
Bact_Deferribacteres_YP_004603234.1_valS_Flexistipes_sinusarabici_DSM_4947
Bact_Deferribacteres_YP_003495757.1_valS_Deferribacter_desulfuricans_SSM1
Bact_Deferribacteres_YP_003504462.1_valS_Denitrovibrio_acetiphilus_DSM_12809
Deferribacteres
Bact_Chrysiogenetes_YP_004112349.1_valS_Desulfurispirillum_indicum_S5
Chrysiogenetes
Bact_Delta_YP_005034153.1_valS_Bacteriovorax_marinus_SJ
δ-protobacteria
Bact_Thermotogae_YP_001306798.1_valS_Thermosipho_melanesiensis_BI429
Bact_Thermotogae_YP_001409865.1_valS_Fervidobacterium_nodosum_Rt17-B1
Bact_Thermotogae_YP_002941427.1_valS_Kosmotoga_olearia_TBF_19.5.1
Thermotogae
Bact_Synergistetes_YP_003553749.1_valS_Aminobacterium_colombiense_DSM_12261
Bact_Synergistetes_YP_003317464.1_valS_Thermanaerovibrio_acidaminovorans_DSM_6589
Synergistetes
Bact_Nitrospirae_YP_006766313.1_valS_Leptospirillum_ferriphilum_ML-04
Bact_Nitrospirae_YP_003797390.1_valS_Candidatus_Nitrospira_defluvii
Nitrospirae
Bact_Nitrospirae_YP_002248722.1_valS_Thermodesulfovibrio_yellowstonii_DSM_11347
Bact_Thermodesulfo_YP_004625748.1_valS_Thermodesulfatator_indicus_DSM_15286
Bact_Thermodesulfo_YP_004628059.1_valS_Thermodesulfobacterium_geofontis_OPF15
Thermodesulfobacteria
Bact_Aquificae_YP_003473797.1_valS_Thermocrinis_albus_DSM_14484
Bact_Aquificae_YP_002121877.1_valS_Hydrogenobaculum_sp._Y04AAS1
Bact_Aquificae_YP_001931496.1_valS_Sulfurihydrogenibium_sp._YO3AOP1
Aquificae
Bact_Thermus_YP_004202658.1_valS_Thermus_scotoductus_SA-01
Bact_Thermus_YP_004057972.1_valS_Oceanithermus_profundus_DSM_14977
Bact_Thermus_YP_002785281.1_valS_Deinococcus_deserti_VCD115
Thermus
Bact_Elusimicrobia_YP_001875152.1_valS_Elusimicrobium_minutum_Pei191
Elusimicrobia
Bact_Caldiserica_YP_005473441.1_valS_Caldisericum_exile_AZM16c01
Caldiserica
Bact_Dictyoglomi_YP_002250812.1_valS_Dictyoglomus_thermophilum_H-6-12
Bact_Dictyoglomi_YP_002352983.1_valS_Dictyoglomus_turgidum_DSM_6724
Dictyoglomi
Archae_Thaum_YP_001582811.1_valS_Nitrosopumilus_maritimus_SCM1
Archae_Thaum_YP_006776342.1_valS_Candidatus_Nitrosopumilus_sp._AR2
Archae_Thaum_YP_006863688.1_valS_Candidatus_Nitrososphaera_gargensis_Ga9.2
Archae_Nano_NP_963543.1_valS_Nanoarchaeum_equitans_Kin4-M
Archae_Kor_YP_001737818.1_valS_Candidatus_Korarchaeum_cryptofilum_OPF8
Bact_Crenarchaeota_YP_007175408.1_valS_Caldisphaera_lagunensis_DSM_15908
Bact_Crenarchaeota_YP_003815725.1_valS_Acidilobus_saccharovorans_345-15
Bact_Crenarchaeota_YP_001540005.1_valS_Caldivirga_maquilingensis_IC-167
Archae_Eury_YP_003483549.1_valS_Aciduliprofundum_boonei_T469
Archae_Eury_YP_007247526.1_valS_Aciduliprofundum_sp._MAR08-339
Archaea
Bact_Actino_YP_007870607.1_valS_Propionibacterium_avidum_44067
Bact_Actino_YP_007565746.1_valS_Ilumatobacter_coccineus_YM16-304
Actinobacteria
Bact_Alpha_YP_002563965.1_valS_Anaplasma_marginale_str._Florida
α-protobacteria
Archae_Eury_NP_142298.1_valS_Pyrococcus_horikoshii_OT3
Euryarchaeota
0.5

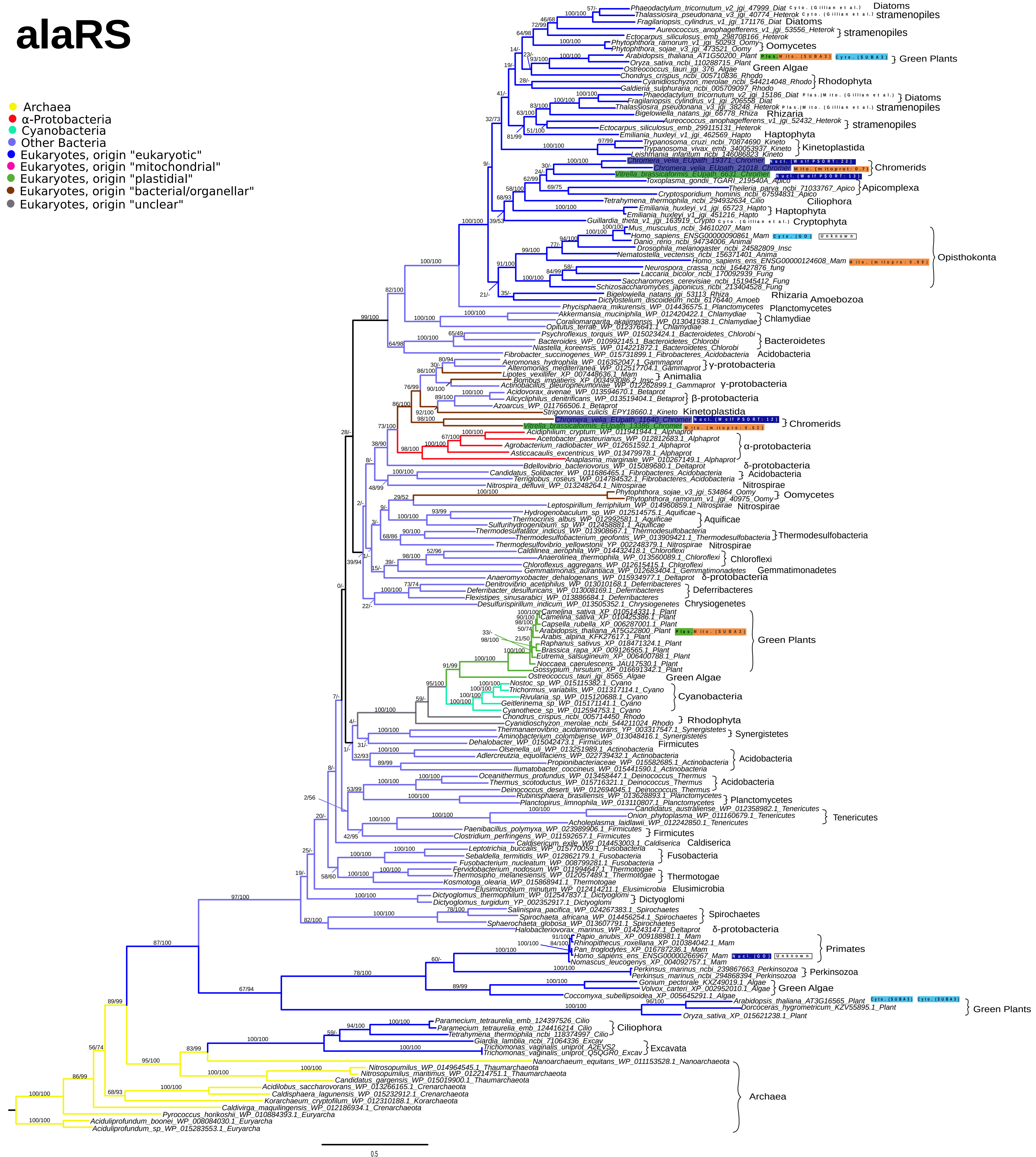

alaRS
Archaea
α-Protobacteria
Cyanobacteria
Other Bacteria
Eukaryotes, origin "eukaryotic"
Eukaryotes, origin "mitochondrial"
Eukaryotes, origin "plastidial"
Eukaryotes, origin "bacterial/organellar"
Eukaryotes, origin "unclear"
Phaeodactylum_tricornutum_v2_jgi_47999_Diat
Thalassiosira_pseudonana_v3_jgi_40774_Heterok
Fragilariopsis_cylindrus_v1_jgi_171176_Diat
Diatoms
stramenopiles
Aureococcus_anophagefferens_v1_jgi_53556_Heterok
Ectocarpus_siliculosus_emb_298708166_Heterok
Phytophthora_ramorum_v1_jgi_50293_Oomy
Phytophthora_sojae_v3_jgi_473521_Oomy
Oomycetes
Arabidopsis_thaliana_AT1G50200_Plant
Oryza_sativa_ncbi_110288715_Plant
Green Plants
Ostreococcus_tauri_jgi_376_Algae
Green Algae
Chondrus_crispus_ncbi_005710836_Rhodo
Cyanidioschyzon_merolae_ncbi_544214048_Rhodo
Galdieria_sulphuraria_ncbi_005709097_Rhodo
Rhodophyta
Phaeodactylum_tricornutum_v2_jgi_15186_Diat
Fragilariopsis_cylindrus_v1_jgi_206558_Diat
Thalassiosira_pseudonana_v3_jgi_38248_Heterok
Bigelowiella_natans_jgi_66778_Rhiza
Rhizaria
Aureococcus_anophagefferens_v1_jgi_52432_Heterok
Ectocarpus_siliculosus_emb_299115131_Heterok
Emiliania_huxleyi_v1_jgi_462569_Hapto
Haptophyta
Trypanosoma_cruzi_ncbi_70874690_Kineto
Trypanosoma_vivax_emb_340053937_Kineto
Leishmania_infantum_ncbi_146086823_Kineto
Kinetoplastida
Chromera_velia_EUpath_19371_Chromer
Chromera_velia_EUpath_21018_Chromer
Vitrella_brassicaformis_EUpath_6631_Chromer
Chromerids
Toxoplasma_gondii_TGARI_219540A_Apico
Theileria_parva_ncbi_71033767_Apico
Cryptosporidium_hominis_ncbi_67594831_Apico
Apicomplexa
Tetrahymena_thermophila_ncbi_294932634_Cilio
Ciliophora
Emiliania_huxleyi_v1_jgi_65723_Hapto
Emiliania_huxleyi_v1_jgi_451216_Hapto
Haptophyta
Guillardia_theta_v1_jgi_163919_Crypto
Cryptophyta
Mus_musculus_ncbi_34610207_Mam
Homo_sapiens_ENSG00000090861_Mam
Danio_rerio_ncbi_94734006_Animal
Drosophila_melanogaster_ncbi_24582809_Insc
Nematostella_vectensis_ncbi_156371401_Anima
Homo_sapiens_ens_ENSG00000124608_Mam
Neurospora_crassa_ncbi_164427876_fung
Laccaria_bicolor_ncbi_170092939_Fung
Saccharomyces_cerevisiae_ncbi_151945412_Fung
Schizosaccharomyces_japonicus_ncbi_213404528_Fung
Opisthokonta
Bigelowiella_natans_jgi_53113_Rhiza
Dictyostelium_discoideum_ncbi_6176440_Amoeb
Rhizaria
Amoebozoa
Phycisphaera_mikurensis_WP_014436575.1_Planctomycetes
Planctomycetes
Akkermansia_muciniphila_WP_012420422.1_Chlamydiae
Coraliomargarita_akajimensis_WP_013041938.1_Chlamydiae
Opitutus_terrae_WP_012376641.1_Chlamydiae
Chlamydiae
Psychroflexus_torquis_WP_015023424.1_Bacteroidetes_Chlorobi
Bacteroides_WP_010992145.1_Bacteroidetes_Chlorobi
Niastella_koreensis_WP_014221872.1_Bacteroidetes_Chlorobi
Bacteroidetes
Fibrobacter_succinogenes_WP_015731899.1_Fibrobacteres_Acidobacteria
Acidobacteria
Aeromonas_hydrophila_WP_016352047.1_Gammaprot
Alteromonas_mediterranea_WP_012517704.1_Gammaprot
γ-protobacteria
Lipotes_vexillifer_XP_007448636.1_Mam
Bombus_impatiens_XP_003493086.2_Insc
Animalia
Actinobacillus_pleuropneumoniae_WP_012262899.1_Gammaprot
γ-protobacteria
Acidovorax_avenae_WP_013594670.1_Betaprot
Alicycliphilus_denitrificans_WP_013519404.1_Betaprot
Azoarcus_WP_011766506.1_Betaprot
β-protobacteria
Strigomonas_culicis_EPY18660.1_Kineto
Kinetoplastida
Chromera_velia_EUpath_11640_Chromer
Vitrella_brassicaformis_EUpath_13386_Chromer
Chromerids
Acidiphilium_cryptum_WP_011941944.1_Alphaprot
Acetobacter_pasteurianus_WP_012812683.1_Alphaprot
Agrobacterium_radiobacter_WP_012651592.1_Alphaprot
Asticcacaulis_excentricus_WP_013479978.1_Alphaprot
Anaplasma_marginale_WP_010267149.1_Alphaprot
α-protobacteria
Bdellovibrio_bacteriovorus_WP_015089680.1_Deltaprot
δ-protobacteria
Candidatus_Solibacter_WP_011686465.1_Fibrobacteres_Acidobacteria
Terriglobus_roseus_WP_014784532.1_Fibrobacteres_Acidobacteria
Acidobacteria
Nitrospira_defluvii_WP_013248264.1_Nitrospirae
Nitrospirae
Phytophthora_sojae_v3_jgi_534864_Oomy
Phytophthora_ramorum_v1_jgi_40975_Oomy
Oomycetes
Leptospirillum_ferriphilum_WP_014960859.1_Nitrospirae
Nitrospirae
Hydrogenobaculum_sp_WP_012514575.1_Aquificae
Thermocrinis_albus_WP_012992581.1_Aquificae
Sulfurihydrogenibium_sp_WP_012458881.1_Aquificae
Aquificae
Thermodesulfatator_indicus_WP_013908667.1_Thermodesulfobacteria
Thermodesulfobacterium_geofontis_WP_013909421.1_Thermodesulfobacteria
Thermodesulfobacteria
Thermodesulfovibrio_yellowstonii_YP_002248379.1_Nitrospirae
Nitrospirae
Caldilinea_aerophila_WP_014432418.1_Chloroflexi
Anaerolinea_thermophila_WP_013560089.1_Chloroflexi
Chloroflexus_aggregans_WP_012615415.1_Chloroflexi
Chloroflexi
Gemmatimonas_aurantiaca_WP_012683404.1_Gemmatimonadetes
Gemmatimonadetes
Anaeromyxobacter_dehalogenans_WP_015934977.1_Deltaprot
δ-protobacteria
Denitrovibrio_acetiphilus_WP_013010168.1_Deferribacteres
Deferribacter_desulfuricans_WP_013008169.1_Deferribacteres
Flexistipes_sinusarabici_WP_013886684.1_Deferribacteres
Deferribacteres
Desulfurispirillum_indicum_WP_013505352.1_Chrysiogenetes
Chrysiogenetes
Camelina_sativa_XP_010514331.1_Plant
Camelina_sativa_XP_010425386.1_Plant
Capsella_rubella_XP_006287001.1_Plant
Arabidopsis_thaliana_AT5G22800_Plant
Arabis_alpina_KFK27617.1_Plant
Raphanus_sativus_XP_018471324.1_Plant
Brassica_rapa_XP_009126565.1_Plant
Eutrema_salsugineum_XP_006400788.1_Plant
Noccaea_caerulescens_JAU17530.1_Plant
Gossypium_hirsutum_XP_016691342.1_Plant
Green Plants
Ostreococcus_tauri_jgi_8565_Algae
Green Algae
Nostoc_sp_WP_015115382.1_Cyano
Trichormus_variabilis_WP_011317114.1_Cyano
Rivularia_sp_WP_015120688.1_Cyano
Geitlerinema_sp_WP_015171141.1_Cyano
Cyanothece_sp_WP_012594753.1_Cyano
Cyanobacteria
Chondrus_crispus_ncbi_005714450_Rhodo
Cyanidioschyzon_merolae_ncbi_544211024_Rhodo
Rhodophyta
Thermanaerovibrio_acidaminovorans_YP_003317547.1_Synergistetes
Aminobacterium_colombiense_WP_013048416.1_Synergistetes
Synergistetes
Dehalobacter_WP_015042473.1_Firmicutes
Firmicutes
Olsenella_uli_WP_013251989.1_Actinobacteria
Adlercreutzia_equolifaciens_WP_022739432.1_Actinobacteria
Propionibacteriaceae_WP_015582685.1_Actinobacteria
Ilumatobacter_coccineus_WP_015441590.1_Actinobacteria
Acidobacteria
Oceanithermus_profundus_WP_013458447.1_Deinococcus_Thermus
Thermus_scotoductus_WP_015716321.1_Deinococcus_Thermus
Deinococcus_deserti_WP_012694045.1_Deinococcus_Thermus
Acidobacteria
Rubinisphaera_brasiliensis_WP_013628893.1_Planctomycetes
Planctopirus_limnophila_WP_013110807.1_Planctomycetes
Planctomycetes
Candidatus_australiense_WP_012358982.1_Tenericutes
Onion_phytoplasma_WP_011160679.1_Tenericutes
Acholeplasma_laidlawii_WP_012242850.1_Tenericutes
Tenericutes
Paenibacillus_polymyxa_WP_023989906.1_Firmicutes
Clostridium_perfringens_WP_011592657.1_Firmicutes
Firmicutes
Caldisericum_exile_WP_014453003.1_Caldiserica
Caldiserica
Leptotrichia_buccalis_WP_015770059.1_Fusobacteria
Sebaldella_termitidis_WP_012862179.1_Fusobacteria
Fusobacterium_nucleatum_WP_008799281.1_Fusobacteria
Fusobacteria
Fervidobacterium_nodosum_WP_011994647.1_Thermotogae
Thermosipho_melanesiensis_WP_012057489.1_Thermotogae
Kosmotoga_olearia_WP_015868941.1_Thermotogae
Thermotogae
Elusimicrobium_minutum_WP_012414211.1_Elusimicrobia
Elusimicrobia
Dictyoglomus_thermophilum_WP_012547837.1_Dictyoglomi
Dictyoglomus_turgidum_YP_002352917.1_Dictyoglomi
Dictyoglomi
Salinispira_pacifica_WP_024267383.1_Spirochaetes
Spirochaeta_africana_WP_014456254.1_Spirochaetes
Sphaerochaeta_globosa_WP_013607791.1_Spirochaetes
Spirochaetes
Halobacteriovorax_marinus_WP_014243147.1_Deltaprot
δ-protobacteria
Papio_anubis_XP_009188981.1_Mam
Rhinopithecus_roxellana_XP_010384042.1_Mam
Pan_troglodytes_XP_016787236.1_Mam
Homo_sapiens_ens_ENSG00000266967_Mam
Nomascus_leucogenys_XP_004092757.1_Mam
Primates
Perkinsus_marinus_ncbi_239867663_Perkinsozoa
Perkinsus_marinus_ncbi_294868394_Perkinsozoa
Perkinsozoa
Gonium_pectorale_KXZ49019.1_Algae
Volvox_carteri_XP_002952010.1_Algae
Coccomyxa_subellipsoidea_XP_005645291.1_Algae
Green Algae
Arabidopsis_thaliana_AT3G16565_Plant
Dorcoceras_hygrometricum_KZV55895.1_Plant
Oryza_sativa_XP_015621238.1_Plant
Green Plants
Paramecium_tetraurelia_emb_124397526_Cilio
Paramecium_tetraurelia_emb_124416214_Cilio
Tetrahymena_thermophila_ncbi_118374997_Cilio
Ciliophora
Giardia_lamblia_ncbi_71064336_Excav
Trichomonas_vaginalis_uniprot_A2EVS2
Trichomonas_vaginalis_uniprot_Q5QGR0_Excav
Excavata
Nanoarchaeum_equitans_WP_011153528.1_Nanoarchaeota
Nitrosopumilus_WP_014964545.1_Thaumarchaeota
Nitrosopumilus_maritimus_WP_012214751.1_Thaumarchaeota
Candidatus_gargensis_WP_015019900.1_Thaumarchaeota
Acidilobus_saccharovorans_WP_013266165.1_Crenarchaeota
Caldisphaera_lagunensis_WP_015232912.1_Crenarchaeota
Korarchaeum_cryptofilum_WP_012310188.1_Korarchaeota
Caldivirga_maquilingensis_WP_012186934.1_Crenarchaeota
Pyrococcus_horikoshii_WP_010884393.1_Euryarcha
Aciduliprofundum_boonei_WP_008084030.1_Euryarcha
Aciduliprofundum_sp_WP_015283553.1_Euryarcha
Archaea
0.5

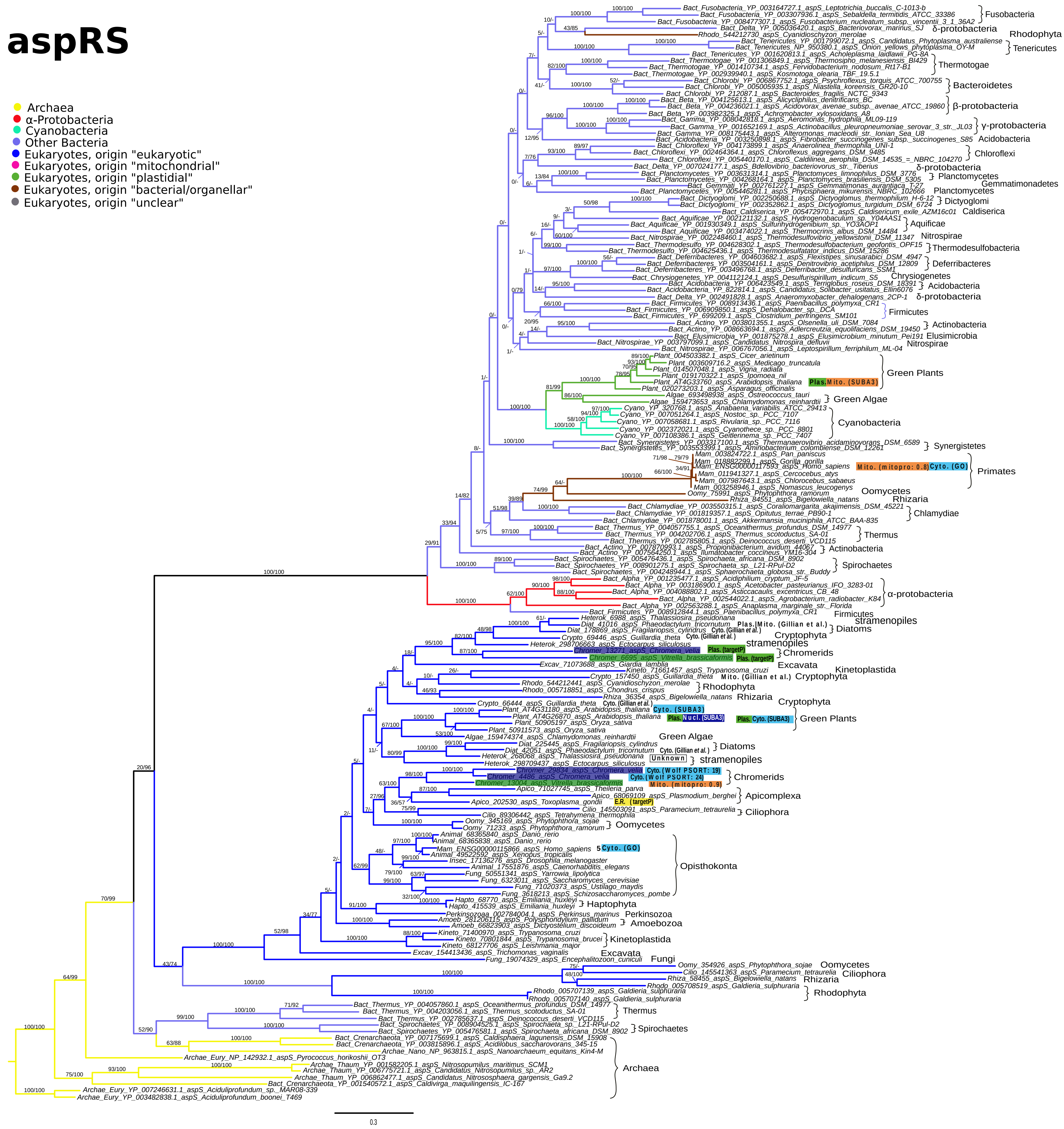
aspRS
Archaea
α-Protobacteria
Cyanobacteria
Other Bacteria
Eukaryotes, origin "eukaryotic"
Eukaryotes, origin "mitochondrial"
Eukaryotes, origin "plastidial"
Eukaryotes, origin "bacterial/organellar"
Eukaryotes, origin "unclear"
Bact_Fusobacteria_YP_003164727.1_aspS_Leptotrichia_buccalis_C-1013-b
Bact_Fusobacteria_YP_003307936.1_aspS_Sebaldella_termitidis_ATCC_33386
Bact_Fusobacteria_YP_008477307.1_aspS_Fusobacterium_nucleatum_subsp._vincentii_3_1_36A2
Fusobacteria
Bact_Delta_YP_005036420.1_aspS_Bacteriovorax_marinus_SJ
δ-protobacteria
Rhodo_544212730_aspS_Cyanidioschyzon_merolae
Rhodophyta
Bact_Tenericutes_YP_001799072.1_aspS_Candidatus_Phytoplasma_australiense
Bact_Tenericutes_NP_950380.1_aspS_Onion_yellows_phytoplasma_OY-M
Bact_Tenericutes_YP_001620813.1_aspS_Acholeplasma_laidlawii_PG-8A
Tenericutes
Bact_Thermotogae_YP_001306849.1_aspS_Thermosipho_melanesiensis_BI429
Bact_Thermotogae_YP_001410734.1_aspS_Fervidobacterium_nodosum_Rt17-B1
Bact_Thermotogae_YP_002939940.1_aspS_Kosmotoga_olearia_TBF_19.5.1
Thermotogae
Bact_Chlorobi_YP_006867752.1_aspS_Psychroflexus_torquis_ATCC_700755
Bact_Chlorobi_YP_005005935.1_aspS_Niastella_koreensis_GR20-10
Bact_Chlorobi_YP_212087.1_aspS_Bacteroides_fragilis_NCTC_9343
Bacteroidetes
Bact_Beta_YP_004125613.1_aspS_Alicycliphilus_denitrificans_BC
Bact_Beta_YP_004236021.1_aspS_Acidovorax_avenae_subsp._avenae_ATCC_19860
Bact_Beta_YP_003982325.1_aspS_Achromobacter_xylosoxidans_A8
β-protobacteria
Bact_Gamma_YP_008042818.1_aspS_Aeromonas_hydrophila_ML09-119
Bact_Gamma_YP_001652169.1_aspS_Actinobacillus_pleuropneumoniae_serovar_3_str._JL03
Bact_Gamma_YP_008175443.1_aspS_Alteromonas_macleodii_str._Ionian_Sea_U8
γ-protobacteria
Bact_Acidobacteria_YP_003250898.1_aspS_Fibrobacter_succinogenes_subsp._succinogenes_S85
Acidobacteria
Bact_Chloroflexi_YP_004173899.1_aspS_Anaerolinea_thermophila_UNI-1
Bact_Chloroflexi_YP_002464364.1_aspS_Chloroflexus_aggregans_DSM_9485
Bact_Chloroflexi_YP_005440170.1_aspS_Caldilinea_aerophila_DSM_14535_=_NBRC_104270
Chloroflexi
Bact_Delta_YP_007024177.1_aspS_Bdellovibrio_bacteriovorus_str._Tiberius
δ-protobacteria
Bact_Planctomycetes_YP_003631314.1_aspS_Planctomyces_limnophilus_DSM_3776
Bact_Planctomycetes_YP_004268164.1_aspS_Planctomyces_brasiliensis_DSM_5305
Planctomycetes
Bact_Gemmati_YP_002761227.1_aspS_Gemmatimonas_aurantiaca_T-27
Gemmatimonadetes
Bact_Planctomycetes_YP_005446281.1_aspS_Phycisphaera_mikurensis_NBRC_102666
Planctomycetes
Bact_Dictyoglomi_YP_002250688.1_aspS_Dictyoglomus_thermophilum_H-6-12
Bact_Dictyoglomi_YP_002352862.1_aspS_Dictyoglomus_turgidum_DSM_6724
Dictyoglomi
Bact_Caldiserica_YP_005472970.1_aspS_Caldisericum_exile_AZM16c01
Caldiserica
Bact_Aquificae_YP_002121132.1_aspS_Hydrogenobaculum_sp._Y04AAS1
Bact_Aquificae_YP_001930349.1_aspS_Sulfurihydrogenibium_sp._YO3AOP1
Bact_Aquificae_YP_003474022.1_aspS_Thermocrinis_albus_DSM_14484
Aquificae
Bact_Nitrospirae_YP_002248460.1_aspS_Thermodesulfovibrio_yellowstonii_DSM_11347
Nitrospirae
Bact_Thermodesulfo_YP_004628302.1_aspS_Thermodesulfobacterium_geofontis_OPF15
Bact_Thermodesulfo_YP_004625436.1_aspS_Thermodesulfatator_indicus_DSM_15286
Thermodesulfobacteria
Bact_Deferribacteres_YP_004603682.1_aspS_Flexistipes_sinusarabici_DSM_4947
Bact_Deferribacteres_YP_003504161.1_aspS_Denitrovibrio_acetiphilus_DSM_12809
Bact_Deferribacteres_YP_003496768.1_aspS_Deferribacter_desulfuricans_SSM1
Deferribacteres
Bact_Chrysiogenetes_YP_004112124.1_aspS_Desulfurispirillum_indicum_S5
Chrysiogenetes
Bact_Acidobacteria_YP_006423549.1_aspS_Terriglobus_roseus_DSM_18391
Bact_Acidobacteria_YP_822814.1_aspS_Candidatus_Solibacter_usitatus_Ellin6076
Acidobacteria
Bact_Delta_YP_002491828.1_aspS_Anaeromyxobacter_dehalogenans_2CP-1
δ-protobacteria
Bact_Firmicutes_YP_008913436.1_aspS_Paenibacillus_polymyxa_CR1
Bact_Firmicutes_YP_006909850.1_aspS_Dehalobacter_sp._DCA
Bact_Firmicutes_YP_699209.1_aspS_Clostridium_perfringens_SM101
Firmicutes
Bact_Actino_YP_003801355.1_aspS_Olsenella_uli_DSM_7084
Bact_Actino_YP_008663694.1_aspS_Adlercreutzia_equolifaciens_DSM_19450
Actinobacteria
Bact_Elusimicrobia_YP_001875278.1_aspS_Elusimicrobium_minutum_Pei191
Elusimicrobia
Bact_Nitrospirae_YP_003797099.1_aspS_Candidatus_Nitrospira_defluvii
Bact_Nitrospirae_YP_006767056.1_aspS_Leptospirillum_ferriphilum_ML-04
Nitrospirae
Plant_004503382.1_aspS_Cicer_arietinum
Plant_003609716.2_aspS_Medicago_truncatula
Plant_014507048.1_aspS_Vigna_radiata
Plant_019170322.1_aspS_Ipomoea_nil
Plant_AT4G33760_aspS_Arabidopsis_thaliana
Plas. Mito. (SUBA3)
Plant_020273203.1_aspS_Asparagus_officinalis
Green Plants
Algae_693498938_aspS_Ostreococcus_tauri
Algae_159473653_aspS_Chlamydomonas_reinhardtii
Green Algae
Cyano_YP_320768.1_aspS_Anabaena_variabilis_ATCC_29413
Cyano_YP_007051264.1_aspS_Nostoc_sp._PCC_7107
Cyano_YP_007058681.1_aspS_Rivularia_sp._PCC_7116
Cyano_YP_002372021.1_aspS_Cyanothece_sp._PCC_8801
Cyano_YP_007108386.1_aspS_Geitlerinema_sp._PCC_7407
Cyanobacteria
Bact_Synergistetes_YP_003317100.1_aspS_Thermanaerovibrio_acidaminovorans_DSM_6589
Bact_Synergistetes_YP_003553399.1_aspS_Aminobacterium_colombiense_DSM_12261
Synergistetes
Mam_003824722.1_aspS_Pan_paniscus
Mam_018882299.1_aspS_Gorilla_gorilla
Mam_ENSG00000117593_aspS_Homo_sapiens
Mito. (mitopro: 0.8) Cyto. (GO)
Mam_011941327.1_aspS_Cercocebus_atys
Mam_007987643.1_aspS_Chlorocebus_sabaeus
Mam_003258946.1_aspS_Nomascus_leucogenys
Primates
Oomy_75991_aspS_Phytophthora_ramorum
Oomycetes
Rhiza_84551_aspS_Bigelowiella_natans
Rhizaria
Bact_Chlamydiae_YP_003550315.1_aspS_Coraliomargarita_akajimensis_DSM_45221
Bact_Chlamydiae_YP_001819357.1_aspS_Opitutus_terrae_PB90-1
Bact_Chlamydiae_YP_001878001.1_aspS_Akkermansia_muciniphila_ATCC_BAA-835
Chlamydiae
Bact_Thermus_YP_004057755.1_aspS_Oceanithermus_profundus_DSM_14977
Bact_Thermus_YP_004202706.1_aspS_Thermus_scotoductus_SA-01
Bact_Thermus_YP_002785805.1_aspS_Deinococcus_deserti_VCD115
Thermus
Bact_Actino_YP_007870993.1_aspS_Propionibacterium_avidum_44067
Bact_Actino_YP_007564250.1_aspS_Ilumatobacter_coccineus_YM16-304
Actinobacteria
Bact_Spirochaetes_YP_005476436.1_aspS_Spirochaeta_africana_DSM_8902
Bact_Spirochaetes_YP_008901275.1_aspS_Spirochaeta_sp._L21-RPul-D2
Bact_Spirochaetes_YP_004248944.1_aspS_Sphaerochaeta_globosa_str._Buddy
Spirochaetes
Bact_Alpha_YP_001235477.1_aspS_Acidiphilium_cryptum_JF-5
Bact_Alpha_YP_003186900.1_aspS_Acetobacter_pasteurianus_IFO_3283-01
Bact_Alpha_YP_004088802.1_aspS_Asticcacaulis_excentricus_CB_48
Bact_Alpha_YP_002544022.1_aspS_Agrobacterium_radiobacter_K84
Bact_Alpha_YP_002563288.1_aspS_Anaplasma_marginale_str._Florida
α-protobacteria
Bact_Firmicutes_YP_008912844.1_aspS_Paenibacillus_polymyxa_CR1
Firmicutes
Heterok_6988_aspS_Thalassiosira_pseudonana
stramenopiles
Diat_41016_aspS_Phaeodactylum_tricornutum
Plas./Mito. (Gillian et al.)
Diat_178869_aspS_Fragilariopsis_cylindrus
Cyto. (Gillian et al.)
Diatoms
Crypto_69446_aspS_Guillardia_theta
Cyto. (Gillian et al.)
Cryptophyta
Heterok_298706663_aspS_Ectocarpus_siliculosus
stramenopiles
Chromer_13271_aspS_Chromera_velia
Plas. (targetP)
Chromer_6695_aspS_Vitrella_brassicaformis
Plas. (targetP)
Chromerids
Excav_71073688_aspS_Giardia_lamblia
Excavata
Kineto_71661457_aspS_Trypanosoma_cruzi
Kinetoplastida
Crypto_157450_aspS_Guillardia_theta
Mito. (Gillian et al.)
Cryptophyta
Rhodo_544212441_aspS_Cyanidioschyzon_merolae
Rhodo_005718851_aspS_Chondrus_crispus
Rhodophyta
Rhiza_36354_aspS_Bigelowiella_natans
Rhizaria
Crypto_66444_aspS_Guillardia_theta
Cyto. (Gillian et al.)
Cryptophyta
Plant_AT4G31180_aspS_Arabidopsis_thaliana
Cyto. (SUBA3)
Plant_AT4G26870_aspS_Arabidopsis_thaliana
Plas. Nucl. (SUBA3)
Plas. Cyto. (SUBA3)
Plant_50905197_aspS_Oryza_sativa
Plant_50911573_aspS_Oryza_sativa
Green Plants
Algae_159474374_aspS_Chlamydomonas_reinhardtii
Green Algae
Diat_225445_aspS_Fragilariopsis_cylindrus
Diat_42051_aspS_Phaeodactylum_tricornutum
Cyto. (Gillian et al.)
Diatoms
Heterok_268068_aspS_Thalassiosira_pseudonana
Unknown
Heterok_298709437_aspS_Ectocarpus_siliculosus
stramenopiles
Chromer_29834_aspS_Chromera_velia
Cyto. (Wolf PSORT: 19)
Chromer_4486_aspS_Chromera_velia
Cyto. (Wolf PSORT: 24)
Chromer_13004_aspS_Vitrella_brassicaformis
Mito. (mitopro: 0.9)
Chromerids
Apico_71027745_aspS_Theileria_parva
Apico_68069109_aspS_Plasmodium_berghei
Apico_202530_aspS_Toxoplasma_gondii
E.R. (targetP)
Apicomplexa
Cilio_145503091_aspS_Paramecium_tetraurelia
Cilio_89306442_aspS_Tetrahymena_thermophila
Ciliophora
Oomy_345169_aspS_Phytophthora_sojae
Oomy_71233_aspS_Phytophthora_ramorum
Oomycetes
Animal_68365840_aspS_Danio_rerio
Animal_68365838_aspS_Danio_rerio
Mam_ENSG00000115866_aspS_Homo_sapiens
5 Cyto. (GO)
Animal_49522592_aspS_Xenopus_tropicalis
Insec_17136276_aspS_Drosophila_melanogaster
Animal_17551876_aspS_Caenorhabditis_elegans
Fung_50551341_aspS_Yarrowia_lipolytica
Fung_6323011_aspS_Saccharomyces_cerevisiae
Fung_71020373_aspS_Ustilago_maydis
Fung_3618213_aspS_Schizosaccharomyces_pombe
Opisthokonta
Hapto_68770_aspS_Emiliania_huxleyi
Hapto_415539_aspS_Emiliania_huxleyi
Haptophyta
Perkinsozoa_002784004.1_aspS_Perkinsus_marinus
Perkinsozoa
Amoeb_281206115_aspS_Polysphondylium_pallidum
Amoeb_66823903_aspS_Dictyostelium_discoideum
Amoebozoa
Kineto_71400970_aspS_Trypanosoma_cruzi
Kineto_70801844_aspS_Trypanosoma_brucei
Kineto_68127706_aspS_Leishmania_major
Kinetoplastida
Excav_154413436_aspS_Trichomonas_vaginalis
Excavata
Fung_19074329_aspS_Encephalitozoon_cuniculi
Fungi
Oomy_354926_aspS_Phytophthora_sojae
Oomycetes
Cilio_145541363_aspS_Paramecium_tetraurelia
Ciliophora
Rhiza_58455_aspS_Bigelowiella_natans
Rhizaria
Rhodo_005708519_aspS_Galdieria_sulphuraria
Rhodo_005707139_aspS_Galdieria_sulphuraria
Rhodo_005707140_aspS_Galdieria_sulphuraria
Rhodophyta
Bact_Thermus_YP_004057860.1_aspS_Oceanithermus_profundus_DSM_14977
Bact_Thermus_YP_004203056.1_aspS_Thermus_scotoductus_SA-01
Bact_Thermus_YP_002785637.1_aspS_Deinococcus_deserti_VCD115
Thermus
Bact_Spirochaetes_YP_008904525.1_aspS_Spirochaeta_sp._L21-RPul-D2
Bact_Spirochaetes_YP_005476581.1_aspS_Spirochaeta_africana_DSM_8902
Spirochaetes
Bact_Crenarchaeota_YP_007175699.1_aspS_Caldisphaera_lagunensis_DSM_15908
Bact_Crenarchaeota_YP_003815896.1_aspS_Acidilobus_saccharovorans_345-15
Archae_Nano_NP_963815.1_aspS_Nanoarchaeum_equitans_Kin4-M
Archae_Eury_NP_142932.1_aspS_Pyrococcus_horikoshii_OT3
Archae_Thaum_YP_001582205.1_aspS_Nitrosopumilus_maritimus_SCM1
Archae_Thaum_YP_006775721.1_aspS_Candidatus_Nitrosopumilus_sp._AR2
Archae_Thaum_YP_006862477.1_aspS_Candidatus_Nitrososphaera_gargensis_Ga9.2
Bact_Crenarchaeota_YP_001540572.1_aspS_Caldivirga_maquilingensis_IC-167
Archae_Eury_YP_007246631.1_aspS_Aciduliprofundum_sp._MAR08-339
Archae_Eury_YP_003482838.1_aspS_Aciduliprofundum_boonei_T469
Archaea
0.3

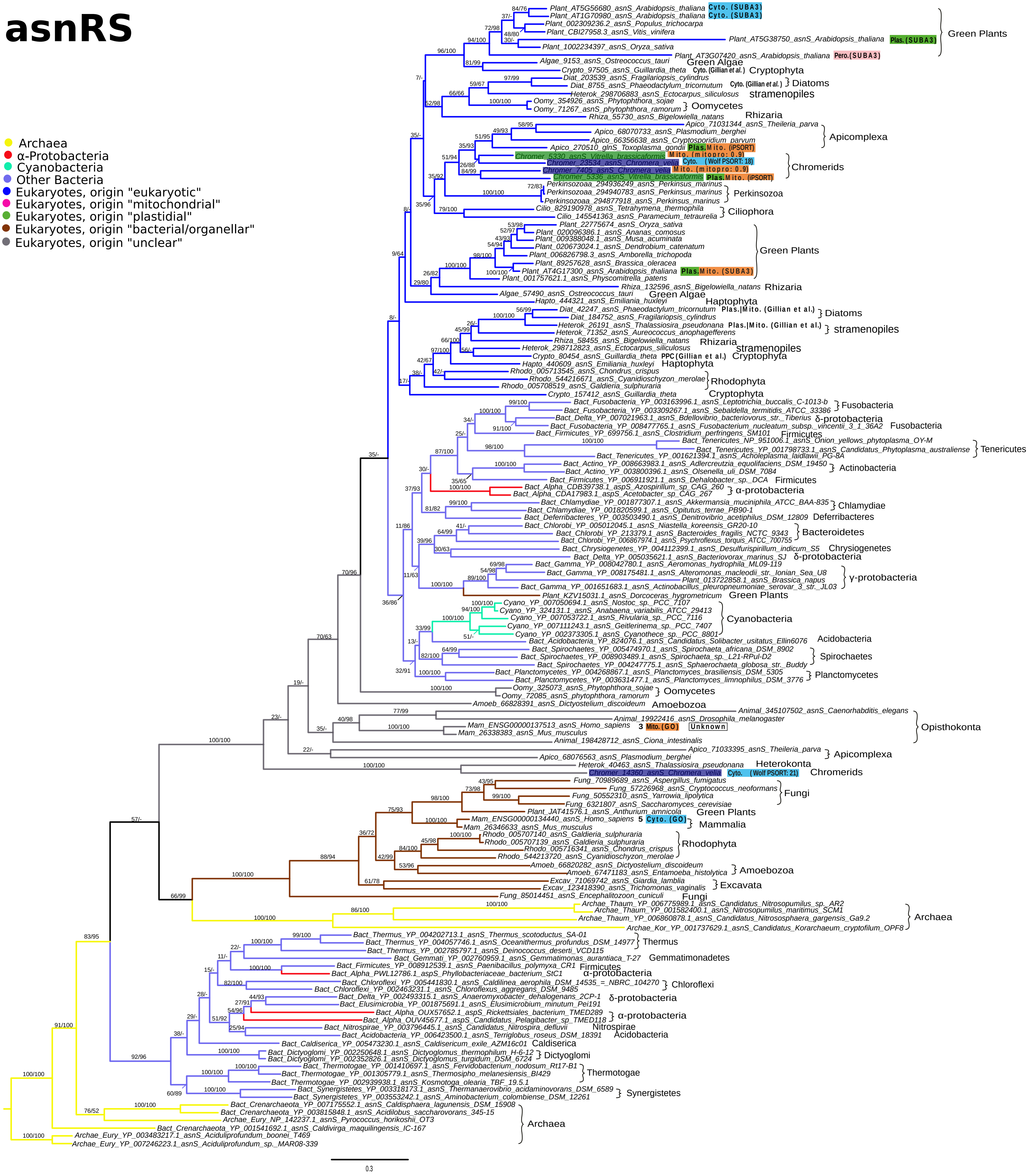

asnRS
Archaea
α-Protobacteria
Cyanobacteria
Other Bacteria
Eukaryotes, origin "eukaryotic"
Eukaryotes, origin "mitochondrial"
Eukaryotes, origin "plastidial"
Eukaryotes, origin "bacterial/organellar"
Eukaryotes, origin "unclear"
Green Plants
Green Algae
Cryptophyta
Diatoms
stramenopiles
Oomycetes
Rhizaria
Apicomplexa
Chromerids
Perkinsozoa
Ciliophora
Haptophyta
Rhodophyta
Fusobacteria
δ-protobacteria
Firmicutes
Tenericutes
Actinobacteria
α-protobacteria
Chlamydiae
Deferribacteres
Bacteroidetes
Chrysiogenetes
γ-protobacteria
Cyanobacteria
Acidobacteria
Spirochaetes
Planctomycetes
Amoebozoa
Opisthokonta
Heterokonta
Fungi
Mammalia
Excavata
Archaea
Thermus
Gemmatimonadetes
Chloroflexi
Nitrospirae
Caldiserica
Dictyoglomi
Thermotogae
Synergistetes
0.3

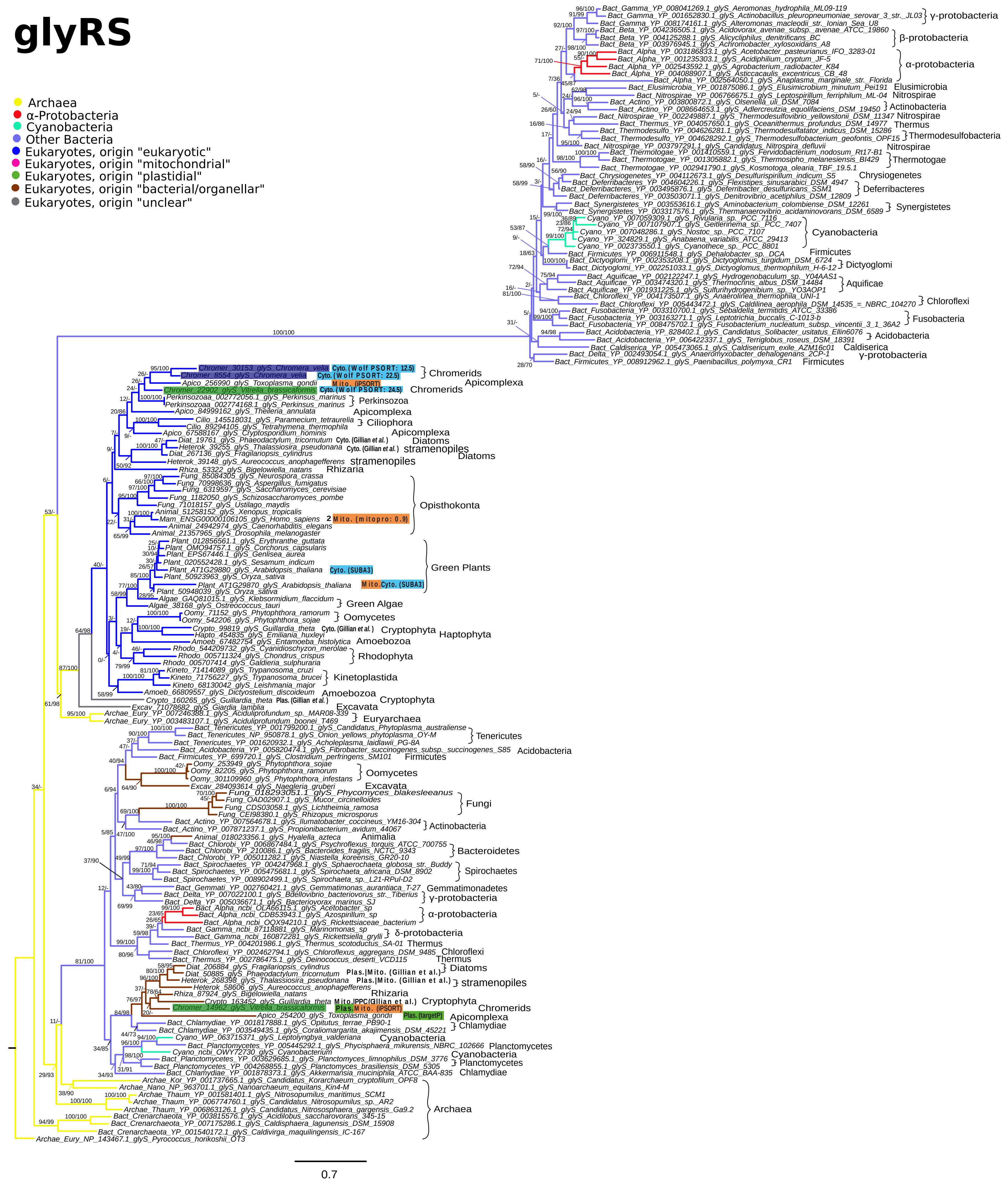

glyRS
Archaea
α-Protobacteria
Cyanobacteria
Other Bacteria
Eukaryotes, origin "eukaryotic"
Eukaryotes, origin "mitochondrial"
Eukaryotes, origin "plastidial"
Eukaryotes, origin "bacterial/organellar"
Eukaryotes, origin "unclear"
Bact_Gamma_YP_008041269.1_glyS_Aeromonas_hydrophila_ML09-119
Bact_Gamma_YP_001652830.1_glyS_Actinobacillus_pleuropneumoniae_serovar_3_str._JL03
Bact_Gamma_YP_008174161.1_glyS_Alteromonas_macleodii_str._Ionian_Sea_U8
γ-protobacteria
Bact_Beta_YP_004236505.1_glyS_Acidovorax_avenae_subsp._avenae_ATCC_19860
Bact_Beta_YP_004125288.1_glyS_Alicycliphilus_denitrificans_BC
Bact_Beta_YP_003976945.1_glyS_Achromobacter_xylosoxidans_A8
β-protobacteria
Bact_Alpha_YP_003186833.1_glyS_Acetobacter_pasteurianus_IFO_3283-01
Bact_Alpha_YP_001235303.1_glyS_Acidiphilium_cryptum_JF-5
Bact_Alpha_YP_002543592.1_glyS_Agrobacterium_radiobacter_K84
Bact_Alpha_YP_004088907.1_glyS_Asticcacaulis_excentricus_CB_48
Bact_Alpha_YP_002564050.1_glyS_Anaplasma_marginale_str._Florida
α-protobacteria
Bact_Elusimicrobia_YP_001875086.1_glyS_Elusimicrobium_minutum_Pei191
Elusimicrobia
Bact_Nitrospirae_YP_006766675.1_glyS_Leptospirillum_ferriphilum_ML-04
Nitrospirae
Bact_Actino_YP_003800872.1_glyS_Olsenella_uli_DSM_7084
Bact_Actino_YP_008664653.1_glyS_Adlercreutzia_equolifaciens_DSM_19450
Actinobacteria
Bact_Nitrospirae_YP_002249887.1_glyS_Thermodesulfovibrio_yellowstonii_DSM_11347
Nitrospirae
Bact_Thermus_YP_004057650.1_glyS_Oceanithermus_profundus_DSM_14977
Thermus
Bact_Thermodesulfo_YP_004626281.1_glyS_Thermodesulfatator_indicus_DSM_15286
Bact_Thermodesulfo_YP_004628292.1_glyS_Thermodesulfobacterium_geofontis_OPF15
Thermodesulfobacteria
Bact_Nitrospirae_YP_003797291.1_glyS_Candidatus_Nitrospira_defluvii
Nitrospirae
Bact_Thermotogae_YP_001410559.1_glyS_Fervidobacterium_nodosum_Rt17-B1
Bact_Thermotogae_YP_001305882.1_glyS_Thermosipho_melanesiensis_BI429
Bact_Thermotogae_YP_002941790.1_glyS_Kosmotoga_olearia_TBF_19.5.1
Thermotogae
Bact_Chrysiogenetes_YP_004112673.1_glyS_Desulfurispirillum_indicum_S5
Chrysiogenetes
Bact_Deferribacteres_YP_004604226.1_glyS_Flexistipes_sinusarabici_DSM_4947
Bact_Deferribacteres_YP_003495876.1_glyS_Deferribacter_desulfuricans_SSM1
Bact_Deferribacteres_YP_003503071.1_glyS_Denitrovibrio_acetiphilus_DSM_12809
Deferribacteres
Bact_Synergistetes_YP_003553616.1_glyS_Aminobacterium_colombiense_DSM_12261
Bact_Synergistetes_YP_003317576.1_glyS_Thermanaerovibrio_acidaminovorans_DSM_6589
Synergistetes
Cyano_YP_007059309.1_glyS_Rivularia_sp._PCC_7116
Cyano_YP_007107907.1_glyS_Geitlerinema_sp._PCC_7407
Cyano_YP_007048286.1_glyS_Nostoc_sp._PCC_7107
Cyano_YP_324829.1_glyS_Anabaena_variabilis_ATCC_29413
Cyano_YP_002373550.1_glyS_Cyanothece_sp._PCC_8801
Cyanobacteria
Bact_Firmicutes_YP_006911548.1_glyS_Dehalobacter_sp._DCA
Firmicutes
Bact_Dictyoglomi_YP_002353208.1_glyS_Dictyoglomus_turgidum_DSM_6724
Bact_Dictyoglomi_YP_002251033.1_glyS_Dictyoglomus_thermophilum_H-6-12
Dictyoglomi
Bact_Aquificae_YP_002122247.1_glyS_Hydrogenobaculum_sp._Y04AAS1
Bact_Aquificae_YP_003474320.1_glyS_Thermocrinis_albus_DSM_14484
Bact_Aquificae_YP_001931225.1_glyS_Sulfurihydrogenibium_sp._YO3AOP1
Aquificae
Bact_Chloroflexi_YP_004173507.1_glyS_Anaerolinea_thermophila_UNI-1
Bact_Chloroflexi_YP_005443472.1_glyS_Caldilinea_aerophila_DSM_14535_=_NBRC_104270
Chloroflexi
Bact_Fusobacteria_YP_003310700.1_glyS_Sebaldella_termitidis_ATCC_33386
Bact_Fusobacteria_YP_003163271.1_glyS_Leptotrichia_buccalis_C-1013-b
Bact_Fusobacteria_YP_008475702.1_glyS_Fusobacterium_nucleatum_subsp._vincentii_3_1_36A2
Fusobacteria
Bact_Acidobacteria_YP_828402.1_glyS_Candidatus_Solibacter_usitatus_Ellin6076
Bact_Acidobacteria_YP_006422337.1_glyS_Terriglobus_roseus_DSM_18391
Acidobacteria
Bact_Caldiserica_YP_005473065.1_glyS_Caldisericum_exile_AZM16c01
Caldiserica
Bact_Delta_YP_002493054.1_glyS_Anaeromyxobacter_dehalogenans_2CP-1
γ-protobacteria
Bact_Firmicutes_YP_008912962.1_glyS_Paenibacillus_polymyxa_CR1
Firmicutes
Chromer_30153_glyS_Chromera_velia
Cyto. (Wolf PSORT: 12.5)
Chromer_8554_glyS_Chromera_velia
Cyto. (Wolf PSORT: 22.5)
Chromerids
Apico_256990_glyS_Toxoplasma_gondii
Mito. (iPSORT)
Apicomplexa
Chromer_22902_glyS_Vitrella_brassicaformis
Cyto. (Wolf PSORT: 24.5)
Chromerids
Perkinsozoaa_002772056.1_glyS_Perkinsus_marinus
Perkinsozoaa_002774168.1_glyS_Perkinsus_marinus
Perkinsozoa
Apico_84999162_glyS_Theileria_annulata
Apicomplexa
Cilio_145518031_glyS_Paramecium_tetraurelia
Cilio_89294105_glyS_Tetrahymena_thermophila
Ciliophora
Apico_67588167_glyS_Cryptosporidium_hominis
Apicomplexa
Diat_19761_glyS_Phaeodactylum_tricornutum
Cyto. (Gillian et al.)
Heterok_39255_glyS_Thalassiosira_pseudonana
Cyto. (Gillian et al.)
Diat_267136_glyS_Fragilariopsis_cylindrus
Diatoms
stramenopiles
Diatoms
Heterok_39148_glyS_Aureococcus_anophagefferens
stramenopiles
Rhiza_53322_glyS_Bigelowiella_natans
Rhizaria
Fung_85084305_glyS_Neurospora_crassa
Fung_70998636_glyS_Aspergillus_fumigatus
Fung_6319597_glyS_Saccharomyces_cerevisiae
Fung_1182050_glyS_Schizosaccharomyces_pombe
Fung_71018157_glyS_Ustilago_maydis
Animal_51258152_glyS_Xenopus_tropicalis
Mam_ENSG00000106105_glyS_Homo_sapiens
2 Mito. (mitopro: 0.9)
Animal_24942974_glyS_Caenorhabditis_elegans
Animal_21357965_glyS_Drosophila_melanogaster
Opisthokonta
Plant_012856561.1_glyS_Erythranthe_guttata
Plant_OMO94757.1_glyS_Corchorus_capsularis
Plant_EPS67446.1_glyS_Genlisea_aurea
Plant_020552428.1_glyS_Sesamum_indicum
Plant_AT1G29880_glyS_Arabidopsis_thaliana
Cyto. (SUBA3)
Plant_50923963_glyS_Oryza_sativa
Plant_AT1G29870_glyS_Arabidopsis_thaliana
Mito.Cyto. (SUBA3)
Plant_50948039_glyS_Oryza_sativa
Green Plants
Algae_GAQ81015.1_glyS_Klebsormidium_flaccidum
Algae_38168_glyS_Ostreococcus_tauri
Green Algae
Oomy_71152_glyS_Phytophthora_ramorum
Oomy_542206_glyS_Phytophthora_sojae
Oomycetes
Crypto_99819_glyS_Guillardia_theta
Cyto. (Gillian et al.)
Cryptophyta
Hapto_454835_glyS_Emiliania_huxleyi
Haptophyta
Amoeb_67482754_glyS_Entamoeba_histolytica
Amoebozoa
Rhodo_544209732_glyS_Cyanidioschyzon_merolae
Rhodo_005711324_glyS_Chondrus_crispus
Rhodo_005707414_glyS_Galdieria_sulphuraria
Rhodophyta
Kineto_71414089_glyS_Trypanosoma_cruzi
Kineto_71756227_glyS_Trypanosoma_brucei
Kineto_68130042_glyS_Leishmania_major
Kinetoplastida
Amoeb_66809557_glyS_Dictyostelium_discoideum
Amoebozoa
Crypto_160265_glyS_Guillardia_theta
Plas. (Gillian et al.)
Cryptophyta
Excav_71078682_glyS_Giardia_lamblia
Excavata
Archae_Eury_YP_007246388.1_glyS_Aciduliprofundum_sp._MAR08-339
Archae_Eury_YP_003483107.1_glyS_Aciduliprofundum_boonei_T469
Euryarchaea
Bact_Tenericutes_YP_001799200.1_glyS_Candidatus_Phytoplasma_australiense
Bact_Tenericutes_NP_950878.1_glyS_Onion_yellows_phytoplasma_OY-M
Bact_Tenericutes_YP_001620932.1_glyS_Acholeplasma_laidlawii_PG-8A
Tenericutes
Bact_Acidobacteria_YP_005820474.1_glyS_Fibrobacter_succinogenes_subsp._succinogenes_S85
Acidobacteria
Bact_Firmicutes_YP_699720.1_glyS_Clostridium_perfringens_SM101
Firmicutes
Oomy_253949_glyS_Phytophthora_sojae
Oomy_82205_glyS_Phytophthora_ramorum
Oomy_301109960_glyS_Phytophthora_infestans
Oomycetes
Excav_284093614_glyS_Naegleria_gruberi
Excavata
Fung_018293051.1_glyS_Phycomyces_blakesleeanus
Fung_OAD02907.1_glyS_Mucor_circinelloides
Fung_CDS03058.1_glyS_Lichtheimia_ramosa
Fung_CEI98380.1_glyS_Rhizopus_microsporus
Fungi
Bact_Actino_YP_007564678.1_glyS_Ilumatobacter_coccineus_YM16-304
Bact_Actino_YP_007871237.1_glyS_Propionibacterium_avidum_44067
Actinobacteria
Animal_018023356.1_glyS_Hyalella_azteca
Animalia
Bact_Chlorobi_YP_006867484.1_glyS_Psychroflexus_torquis_ATCC_700755
Bact_Chlorobi_YP_210086.1_glyS_Bacteroides_fragilis_NCTC_9343
Bact_Chlorobi_YP_005011282.1_glyS_Niastella_koreensis_GR20-10
Bacteroidetes
Bact_Spirochaetes_YP_004247968.1_glyS_Sphaerochaeta_globosa_str._Buddy
Bact_Spirochaetes_YP_005475681.1_glyS_Spirochaeta_africana_DSM_8902
Bact_Spirochaetes_YP_008902499.1_glyS_Spirochaeta_sp._L21-RPul-D2
Spirochaetes
Bact_Gemmati_YP_002760421.1_glyS_Gemmatimonas_aurantiaca_T-27
Gemmatimonadetes
Bact_Delta_YP_007022100.1_glyS_Bdellovibrio_bacteriovorus_str._Tiberius
Bact_Delta_YP_005036671.1_glyS_Bacteriovorax_marinus_SJ
γ-protobacteria
Bact_Alpha_ncbi_OLA66115.1_glyS_Acetobacter_sp
Bact_Alpha_ncbi_CDB53943.1_glyS_Azospirillum_sp
Bact_Alpha_ncbi_OQX94210.1_glyS_Rickettsiaceae_bacterium
α-protobacteria
Bact_Gamma_ncbi_87118881_glyS_Marinomonas_sp
Bact_Gamma_ncbi_160872281_glyS_Rickettsiella_grylli
δ-protobacteria
Bact_Thermus_YP_004201986.1_glyS_Thermus_scotoductus_SA-01
Thermus
Bact_Chloroflexi_YP_002462794.1_glyS_Chloroflexus_aggregans_DSM_9485
Chloroflexi
Bact_Thermus_YP_002786475.1_glyS_Deinococcus_deserti_VCD115
Thermus
Diat_206884_glyS_Fragilariopsis_cylindrus
Diat_50885_glyS_Phaeodactylum_tricornutum
Plas.|Mito. (Gillian et al.)
Diatoms
Heterok_268398_glyS_Thalassiosira_pseudonana
Plas.|Mito. (Gillian et al.)
Heterok_58606_glyS_Aureococcus_anophagefferens
stramenopiles
Rhiza_87924_glyS_Bigelowiella_natans
Rhizaria
Crypto_163452_glyS_Guillardia_theta
Mito.|PPC (Gillian et al.)
Cryptophyta
Chromer_14962_glyS_Vitrella_brassicaformis
Plas.|Mito. (iPSORT)
Chromerids
Apico_254200_glyS_Toxoplasma_gondii
Plas. (targetP)
Apicomplexa
Bact_Chlamydiae_YP_001817888.1_glyS_Opitutus_terrae_PB90-1
Bact_Chlamydiae_YP_003549435.1_glyS_Coraliomargarita_akajimensis_DSM_45221
Chlamydiae
Cyano_WP_063715371_glyS_Leptolyngbya_valderiana
Cyanobacteria
Bact_Planctomycetes_YP_005445292.1_glyS_Phycisphaera_mikurensis_NBRC_102666
Planctomycetes
Cyano_ncbi_OWY72730_glyS_Cyanobacterium
Cyanobacteria
Bact_Planctomycetes_YP_003629685.1_glyS_Planctomyces_limnophilus_DSM_3776
Bact_Planctomycetes_YP_004268855.1_glyS_Planctomyces_brasiliensis_DSM_5305
Planctomycetes
Bact_Chlamydiae_YP_001878373.1_glyS_Akkermansia_muciniphila_ATCC_BAA-835
Chlamydiae
Archae_Kor_YP_001737665.1_glyS_Candidatus_Korarchaeum_cryptofilum_OPF8
Archae_Nano_NP_963701.1_glyS_Nanoarchaeum_equitans_Kin4-M
Archae_Thaum_YP_001581401.1_glyS_Nitrosopumilus_maritimus_SCM1
Archae_Thaum_YP_006774760.1_glyS_Candidatus_Nitrosopumilus_sp._AR2
Archae_Thaum_YP_006863126.1_glyS_Candidatus_Nitrososphaera_gargensis_Ga9.2
Bact_Crenarchaeota_YP_003815576.1_glyS_Acidilobus_saccharovorans_345-15
Bact_Crenarchaeota_YP_007175286.1_glyS_Caldisphaera_lagunensis_DSM_15908
Bact_Crenarchaeota_YP_001540172.1_glyS_Caldivirga_maquilingensis_IC-167
Archaea
Archae_Eury_NP_143467.1_glyS_Pyrococcus_horikoshii_OT3
0.7

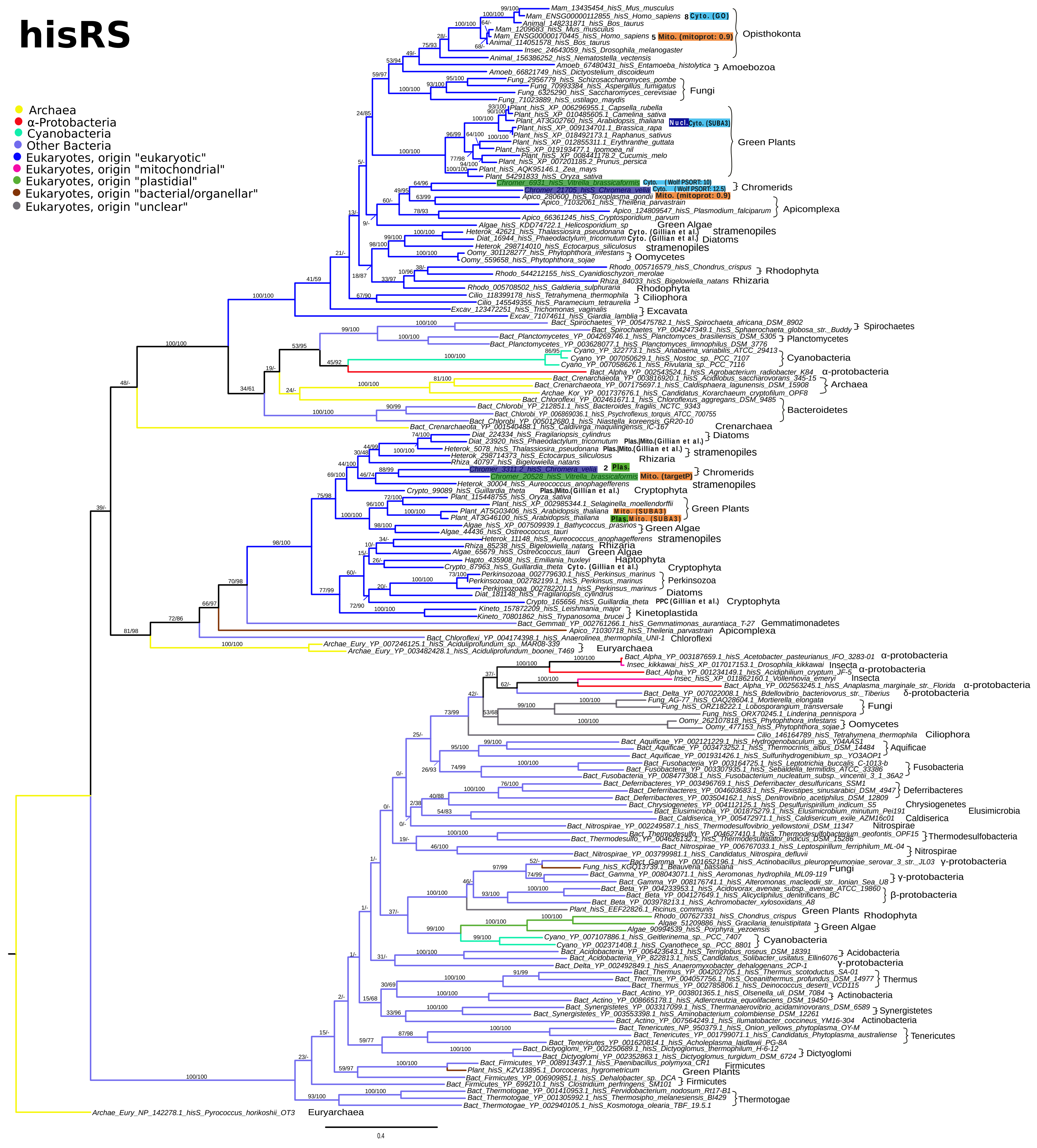
hisRS
Archaea
α-Protobacteria
Cyanobacteria
Other Bacteria
Eukaryotes, origin "eukaryotic"
Eukaryotes, origin "mitochondrial"
Eukaryotes, origin "plastidial"
Eukaryotes, origin "bacterial/organellar"
Eukaryotes, origin "unclear"
Opisthokonta
Amoebozoa
Fungi
Green Plants
Chromerids
Apicomplexa
Green Algae
stramenopiles
Diatoms
Oomycetes
Rhodophyta
Rhizaria
Ciliophora
Excavata
Spirochaetes
Planctomycetes
Cyanobacteria
α-protobacteria
Archaea
Bacteroidetes
Crenarchaea
Cryptophyta
Haptophyta
Perkinsozoa
Kinetoplastida
Gemmatimonadetes
Chloroflexi
Euryarchaea
Insecta
δ-protobacteria
Aquificae
Fusobacteria
Deferribacteres
Chrysiogenetes
Elusimicrobia
Caldiserica
Nitrospirae
Thermodesulfobacteria
γ-protobacteria
β-protobacteria
Acidobacteria
Thermus
Actinobacteria
Synergistetes
Tenericutes
Dictyoglomi
Firmicutes
Thermotogae
0.4

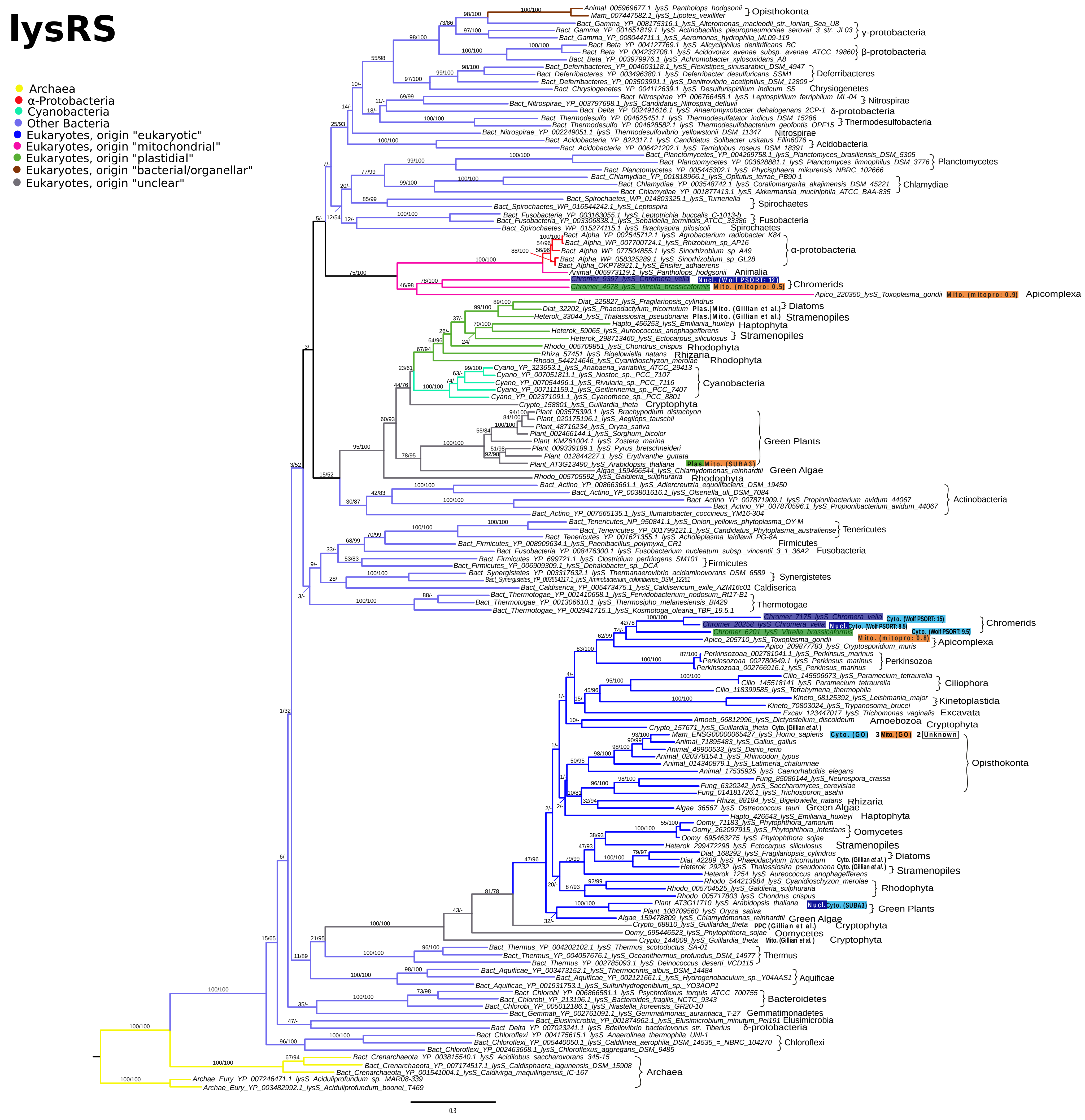
lysRS
Archaea
α-Protobacteria
Cyanobacteria
Other Bacteria
Eukaryotes, origin "eukaryotic"
Eukaryotes, origin "mitochondrial"
Eukaryotes, origin "plastidial"
Eukaryotes, origin "bacterial/organellar"
Eukaryotes, origin "unclear"
Animal_005969677.1_lysS_Pantholops_hodgsonii
Mam_007447582.1_lysS_Lipotes_vexillifer
Opisthokonta
Bact_Gamma_YP_008175316.1_lysS_Alteromonas_macleodii_str._Ionian_Sea_U8
Bact_Gamma_YP_001651819.1_lysS_Actinobacillus_pleuropneumoniae_serovar_3_str._JL03
Bact_Gamma_YP_008044711.1_lysS_Aeromonas_hydrophila_ML09-119
γ-protobacteria
Bact_Beta_YP_004127769.1_lysS_Alicycliphilus_denitrificans_BC
Bact_Beta_YP_004233708.1_lysS_Acidovorax_avenae_subsp._avenae_ATCC_19860
Bact_Beta_YP_003979976.1_lysS_Achromobacter_xylosoxidans_A8
β-protobacteria
Bact_Deferribacteres_YP_004603118.1_lysS_Flexistipes_sinusarabici_DSM_4947
Bact_Deferribacteres_YP_003496380.1_lysS_Deferribacter_desulfuricans_SSM1
Bact_Deferribacteres_YP_003503991.1_lysS_Denitrovibrio_acetiphilus_DSM_12809
Deferribacteres
Bact_Chrysiogenetes_YP_004112639.1_lysS_Desulfurispirillum_indicum_S5
Chrysiogenetes
Bact_Nitrospirae_YP_006766458.1_lysS_Leptospirillum_ferriphilum_ML-04
Bact_Nitrospirae_YP_003797698.1_lysS_Candidatus_Nitrospira_defluvii
Nitrospirae
Bact_Delta_YP_002491616.1_lysS_Anaeromyxobacter_dehalogenans_2CP-1
δ-protobacteria
Bact_Thermodesulfo_YP_004625451.1_lysS_Thermodesulfatator_indicus_DSM_15286
Bact_Thermodesulfo_YP_004628582.1_lysS_Thermodesulfobacterium_geofontis_OPF15
Thermodesulfobacteria
Bact_Nitrospirae_YP_002249051.1_lysS_Thermodesulfovibrio_yellowstonii_DSM_11347
Nitrospirae
Bact_Acidobacteria_YP_822317.1_lysS_Candidatus_Solibacter_usitatus_Ellin6076
Bact_Acidobacteria_YP_006421202.1_lysS_Terriglobus_roseus_DSM_18391
Acidobacteria
Bact_Planctomycetes_YP_004269758.1_lysS_Planctomyces_brasiliensis_DSM_5305
Bact_Planctomycetes_YP_003628881.1_lysS_Planctomyces_limnophilus_DSM_3776
Bact_Planctomycetes_YP_005445302.1_lysS_Phycisphaera_mikurensis_NBRC_102666
Planctomycetes
Bact_Chlamydiae_YP_001818966.1_lysS_Opitutus_terrae_PB90-1
Bact_Chlamydiae_YP_003548742.1_lysS_Coraliomargarita_akajimensis_DSM_45221
Bact_Chlamydiae_YP_001877413.1_lysS_Akkermansia_muciniphila_ATCC_BAA-835
Chlamydiae
Bact_Spirochaetes_WP_014803325.1_lysS_Turneriella
Bact_Spirochaetes_WP_016544242.1_lysS_Leptospira
Spirochaetes
Bact_Fusobacteria_YP_003163055.1_lysS_Leptotrichia_buccalis_C-1013-b
Bact_Fusobacteria_YP_003306838.1_lysS_Sebaldella_termitidis_ATCC_33386
Fusobacteria
Bact_Spirochaetes_WP_015274115.1_lysS_Brachyspira_pilosicoli
Spirochaetes
Bact_Alpha_YP_002545712.1_lysS_Agrobacterium_radiobacter_K84
Bact_Alpha_WP_007700724.1_lysS_Rhizobium_sp_AP16
Bact_Alpha_WP_077504855.1_lysS_Sinorhizobium_sp_A49
Bact_Alpha_WP_058325289.1_lysS_Sinorhizobium_sp_GL28
Bact_Alpha_OKP78921.1_lysS_Ensifer_adhaerens
α-protobacteria
Animal_005973119.1_lysS_Pantholops_hodgsonii
Animalia
Chromer_9397_lysS_Chromera_velia
Nucl. (Wolf PSORT: 12)
Chromer_4678_lysS_Vitrella_brassicaformis
Mito. (mitopro: 0.5)
Chromerids
Apico_220350_lysS_Toxoplasma_gondii
Mito. (mitopro: 0.9)
Apicomplexa
Diat_225827_lysS_Fragilariopsis_cylindrus
Diat_32202_lysS_Phaeodactylum_tricornutum
Plas.|Mito. (Gillian et al.)
Diatoms
Heterok_33044_lysS_Thalassiosira_pseudonana
Plas.|Mito. (Gillian et al.)
Stramenopiles
Hapto_456253_lysS_Emiliania_huxleyi
Haptophyta
Heterok_59065_lysS_Aureococcus_anophagefferens
Heterok_298713460_lysS_Ectocarpus_siliculosus
Stramenopiles
Rhodo_005709851_lysS_Chondrus_crispus
Rhodophyta
Rhiza_57451_lysS_Bigelowiella_natans
Rhizaria
Rhodo_544214646_lysS_Cyanidioschyzon_merolae
Rhodophyta
Cyano_YP_323653.1_lysS_Anabaena_variabilis_ATCC_29413
Cyano_YP_007051811.1_lysS_Nostoc_sp._PCC_7107
Cyano_YP_007054496.1_lysS_Rivularia_sp._PCC_7116
Cyano_YP_007111159.1_lysS_Geitlerinema_sp._PCC_7407
Cyano_YP_002371091.1_lysS_Cyanothece_sp._PCC_8801
Cyanobacteria
Crypto_158801_lysS_Guillardia_theta
Cryptophyta
Plant_003575390.1_lysS_Brachypodium_distachyon
Plant_020175196.1_lysS_Aegilops_tauschii
Plant_48716234_lysS_Oryza_sativa
Plant_002466144.1_lysS_Sorghum_bicolor
Plant_KMZ61004.1_lysS_Zostera_marina
Plant_009339189.1_lysS_Pyrus_bretschneideri
Plant_012844227.1_lysS_Erythranthe_guttata
Plant_AT3G13490_lysS_Arabidopsis_thaliana
Plas.Mito. (SUBA3)
Green Plants
Algae_159466544_lysS_Chlamydomonas_reinhardtii
Green Algae
Rhodo_005705592_lysS_Galdieria_sulphuraria
Rhodophyta
Bact_Actino_YP_008663661.1_lysS_Adlercreutzia_equolifaciens_DSM_19450
Bact_Actino_YP_003801616.1_lysS_Olsenella_uli_DSM_7084
Bact_Actino_YP_007871909.1_lysS_Propionibacterium_avidum_44067
Bact_Actino_YP_007870596.1_lysS_Propionibacterium_avidum_44067
Bact_Actino_YP_007565135.1_lysS_Ilumatobacter_coccineus_YM16-304
Actinobacteria
Bact_Tenericutes_NP_950841.1_lysS_Onion_yellows_phytoplasma_OY-M
Bact_Tenericutes_YP_001799121.1_lysS_Candidatus_Phytoplasma_australiense
Bact_Tenericutes_YP_001621355.1_lysS_Acholeplasma_laidlawii_PG-8A
Tenericutes
Bact_Firmicutes_YP_008909634.1_lysS_Paenibacillus_polymyxa_CR1
Firmicutes
Bact_Fusobacteria_YP_008476300.1_lysS_Fusobacterium_nucleatum_subsp._vincentii_3_1_36A2
Fusobacteria
Bact_Firmicutes_YP_699721.1_lysS_Clostridium_perfringens_SM101
Bact_Firmicutes_YP_006909309.1_lysS_Dehalobacter_sp._DCA
Firmicutes
Bact_Synergistetes_YP_003317632.1_lysS_Thermanaerovibrio_acidaminovorans_DSM_6589
Bact_Synergistetes_YP_003554217.1_lysS_Aminobacterium_colombiense_DSM_12261
Synergistetes
Bact_Caldiserica_YP_005473475.1_lysS_Caldisericum_exile_AZM16c01
Caldiserica
Bact_Thermotogae_YP_001410658.1_lysS_Fervidobacterium_nodosum_Rt17-B1
Bact_Thermotogae_YP_001306610.1_lysS_Thermosipho_melanesiensis_BI429
Bact_Thermotogae_YP_002941715.1_lysS_Kosmotoga_olearia_TBF_19.5.1
Thermotogae
Chromer_7175_lysS_Chromera_velia
Cyto. (Wolf PSORT: 15)
Chromer_20258_lysS_Chromera_velia
Nucl.Cyto. (Wolf PSORT: 8.5)
Chromer_6201_lysS_Vitrella_brassicaformis
Cyto. (Wolf PSORT: 9.5)
Chromerids
Apico_205710_lysS_Toxoplasma_gondii
Mito. (mitopro: 0.8)
Apico_209877783_lysS_Cryptosporidium_muris
Apicomplexa
Perkinsozoa_002781041.1_lysS_Perkinsus_marinus
Perkinsozoa_002780649.1_lysS_Perkinsus_marinus
Perkinsozoa_002766916.1_lysS_Perkinsus_marinus
Perkinsozoa
Cilio_145506673_lysS_Paramecium_tetraurelia
Cilio_145518141_lysS_Paramecium_tetraurelia
Cilio_118399585_lysS_Tetrahymena_thermophila
Ciliophora
Kineto_68125392_lysS_Leishmania_major
Kineto_70803024_lysS_Trypanosoma_brucei
Kinetoplastida
Excav_123447017_lysS_Trichomonas_vaginalis
Excavata
Amoeb_66812996_lysS_Dictyostelium_discoideum
Amoebozoa
Crypto_157671_lysS_Guillardia_theta
Cyto. (Gillian et al.)
Cryptophyta
Mam_ENSG00000065427_lysS_Homo_sapiens
Cyto. (GO)
3 Mito. (GO)
2 Unknown
Animal_71895483_lysS_Gallus_gallus
Animal_49900533_lysS_Danio_rerio
Animal_020378154.1_lysS_Rhincodon_typus
Animal_014340879.1_lysS_Latimeria_chalumnae
Animal_17535925_lysS_Caenorhabditis_elegans
Fung_85086144_lysS_Neurospora_crassa
Fung_6320242_lysS_Saccharomyces_cerevisiae
Fung_014181726.1_lysS_Trichosporon_asahii
Opisthokonta
Rhiza_88184_lysS_Bigelowiella_natans
Rhizaria
Algae_36567_lysS_Ostreococcus_tauri
Green Algae
Hapto_426543_lysS_Emiliania_huxleyi
Haptophyta
Oomy_71183_lysS_Phytophthora_ramorum
Oomy_262097915_lysS_Phytophthora_infestans
Oomy_695463275_lysS_Phytophthora_sojae
Oomycetes
Heterok_299472298_lysS_Ectocarpus_siliculosus
Stramenopiles
Diat_168292_lysS_Fragilariopsis_cylindrus
Diat_42289_lysS_Phaeodactylum_tricornutum
Cyto. (Gillian et al.)
Diatoms
Heterok_29232_lysS_Thalassiosira_pseudonana
Cyto. (Gillian et al.)
Heterok_1254_lysS_Aureococcus_anophagefferens
Stramenopiles
Rhodo_544213984_lysS_Cyanidioschyzon_merolae
Rhodo_005704525_lysS_Galdieria_sulphuraria
Rhodo_005717803_lysS_Chondrus_crispus
Rhodophyta
Plant_AT3G11710_lysS_Arabidopsis_thaliana
Nucl.Cyto. (SUBA3)
Plant_108709560_lysS_Oryza_sativa
Green Plants
Algae_159478809_lysS_Chlamydomonas_reinhardtii
Green Algae
Crypto_68810_lysS_Guillardia_theta
PPC (Gillian et al.)
Cryptophyta
Oomy_695446523_lysS_Phytophthora_sojae
Oomycetes
Crypto_144009_lysS_Guillardia_theta
Mito. (Gillian et al.)
Cryptophyta
Bact_Thermus_YP_004202102.1_lysS_Thermus_scotoductus_SA-01
Bact_Thermus_YP_004057676.1_lysS_Oceanithermus_profundus_DSM_14977
Bact_Thermus_YP_002785093.1_lysS_Deinococcus_deserti_VCD115
Thermus
Bact_Aquificae_YP_003473152.1_lysS_Thermocrinis_albus_DSM_14484
Bact_Aquificae_YP_002121661.1_lysS_Hydrogenobaculum_sp._Y04AAS1
Aquificae
Bact_Aquificae_YP_001931753.1_lysS_Sulfurihydrogenibium_sp._YO3AOP1
Bact_Chlorobi_YP_006866581.1_lysS_Psychroflexus_torquis_ATCC_700755
Bact_Chlorobi_YP_213196.1_lysS_Bacteroides_fragilis_NCTC_9343
Bact_Chlorobi_YP_005012186.1_lysS_Niastella_koreensis_GR20-10
Bacteroidetes
Bact_Gemmati_YP_002761091.1_lysS_Gemmatimonas_aurantiaca_T-27
Gemmatimonadetes
Bact_Elusimicrobia_YP_001874962.1_lysS_Elusimicrobium_minutum_Pei191
Elusimicrobia
Bact_Delta_YP_007023241.1_lysS_Bdellovibrio_bacteriovorus_str._Tiberius
δ-protobacteria
Bact_Chloroflexi_YP_004175615.1_lysS_Anaerolinea_thermophila_UNI-1
Bact_Chloroflexi_YP_005440050.1_lysS_Caldilinea_aerophila_DSM_14535_=_NBRC_104270
Bact_Chloroflexi_YP_002463668.1_lysS_Chloroflexus_aggregans_DSM_9485
Chloroflexi
Bact_Crenarchaeota_YP_003815540.1_lysS_Acidilobus_saccharovorans_345-15
Bact_Crenarchaeota_YP_007174517.1_lysS_Caldisphaera_lagunensis_DSM_15908
Bact_Crenarchaeota_YP_001541004.1_lysS_Caldivirga_maquilingensis_IC-167
Archae_Eury_YP_007246471.1_lysS_Aciduliprofundum_sp._MAR08-339
Archae_Eury_YP_003482992.1_lysS_Aciduliprofundum_boonei_T469
Archaea
0.3

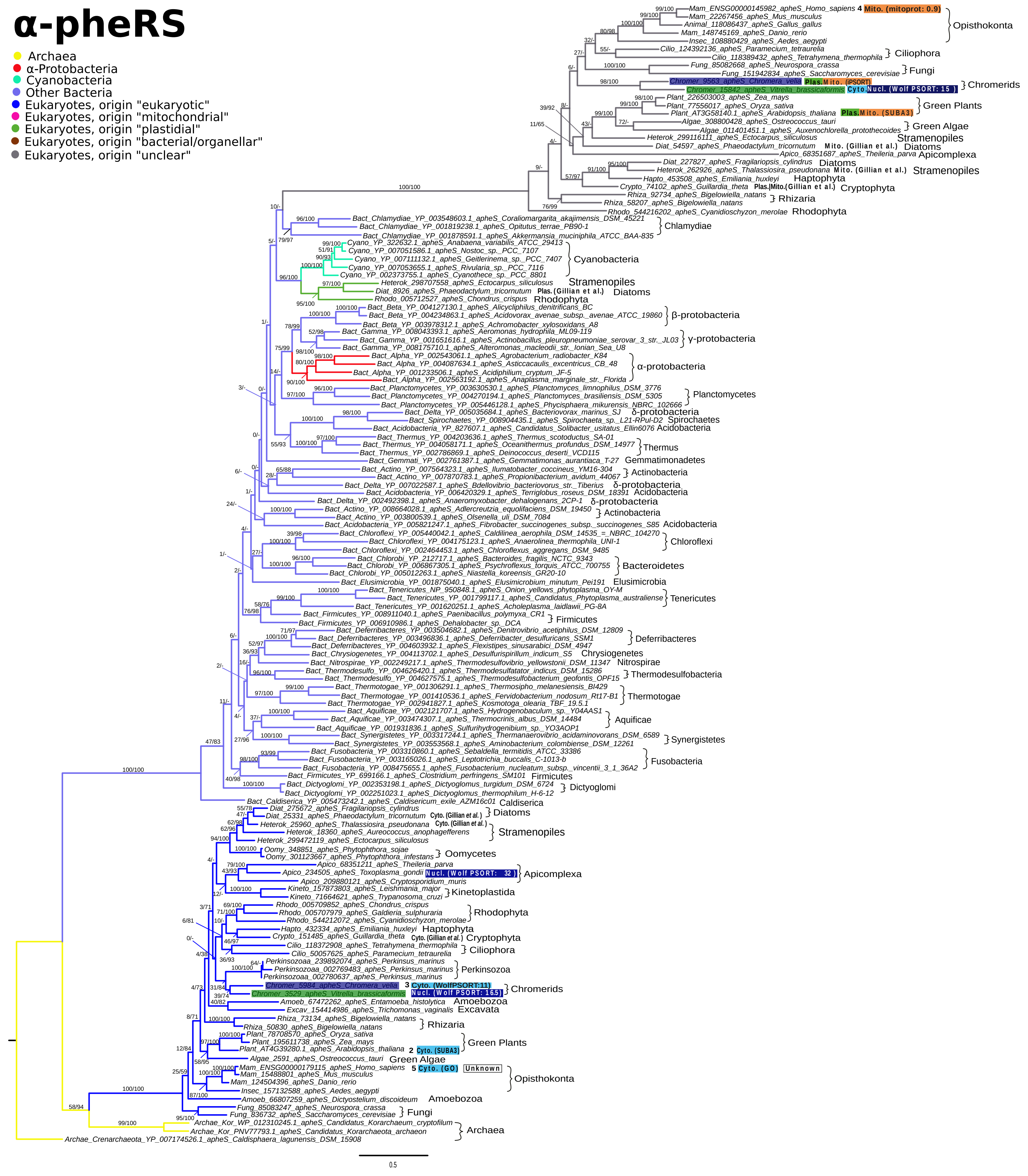

α-pheRS
Archaea
α-Protobacteria
Cyanobacteria
Other Bacteria
Eukaryotes, origin "eukaryotic"
Eukaryotes, origin "mitochondrial"
Eukaryotes, origin "plastidial"
Eukaryotes, origin "bacterial/organellar"
Eukaryotes, origin "unclear"
Mam_ENSG00000145982_apheS_Homo_sapiens 4 Mito. (mitoprot: 0.9)
Mam_22267456_apheS_Mus_musculus
Animal_118086437_apheS_Gallus_gallus
Mam_148745169_apheS_Danio_rerio
Insec_108880429_apheS_Aedes_aegypti
Opisthokonta
Cilio_124392136_apheS_Paramecium_tetraurelia
Cilio_118389432_apheS_Tetrahymena_thermophila
Ciliophora
Fung_85082668_apheS_Neurospora_crassa
Fung_151942834_apheS_Saccharomyces_cerevisiae
Fungi
Chromer_9563_apheS_Chromera_velia Plas.Mito. (iPSORT)
Chromer_15842_apheS_Vitrella_brassicaformis Cyto.Nucl. (Wolf PSORT: 15)
Chromerids
Plant_226503003_apheS_Zea_mays
Plant_77556017_apheS_Oryza_sativa
Plant_AT3G58140.1_apheS_Arabidopsis_thaliana Plas.Mito. (SUBA3)
Green Plants
Algae_308800428_apheS_Ostreococcus_tauri
Algae_011401451.1_apheS_Auxenochlorella_protothecoides
Green Algae
Heterok_299116111_apheS_Ectocarpus_siliculosus
Stramenopiles
Diat_54597_apheS_Phaeodactylum_tricornutum Mito. (Gillian et al.)
Diatoms
Apico_68351687_apheS_Theileria_parva
Apicomplexa
Diat_227827_apheS_Fragilariopsis_cylindrus
Diatoms
Heterok_262926_apheS_Thalassiosira_pseudonana Mito. (Gillian et al.)
Stramenopiles
Hapto_453508_apheS_Emiliania_huxleyi
Haptophyta
Crypto_74102_apheS_Guillardia_theta Plas.|Mito.(Gillian et al.)
Cryptophyta
Rhiza_92734_apheS_Bigelowiella_natans
Rhiza_58207_apheS_Bigelowiella_natans
Rhizaria
Rhodo_544216202_apheS_Cyanidioschyzon_merolae
Rhodophyta
Bact_Chlamydiae_YP_003548603.1_apheS_Coraliomargarita_akajimensis_DSM_45221
Bact_Chlamydiae_YP_001819238.1_apheS_Opitutus_terrae_PB90-1
Bact_Chlamydiae_YP_001878591.1_apheS_Akkermansia_muciniphila_ATCC_BAA-835
Chlamydiae
Cyano_YP_322632.1_apheS_Anabaena_variabilis_ATCC_29413
Cyano_YP_007051586.1_apheS_Nostoc_sp._PCC_7107
Cyano_YP_007111132.1_apheS_Geitlerinema_sp._PCC_7407
Cyano_YP_007053655.1_apheS_Rivularia_sp._PCC_7116
Cyano_YP_002373755.1_apheS_Cyanothece_sp._PCC_8801
Cyanobacteria
Heterok_298707558_apheS_Ectocarpus_siliculosus
Stramenopiles
Diat_8926_apheS_Phaeodactylum_tricornutum Plas. (Gillian et al.)
Diatoms
Rhodo_005712527_apheS_Chondrus_crispus
Rhodophyta
Bact_Beta_YP_004127130.1_apheS_Alicycliphilus_denitrificans_BC
Bact_Beta_YP_004234863.1_apheS_Acidovorax_avenae_subsp._avenae_ATCC_19860
Bact_Beta_YP_003978312.1_apheS_Achromobacter_xylosoxidans_A8
β-protobacteria
Bact_Gamma_YP_008043393.1_apheS_Aeromonas_hydrophila_ML09-119
Bact_Gamma_YP_001651616.1_apheS_Actinobacillus_pleuropneumoniae_serovar_3_str._JL03
Bact_Gamma_YP_008175710.1_apheS_Alteromonas_macleodii_str._Ionian_Sea_U8
γ-protobacteria
Bact_Alpha_YP_002543061.1_apheS_Agrobacterium_radiobacter_K84
Bact_Alpha_YP_004087634.1_apheS_Asticcacaulis_excentricus_CB_48
Bact_Alpha_YP_001233506.1_apheS_Acidiphilium_cryptum_JF-5
Bact_Alpha_YP_002563192.1_apheS_Anaplasma_marginale_str._Florida
α-protobacteria
Bact_Planctomycetes_YP_003630530.1_apheS_Planctomyces_limnophilus_DSM_3776
Bact_Planctomycetes_YP_004270194.1_apheS_Planctomyces_brasiliensis_DSM_5305
Bact_Planctomycetes_YP_005446128.1_apheS_Phycisphaera_mikurensis_NBRC_102666
Planctomycetes
Bact_Delta_YP_005035684.1_apheS_Bacteriovorax_marinus_SJ
δ-protobacteria
Bact_Spirochaetes_YP_008904435.1_apheS_Spirochaeta_sp._L21-RPul-D2
Spirochaetes
Bact_Acidobacteria_YP_827607.1_apheS_Candidatus_Solibacter_usitatus_Ellin6076
Acidobacteria
Bact_Thermus_YP_004203636.1_apheS_Thermus_scotoductus_SA-01
Bact_Thermus_YP_004058171.1_apheS_Oceanithermus_profundus_DSM_14977
Bact_Thermus_YP_002786869.1_apheS_Deinococcus_deserti_VCD115
Thermus
Bact_Gemmati_YP_002761387.1_apheS_Gemmatimonas_aurantiaca_T-27
Gemmatimonadetes
Bact_Actino_YP_007564323.1_apheS_Ilumatobacter_coccineus_YM16-304
Bact_Actino_YP_007870783.1_apheS_Propionibacterium_avidum_44067
Actinobacteria
Bact_Delta_YP_007022587.1_apheS_Bdellovibrio_bacteriovorus_str._Tiberius
δ-protobacteria
Bact_Acidobacteria_YP_006420329.1_apheS_Terriglobus_roseus_DSM_18391
Acidobacteria
Bact_Delta_YP_002492398.1_apheS_Anaeromyxobacter_dehalogenans_2CP-1
δ-protobacteria
Bact_Actino_YP_008664028.1_apheS_Adlercreutzia_equolifaciens_DSM_19450
Bact_Actino_YP_003800539.1_apheS_Olsenella_uli_DSM_7084
Actinobacteria
Bact_Acidobacteria_YP_005821247.1_apheS_Fibrobacter_succinogenes_subsp._succinogenes_S85
Acidobacteria
Bact_Chloroflexi_YP_005440042.1_apheS_Caldilinea_aerophila_DSM_14535_=_NBRC_104270
Bact_Chloroflexi_YP_004175123.1_apheS_Anaerolinea_thermophila_UNI-1
Bact_Chloroflexi_YP_002464453.1_apheS_Chloroflexus_aggregans_DSM_9485
Chloroflexi
Bact_Chlorobi_YP_212717.1_apheS_Bacteroides_fragilis_NCTC_9343
Bact_Chlorobi_YP_006867305.1_apheS_Psychroflexus_torquis_ATCC_700755
Bact_Chlorobi_YP_005012263.1_apheS_Niastella_koreensis_GR20-10
Bacteroidetes
Bact_Elusimicrobia_YP_001875040.1_apheS_Elusimicrobium_minutum_Pei191
Elusimicrobia
Bact_Tenericutes_NP_950848.1_apheS_Onion_yellows_phytoplasma_OY-M
Bact_Tenericutes_YP_001799117.1_apheS_Candidatus_Phytoplasma_australiense
Bact_Tenericutes_YP_001620251.1_apheS_Acholeplasma_laidlawii_PG-8A
Tenericutes
Bact_Firmicutes_YP_008911040.1_apheS_Paenibacillus_polymyxa_CR1
Bact_Firmicutes_YP_006910986.1_apheS_Dehalobacter_sp._DCA
Firmicutes
Bact_Deferribacteres_YP_003504682.1_apheS_Denitrovibrio_acetiphilus_DSM_12809
Bact_Deferribacteres_YP_003496836.1_apheS_Deferribacter_desulfuricans_SSM1
Bact_Deferribacteres_YP_004603932.1_apheS_Flexistipes_sinusarabici_DSM_4947
Deferribacteres
Bact_Chrysiogenetes_YP_004113702.1_apheS_Desulfurispirillum_indicum_S5
Chrysiogenetes
Bact_Nitrospirae_YP_002249217.1_apheS_Thermodesulfovibrio_yellowstonii_DSM_11347
Nitrospirae
Bact_Thermodesulfo_YP_004626420.1_apheS_Thermodesulfatator_indicus_DSM_15286
Bact_Thermodesulfo_YP_004627575.1_apheS_Thermodesulfobacterium_geofontis_OPF15
Thermodesulfobacteria
Bact_Thermotogae_YP_001306291.1_apheS_Thermosipho_melanesiensis_BI429
Bact_Thermotogae_YP_001410536.1_apheS_Fervidobacterium_nodosum_Rt17-B1
Bact_Thermotogae_YP_002941827.1_apheS_Kosmotoga_olearia_TBF_19.5.1
Thermotogae
Bact_Aquificae_YP_002121707.1_apheS_Hydrogenobaculum_sp._Y04AAS1
Bact_Aquificae_YP_003474307.1_apheS_Thermocrinis_albus_DSM_14484
Bact_Aquificae_YP_001931836.1_apheS_Sulfurihydrogenibium_sp._YO3AOP1
Aquificae
Bact_Synergistetes_YP_003317244.1_apheS_Thermanaerovibrio_acidaminovorans_DSM_6589
Bact_Synergistetes_YP_003553568.1_apheS_Aminobacterium_colombiense_DSM_12261
Synergistetes
Bact_Fusobacteria_YP_003310860.1_apheS_Sebaldella_termitidis_ATCC_33386
Bact_Fusobacteria_YP_003165026.1_apheS_Leptotrichia_buccalis_C-1013-b
Bact_Fusobacteria_YP_008475655.1_apheS_Fusobacterium_nucleatum_subsp._vincentii_3_1_36A2
Fusobacteria
Bact_Firmicutes_YP_699166.1_apheS_Clostridium_perfringens_SM101
Firmicutes
Bact_Dictyoglomi_YP_002353198.1_apheS_Dictyoglomus_turgidum_DSM_6724
Bact_Dictyoglomi_YP_002251023.1_apheS_Dictyoglomus_thermophilum_H-6-12
Dictyoglomi
Bact_Caldiserica_YP_005473242.1_apheS_Caldisericum_exile_AZM16c01
Caldiserica
Diat_275672_apheS_Fragilariopsis_cylindrus
Diat_25331_apheS_Phaeodactylum_tricornutum Cyto. (Gillian et al.)
Diatoms
Heterok_25960_apheS_Thalassiosira_pseudonana Cyto. (Gillian et al.)
Heterok_18360_apheS_Aureococcus_anophagefferens
Heterok_299472119_apheS_Ectocarpus_siliculosus
Stramenopiles
Oomy_348851_apheS_Phytophthora_sojae
Oomy_301123667_apheS_Phytophthora_infestans
Oomycetes
Apico_68351211_apheS_Theileria_parva
Apico_234505_apheS_Toxoplasma_gondii Nucl. (Wolf PSORT: 32)
Apico_209880121_apheS_Cryptosporidium_muris
Apicomplexa
Kineto_157873803_apheS_Leishmania_major
Kineto_71664621_apheS_Trypanosoma_cruzi
Kinetoplastida
Rhodo_005709852_apheS_Chondrus_crispus
Rhodo_005707979_apheS_Galdieria_sulphuraria
Rhodo_544212072_apheS_Cyanidioschyzon_merolae
Rhodophyta
Hapto_432334_apheS_Emiliania_huxleyi
Haptophyta
Crypto_151485_apheS_Guillardia_theta Cyto. (Gillian et al.)
Cryptophyta
Cilio_118372908_apheS_Tetrahymena_thermophila
Cilio_50057625_apheS_Paramecium_tetraurelia
Ciliophora
Perkinsozoaa_239892074_apheS_Perkinsus_marinus
Perkinsozoaa_002769483_apheS_Perkinsus_marinus
Perkinsozoaa_002780637_apheS_Perkinsus_marinus
Perkinsozoa
Chromer_5984_apheS_Chromera_velia 3 Cyto. (WolfPSORT:11)
Chromer_3529_apheS_Vitrella_brassicaformis Nucl. (Wolf PSORT: 16.5)
Chromerids
Amoeb_67472262_apheS_Entamoeba_histolytica
Amoebozoa
Excav_154414986_apheS_Trichomonas_vaginalis
Excavata
Rhiza_73134_apheS_Bigelowiella_natans
Rhiza_50830_apheS_Bigelowiella_natans
Rhizaria
Plant_78708570_apheS_Oryza_sativa
Plant_195611738_apheS_Zea_mays
Plant_AT4G39280.1_apheS_Arabidopsis_thaliana 2 Cyto. (SUBA3)
Green Plants
Algae_2591_apheS_Ostreococcus_tauri
Green Algae
Mam_ENSG00000179115_apheS_Homo_sapiens 5 Cyto. (GO) Unknown
Mam_15488801_apheS_Mus_musculus
Mam_124504396_apheS_Danio_rerio
Insec_157132588_apheS_Aedes_aegypti
Opisthokonta
Amoeb_66807259_apheS_Dictyostelium_discoideum
Amoebozoa
Fung_85083247_apheS_Neurospora_crassa
Fung_836732_apheS_Saccharomyces_cerevisiae
Fungi
Archae_Kor_WP_012310245.1_apheS_Candidatus_Korarchaeum_cryptofilum
Archae_Kor_PNV77793.1_apheS_Candidatus_Korarchaeota_archaeon
Archaea
Archae_Crenarchaeota_YP_007174526.1_apheS_Caldisphaera_lagunensis_DSM_15908
0.5

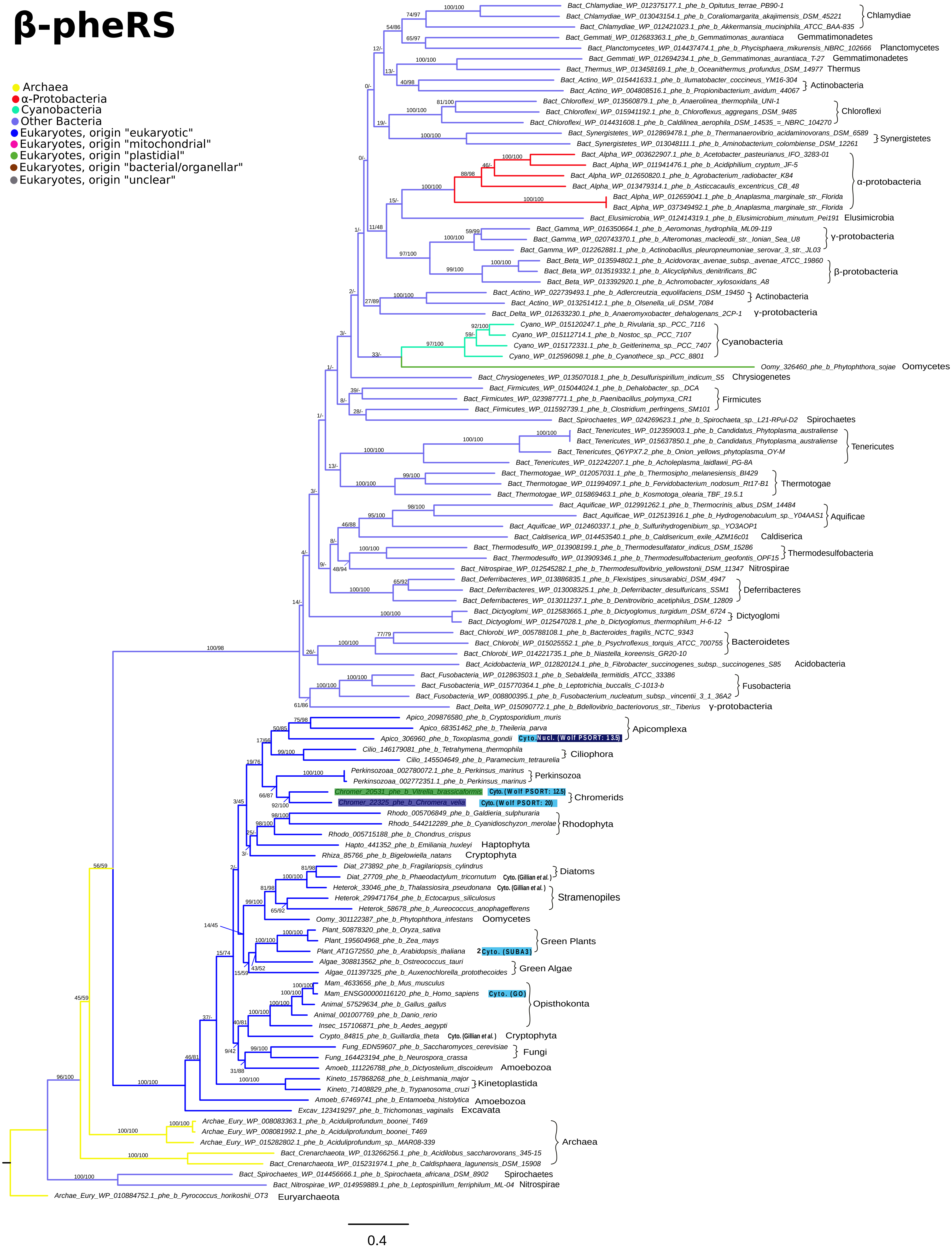

β-pheRS
Archaea
α-Protobacteria
Cyanobacteria
Other Bacteria
Eukaryotes, origin "eukaryotic"
Eukaryotes, origin "mitochondrial"
Eukaryotes, origin "plastidial"
Eukaryotes, origin "bacterial/organellar"
Eukaryotes, origin "unclear"
Bact_Chlamydiae_WP_012375177.1_phe_b_Opitutus_terrae_PB90-1
Bact_Chlamydiae_WP_013043154.1_phe_b_Coraliomargarita_akajimensis_DSM_45221
Bact_Chlamydiae_WP_012421023.1_phe_b_Akkermansia_muciniphila_ATCC_BAA-835
Chlamydiae
Bact_Gemmati_WP_012683363.1_phe_b_Gemmatimonas_aurantiaca
Gemmatimonadetes
Bact_Planctomycetes_WP_014437474.1_phe_b_Phycisphaera_mikurensis_NBRC_102666
Planctomycetes
Bact_Gemmati_WP_012694234.1_phe_b_Gemmatimonas_aurantiaca_T-27
Gemmatimonadetes
Bact_Thermus_WP_013458169.1_phe_b_Oceanithermus_profundus_DSM_14977
Thermus
Bact_Actino_WP_015441633.1_phe_b_Ilumatobacter_coccineus_YM16-304
Bact_Actino_WP_004808516.1_phe_b_Propionibacterium_avidum_44067
Actinobacteria
Bact_Chloroflexi_WP_013560879.1_phe_b_Anaerolinea_thermophila_UNI-1
Bact_Chloroflexi_WP_015941192.1_phe_b_Chloroflexus_aggregans_DSM_9485
Bact_Chloroflexi_WP_014431608.1_phe_b_Caldilinea_aerophila_DSM_14535_=_NBRC_104270
Chloroflexi
Bact_Synergistetes_WP_012869478.1_phe_b_Thermanaerovibrio_acidaminovorans_DSM_6589
Bact_Synergistetes_WP_013048111.1_phe_b_Aminobacterium_colombiense_DSM_12261
Synergistetes
Bact_Alpha_WP_003622907.1_phe_b_Acetobacter_pasteurianus_IFO_3283-01
Bact_Alpha_WP_011941476.1_phe_b_Acidiphilium_cryptum_JF-5
Bact_Alpha_WP_012650820.1_phe_b_Agrobacterium_radiobacter_K84
Bact_Alpha_WP_013479314.1_phe_b_Asticcacaulis_excentricus_CB_48
Bact_Alpha_WP_012659041.1_phe_b_Anaplasma_marginale_str._Florida
Bact_Alpha_WP_037349492.1_phe_b_Anaplasma_marginale_str._Florida
α-protobacteria
Bact_Elusimicrobia_WP_012414319.1_phe_b_Elusimicrobium_minutum_Pei191
Elusimicrobia
Bact_Gamma_WP_016350664.1_phe_b_Aeromonas_hydrophila_ML09-119
Bact_Gamma_WP_020743370.1_phe_b_Alteromonas_macleodii_str._Ionian_Sea_U8
Bact_Gamma_WP_012262881.1_phe_b_Actinobacillus_pleuropneumoniae_serovar_3_str._JL03
γ-protobacteria
Bact_Beta_WP_013594802.1_phe_b_Acidovorax_avenae_subsp._avenae_ATCC_19860
Bact_Beta_WP_013519332.1_phe_b_Alicycliphilus_denitrificans_BC
Bact_Beta_WP_013392920.1_phe_b_Achromobacter_xylosoxidans_A8
β-protobacteria
Bact_Actino_WP_022739493.1_phe_b_Adlercreutzia_equolifaciens_DSM_19450
Bact_Actino_WP_013251412.1_phe_b_Olsenella_uli_DSM_7084
Actinobacteria
Bact_Delta_WP_012633230.1_phe_b_Anaeromyxobacter_dehalogenans_2CP-1
γ-protobacteria
Cyano_WP_015120247.1_phe_b_Rivularia_sp._PCC_7116
Cyano_WP_015112714.1_phe_b_Nostoc_sp._PCC_7107
Cyano_WP_015172331.1_phe_b_Geitlerinema_sp._PCC_7407
Cyano_WP_012596098.1_phe_b_Cyanothece_sp._PCC_8801
Cyanobacteria
Oomy_326460_phe_b_Phytophthora_sojae
Oomycetes
Bact_Chrysiogenetes_WP_013507018.1_phe_b_Desulfurispirillum_indicum_S5
Chrysiogenetes
Bact_Firmicutes_WP_015044024.1_phe_b_Dehalobacter_sp._DCA
Bact_Firmicutes_WP_023987771.1_phe_b_Paenibacillus_polymyxa_CR1
Bact_Firmicutes_WP_011592739.1_phe_b_Clostridium_perfringens_SM101
Firmicutes
Bact_Spirochaetes_WP_024269623.1_phe_b_Spirochaeta_sp._L21-RPul-D2
Spirochaetes
Bact_Tenericutes_WP_012359003.1_phe_b_Candidatus_Phytoplasma_australiense
Bact_Tenericutes_WP_015637850.1_phe_b_Candidatus_Phytoplasma_australiense
Bact_Tenericutes_Q6YPX7.2_phe_b_Onion_yellows_phytoplasma_OY-M
Bact_Tenericutes_WP_012242207.1_phe_b_Acholeplasma_laidlawii_PG-8A
Tenericutes
Bact_Thermotogae_WP_012057031.1_phe_b_Thermosipho_melanesiensis_BI429
Bact_Thermotogae_WP_011994097.1_phe_b_Fervidobacterium_nodosum_Rt17-B1
Bact_Thermotogae_WP_015869463.1_phe_b_Kosmotoga_olearia_TBF_19.5.1
Thermotogae
Bact_Aquificae_WP_012991262.1_phe_b_Thermocrinis_albus_DSM_14484
Bact_Aquificae_WP_012513916.1_phe_b_Hydrogenobaculum_sp._Y04AAS1
Bact_Aquificae_WP_012460337.1_phe_b_Sulfurihydrogenibium_sp._YO3AOP1
Aquificae
Bact_Caldiserica_WP_014453540.1_phe_b_Caldisericum_exile_AZM16c01
Caldiserica
Bact_Thermodesulfo_WP_013908199.1_phe_b_Thermodesulfatator_indicus_DSM_15286
Bact_Thermodesulfo_WP_013909346.1_phe_b_Thermodesulfobacterium_geofontis_OPF15
Thermodesulfobacteria
Bact_Nitrospirae_WP_012545282.1_phe_b_Thermodesulfovibrio_yellowstonii_DSM_11347
Nitrospirae
Bact_Deferribacteres_WP_013886835.1_phe_b_Flexistipes_sinusarabici_DSM_4947
Bact_Deferribacteres_WP_013008325.1_phe_b_Deferribacter_desulfuricans_SSM1
Bact_Deferribacteres_WP_013011237.1_phe_b_Denitrovibrio_acetiphilus_DSM_12809
Deferribacteres
Bact_Dictyoglomi_WP_012583665.1_phe_b_Dictyoglomus_turgidum_DSM_6724
Bact_Dictyoglomi_WP_012547028.1_phe_b_Dictyoglomus_thermophilum_H-6-12
Dictyoglomi
Bact_Chlorobi_WP_005788108.1_phe_b_Bacteroides_fragilis_NCTC_9343
Bact_Chlorobi_WP_015025552.1_phe_b_Psychroflexus_torquis_ATCC_700755
Bact_Chlorobi_WP_014221735.1_phe_b_Niastella_koreensis_GR20-10
Bacteroidetes
Bact_Acidobacteria_WP_012820124.1_phe_b_Fibrobacter_succinogenes_subsp._succinogenes_S85
Acidobacteria
Bact_Fusobacteria_WP_012863503.1_phe_b_Sebaldella_termitidis_ATCC_33386
Bact_Fusobacteria_WP_015770364.1_phe_b_Leptotrichia_buccalis_C-1013-b
Bact_Fusobacteria_WP_008800395.1_phe_b_Fusobacterium_nucleatum_subsp._vincentii_3_1_36A2
Fusobacteria
Bact_Delta_WP_015090772.1_phe_b_Bdellovibrio_bacteriovorus_str._Tiberius
γ-protobacteria
Apico_209876580_phe_b_Cryptosporidium_muris
Apico_68351462_phe_b_Theileria_parva
Apico_306960_phe_b_Toxoplasma_gondii
Cyto. Nucl. (Wolf PSORT: 13.5)
Apicomplexa
Cilio_146179081_phe_b_Tetrahymena_thermophila
Cilio_145504649_phe_b_Paramecium_tetraurelia
Ciliophora
Perkinsozoaa_002780072.1_phe_b_Perkinsus_marinus
Perkinsozoaa_002772351.1_phe_b_Perkinsus_marinus
Perkinsoza
Chromer_20531_phe_b_Vitrella_brassicaformis
Cyto. (Wolf PSORT: 12.5)
Chromer_22325_phe_b_Chromera_velia
Cyto. (Wolf PSORT: 20)
Chromerids
Rhodo_005706849_phe_b_Galdieria_sulphuraria
Rhodo_544212289_phe_b_Cyanidioschyzon_merolae
Rhodo_005715188_phe_b_Chondrus_crispus
Rhodophyta
Hapto_441352_phe_b_Emiliania_huxleyi
Haptophyta
Rhiza_85766_phe_b_Bigelowiella_natans
Cryptophyta
Diat_273892_phe_b_Fragilariopsis_cylindrus
Diat_27709_phe_b_Phaeodactylum_tricornutum
Cyto. (Gillian et al.)
Diatoms
Heterok_33046_phe_b_Thalassiosira_pseudonana
Cyto. (Gillian et al.)
Heterok_299471764_phe_b_Ectocarpus_siliculosus
Heterok_58678_phe_b_Aureococcus_anophagefferens
Stramenopiles
Oomy_301122387_phe_b_Phytophthora_infestans
Oomycetes
Plant_50878320_phe_b_Oryza_sativa
Plant_195604968_phe_b_Zea_mays
Plant_AT1G72550_phe_b_Arabidopsis_thaliana
2 Cyto. (SUBA3)
Green Plants
Algae_308813562_phe_b_Ostreococcus_tauri
Algae_011397325_phe_b_Auxenochlorella_protothecoides
Green Algae
Mam_4633656_phe_b_Mus_musculus
Mam_ENSG00000116120_phe_b_Homo_sapiens
Cyto. (GO)
Animal_57529634_phe_b_Gallus_gallus
Animal_001007769_phe_b_Danio_rerio
Insec_157106871_phe_b_Aedes_aegypti
Opisthokonta
Crypto_84815_phe_b_Guillardia_theta
Cyto. (Gillian et al.)
Cryptophyta
Fung_EDN59607_phe_b_Saccharomyces_cerevisiae
Fung_164423194_phe_b_Neurospora_crassa
Fungi
Amoeb_111226788_phe_b_Dictyostelium_discoideum
Amoebozoa
Kineto_157868268_phe_b_Leishmania_major
Kineto_71408829_phe_b_Trypanosoma_cruzi
Kinetoplastida
Amoeb_67469741_phe_b_Entamoeba_histolytica
Amoebozoa
Excav_123419297_phe_b_Trichomonas_vaginalis
Excavata
Archae_Eury_WP_008083363.1_phe_b_Aciduliprofundum_boonei_T469
Archae_Eury_WP_008081992.1_phe_b_Aciduliprofundum_boonei_T469
Archae_Eury_WP_015282802.1_phe_b_Aciduliprofundum_sp._MAR08-339
Bact_Crenarchaeota_WP_013266256.1_phe_b_Acidilobus_saccharovorans_345-15
Bact_Crenarchaeota_WP_015231974.1_phe_b_Caldisphaera_lagunensis_DSM_15908
Archaea
Bact_Spirochaetes_WP_014456666.1_phe_b_Spirochaeta_africana_DSM_8902
Spirochaetes
Bact_Nitrospirae_WP_014959889.1_phe_b_Leptospirillum_ferriphilum_ML-04
Nitrospirae
Archae_Eury_WP_010884752.1_phe_b_Pyrococcus_horikoshii_OT3
Euryarchaeota
0.4

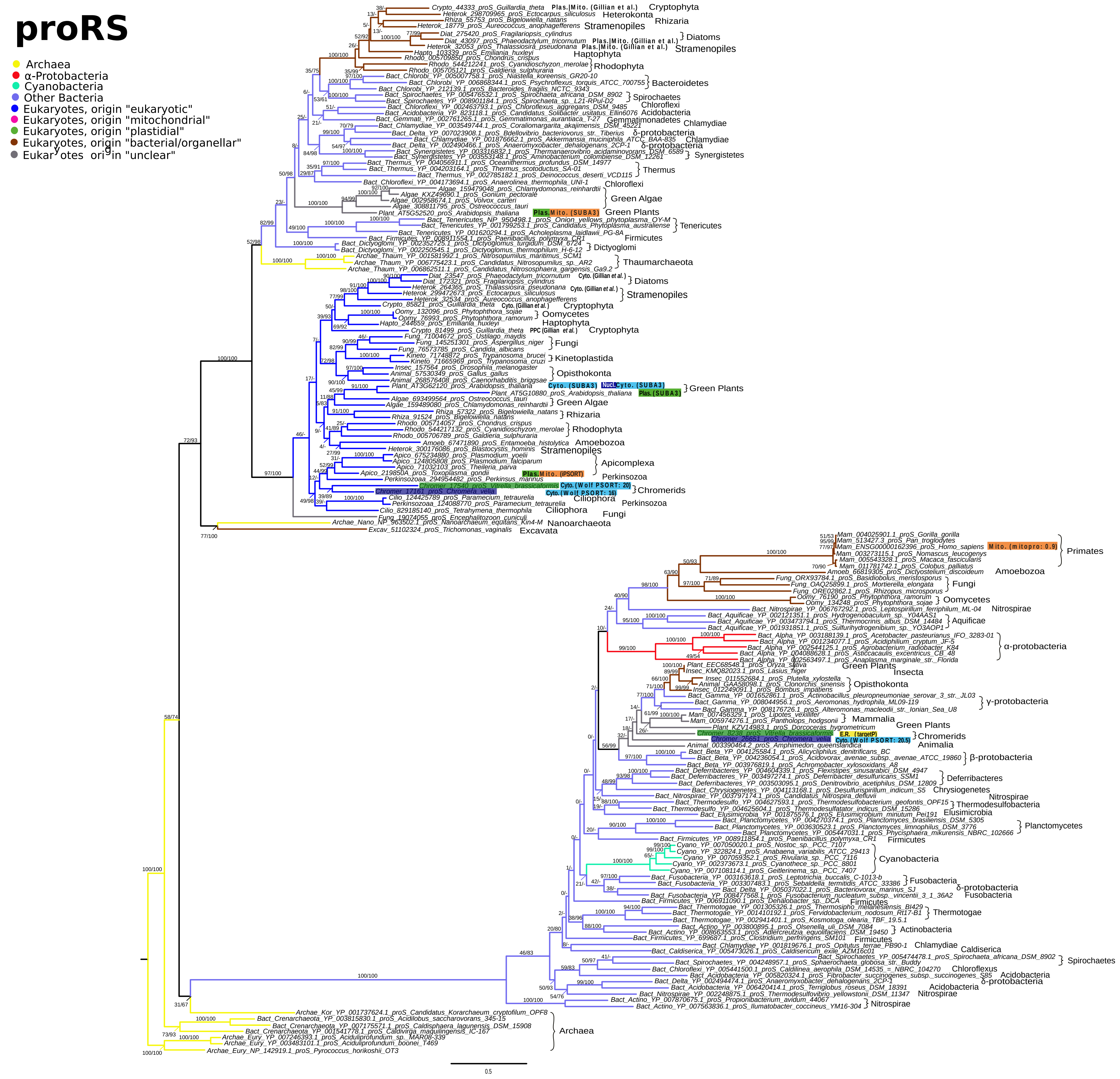
proRS
Archaea
α-Protobacteria
Cyanobacteria
Other Bacteria
Eukaryotes, origin "eukaryotic"
Eukaryotes, origin "mitochondrial"
Eukaryotes, origin "plastidial"
Eukaryotes, origin "bacterial/organellar"
Eukaryotes, origin "unclear"
Cryptophyta
Heterokonta
Rhizaria
Stramenopiles
Diatoms
Haptophyta
Rhodophyta
Bacteroidetes
Spirochaetes
Chloroflexi
Acidobacteria
Gemmatimonadetes
Chlamydiae
δ-protobacteria
Synergistetes
Thermus
Green Algae
Green Plants
Tenericutes
Firmicutes
Dictyoglomi
Thaumarchaeota
Oomycetes
Fungi
Kinetoplastida
Opisthokonta
Amoebozoa
Apicomplexa
Perkinsozoa
Chromerids
Ciliophora
Nanoarchaeota
Excavata
Primates
Nitrospirae
Aquificae
α-protobacteria
Insecta
γ-protobacteria
Mammalia
Animalia
β-protobacteria
Deferribacteres
Chrysiogenetes
Thermodesulfobacteria
Elusimicrobia
Planctomycetes
Cyanobacteria
Fusobacteria
Thermotogae
Actinobacteria
Caldiserica
Chloroflexus
Archaea
0.5

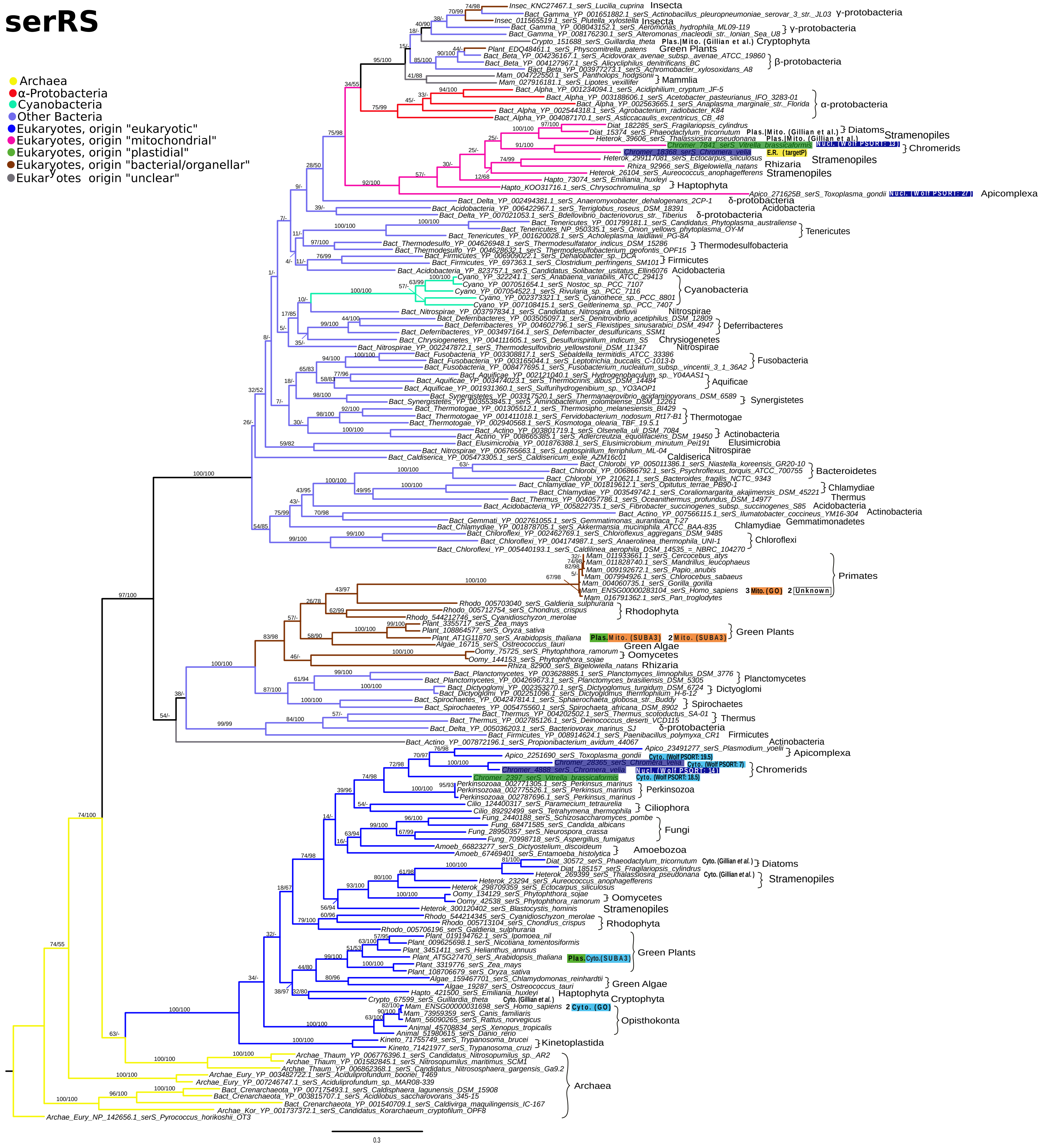
serRS
Archaea
α-Protobacteria
Cyanobacteria
Other Bacteria
Eukaryotes, origin "eukaryotic"
Eukaryotes, origin "mitochondrial"
Eukaryotes, origin "plastidial"
Eukaryotes, origin "bacterial/organellar"
Eukaryotes origin "unclear"
Insec_KNC27467.1_serS_Lucilia_cuprina
Insecta
Bact_Gamma_YP_001651882.1_serS_Actinobacillus_pleuropneumoniae_serovar_3_str._JL03
γ-protobacteria
Insec_011565519.1_serS_Plutella_xylostella
Insecta
Bact_Gamma_YP_008043152.1_serS_Aeromonas_hydrophila_ML09-119
Bact_Gamma_YP_008176230.1_serS_Alteromonas_macleodii_str._Ionian_Sea_U8
γ-protobacteria
Crypto_151688_serS_Guillardia_theta
Plas.|Mito. (Gillian et al.)
Cryptophyta
Plant_EDQ48461.1_serS_Physcomitrella_patens
Green Plants
Bact_Beta_YP_004236167.1_serS_Acidovorax_avenae_subsp._avenae_ATCC_19860
Bact_Beta_YP_004127967.1_serS_Alicycliphilus_denitrificans_BC
Bact_Beta_YP_003977273.1_serS_Achromobacter_xylosoxidans_A8
β-protobacteria
Mam_004722550.1_serS_Pantholops_hodgsonii
Mam_027916181.1_serS_Lipotes_vexillifer
Mammlia
Bact_Alpha_YP_001234094.1_serS_Acidiphilium_cryptum_JF-5
Bact_Alpha_YP_003188606.1_serS_Acetobacter_pasteurianus_IFO_3283-01
Bact_Alpha_YP_002563665.1_serS_Anaplasma_marginale_str._Florida
Bact_Alpha_YP_002544318.1_serS_Agrobacterium_radiobacter_K84
Bact_Alpha_YP_004087170.1_serS_Asticcacaulis_excentricus_CB_48
α-protobacteria
Diat_182285_serS_Fragilariopsis_cylindrus
Diat_15374_serS_Phaeodactylum_tricornutum
Plas.|Mito. (Gillian et al.)
Diatoms
Heterok_39606_serS_Thalassiosira_pseudonana
Plas.|Mito. (Gillian et al.)
Stramenopiles
Chromer_7841_serS_Vitrella_brassicaformis
Nucl. (Wolf PSORT: 13)
Chromer_18368_serS_Chromera_velia
E.R. (targetP)
Chromerids
Heterok_299117081_serS_Ectocarpus_siliculosus
Stramenopiles
Rhiza_92966_serS_Bigelowiella_natans
Rhizaria
Heterok_26104_serS_Aureococcus_anophagefferens
Stramenopiles
Hapto_73074_serS_Emiliania_huxleyi
Hapto_KOO31716.1_serS_Chrysochromulina_sp
Haptophyta
Apico_271625B_serS_Toxoplasma_gondii
Nucl. (Wolf PSORT: 27)
Apicomplexa
Bact_Delta_YP_002494381.1_serS_Anaeromyxobacter_dehalogenans_2CP-1
δ-protobacteria
Bact_Acidobacteria_YP_006422967.1_serS_Terriglobus_roseus_DSM_18391
Acidobacteria
Bact_Delta_YP_007021053.1_serS_Bdellovibrio_bacteriovorus_str._Tiberius
δ-protobacteria
Bact_Tenericutes_YP_001799181.1_serS_Candidatus_Phytoplasma_australiense
Bact_Tenericutes_NP_950335.1_serS_Onion_yellows_phytoplasma_OY-M
Bact_Tenericutes_YP_001620028.1_serS_Acholeplasma_laidlawii_PG-8A
Tenericutes
Bact_Thermodesulfo_YP_004626948.1_serS_Thermodesulfatator_indicus_DSM_15286
Bact_Thermodesulfo_YP_004628632.1_serS_Thermodesulfobacterium_geofontis_OPF15
Thermodesulfobacteria
Bact_Firmicutes_YP_006909022.1_serS_Dehalobacter_sp._DCA
Bact_Firmicutes_YP_697363.1_serS_Clostridium_perfringens_SM101
Firmicutes
Bact_Acidobacteria_YP_823757.1_serS_Candidatus_Solibacter_usitatus_Ellin6076
Acidobacteria
Cyano_YP_322241.1_serS_Anabaena_variabilis_ATCC_29413
Cyano_YP_007051654.1_serS_Nostoc_sp._PCC_7107
Cyano_YP_007054522.1_serS_Rivularia_sp._PCC_7116
Cyano_YP_002373321.1_serS_Cyanothece_sp._PCC_8801
Cyano_YP_007108415.1_serS_Geitlerinema_sp._PCC_7407
Cyanobacteria
Bact_Nitrospirae_YP_003797834.1_serS_Candidatus_Nitrospira_defluvii
Nitrospirae
Bact_Deferribacteres_YP_003505097.1_serS_Denitrovibrio_acetiphilus_DSM_12809
Bact_Deferribacteres_YP_004602796.1_serS_Flexistipes_sinusarabici_DSM_4947
Bact_Deferribacteres_YP_003497164.1_serS_Deferribacter_desulfuricans_SSM1
Deferribacteres
Bact_Chrysiogenetes_YP_004111605.1_serS_Desulfurispirillum_indicum_S5
Chrysiogenetes
Bact_Nitrospirae_YP_002247872.1_serS_Thermodesulfovibrio_yellowstonii_DSM_11347
Nitrospirae
Bact_Fusobacteria_YP_003308817.1_serS_Sebaldella_termitidis_ATCC_33386
Bact_Fusobacteria_YP_003165044.1_serS_Leptotrichia_buccalis_C-1013-b
Bact_Fusobacteria_YP_008477695.1_serS_Fusobacterium_nucleatum_subsp._vincentii_3_1_36A2
Fusobacteria
Bact_Aquificae_YP_002121040.1_serS_Hydrogenobaculum_sp._Y04AAS1
Bact_Aquificae_YP_003474023.1_serS_Thermocrinis_albus_DSM_14484
Bact_Aquificae_YP_001931360.1_serS_Sulfurihydrogenibium_sp._YO3AOP1
Aquificae
Bact_Synergistetes_YP_003317520.1_serS_Thermanaerovibrio_acidaminovorans_DSM_6589
Bact_Synergistetes_YP_003553845.1_serS_Aminobacterium_colombiense_DSM_12261
Synergistetes
Bact_Thermotogae_YP_001305512.1_serS_Thermosipho_melanesiensis_BI429
Bact_Thermotogae_YP_001411018.1_serS_Fervidobacterium_nodosum_Rt17-B1
Bact_Thermotogae_YP_002940568.1_serS_Kosmotoga_olearia_TBF_19.5.1
Thermotogae
Bact_Actino_YP_003801719.1_serS_Olsenella_uli_DSM_7084
Bact_Actino_YP_008665385.1_serS_Adlercreutzia_equolifaciens_DSM_19450
Actinobacteria
Bact_Elusimicrobia_YP_001876388.1_serS_Elusimicrobium_minutum_Pei191
Elusimicrobia
Bact_Nitrospirae_YP_006765663.1_serS_Leptospirillum_ferriphilum_ML-04
Nitrospirae
Bact_Caldiserica_YP_005473305.1_serS_Caldisericum_exile_AZM16c01
Caldiserica
Bact_Chlorobi_YP_005011386.1_serS_Niastella_koreensis_GR20-10
Bact_Chlorobi_YP_006866792.1_serS_Psychroflexus_torquis_ATCC_700755
Bact_Chlorobi_YP_210621.1_serS_Bacteroides_fragilis_NCTC_9343
Bacteroidetes
Bact_Chlamydiae_YP_001819612.1_serS_Opitutus_terrae_PB90-1
Bact_Chlamydiae_YP_003549742.1_serS_Coraliomargarita_akajimensis_DSM_45221
Chlamydiae
Bact_Thermus_YP_004057786.1_serS_Oceanithermus_profundus_DSM_14977
Thermus
Bact_Acidobacteria_YP_005822735.1_serS_Fibrobacter_succinogenes_subsp._succinogenes_S85
Acidobacteria
Bact_Actino_YP_007566115.1_serS_Ilumatobacter_coccineus_YM16-304
Actinobacteria
Bact_Gemmati_YP_002761055.1_serS_Gemmatimonas_aurantiaca_T-27
Gemmatimonadetes
Bact_Chlamydiae_YP_001878705.1_serS_Akkermansia_muciniphila_ATCC_BAA-835
Chlamydiae
Bact_Chloroflexi_YP_002462769.1_serS_Chloroflexus_aggregans_DSM_9485
Bact_Chloroflexi_YP_004174987.1_serS_Anaerolinea_thermophila_UNI-1
Bact_Chloroflexi_YP_005440193.1_serS_Caldilinea_aerophila_DSM_14535_=_NBRC_104270
Chloroflexi
Mam_011933661.1_serS_Cercocebus_atys
Mam_011828740.1_serS_Mandrillus_leucophaeus
Mam_009192672.1_serS_Papio_anubis
Mam_007994926.1_serS_Chlorocebus_sabaeus
Mam_004060735.1_serS_Gorilla_gorilla
Mam_ENSG00000283104_serS_Homo_sapiens
3 Mito. (GO) 2 Unknown
Mam_016791362.1_serS_Pan_troglodytes
Primates
Rhodo_005703040_serS_Galdieria_sulphuraria
Rhodo_005712754_serS_Chondrus_crispus
Rhodo_544212746_serS_Cyanidioschyzon_merolae
Rhodophyta
Plant_3355717_serS_Zea_mays
Plant_108864577_serS_Oryza_sativa
Plant_AT1G11870_serS_Arabidopsis_thaliana
Plas. Mito. (SUBA3) 2 Mito. (SUBA3)
Green Plants
Algae_16715_serS_Ostreococcus_tauri
Green Algae
Oomy_75725_serS_Phytophthora_ramorum
Oomy_144153_serS_Phytophthora_sojae
Oomycetes
Rhiza_82900_serS_Bigelowiella_natans
Rhizaria
Bact_Planctomycetes_YP_003628885.1_serS_Planctomyces_limnophilus_DSM_3776
Bact_Planctomycetes_YP_004269673.1_serS_Planctomyces_brasiliensis_DSM_5305
Planctomycetes
Bact_Dictyoglomi_YP_002353270.1_serS_Dictyoglomus_turgidum_DSM_6724
Bact_Dictyoglomi_YP_002251096.1_serS_Dictyoglomus_thermophilum_H-6-12
Dictyoglomi
Bact_Spirochaetes_YP_004247814.1_serS_Sphaerochaeta_globosa_str._Buddy
Bact_Spirochaetes_YP_005475560.1_serS_Spirochaeta_africana_DSM_8902
Spirochaetes
Bact_Thermus_YP_004202502.1_serS_Thermus_scotoductus_SA-01
Bact_Thermus_YP_002785126.1_serS_Deinococcus_deserti_VCD115
Thermus
Bact_Delta_YP_005036203.1_serS_Bacteriovorax_marinus_SJ
δ-protobacteria
Bact_Firmicutes_YP_008914624.1_serS_Paenibacillus_polymyxa_CR1
Firmicutes
Bact_Actino_YP_007872196.1_serS_Propionibacterium_avidum_44067
Actinobacteria
Apico_23491277_serS_Plasmodium_yoelii
Apico_2251690_serS_Toxoplasma_gondii
Cyto. (Wolf PSORT: 19.5)
Apicomplexa
Chromer_28365_serS_Chromera_velia
Cyto. (Wolf PSORT: 7)
Chromer_4888_serS_Chromera_velia
Nucl. (Wolf PSORT: 14)
Chromer_2397_serS_Vitrella_brassicaformis
Cyto. (Wolf PSORT: 18.5)
Chromerids
Perkinsozoaa_002771305.1_serS_Perkinsus_marinus
Perkinsozoaa_002775526.1_serS_Perkinsus_marinus
Perkinsozoaa_002787696.1_serS_Perkinsus_marinus
Perkinsozoa
Cilio_124400317_serS_Paramecium_tetraurelia
Cilio_89292499_serS_Tetrahymena_thermophila
Ciliophora
Fung_2440188_serS_Schizosaccharomyces_pombe
Fung_68471585_serS_Candida_albicans
Fung_28950357_serS_Neurospora_crassa
Fung_70998718_serS_Aspergillus_fumigatus
Fungi
Amoeb_66823277_serS_Dictyostelium_discoideum
Amoeb_67469401_serS_Entamoeba_histolytica
Amoebozoa
Diat_30572_serS_Phaeodactylum_tricornutum
Cyto. (Gillian et al.)
Diat_185157_serS_Fragilariopsis_cylindrus
Diatoms
Heterok_269399_serS_Thalassiosira_pseudonana
Cyto. (Gillian et al.)
Heterok_23294_serS_Aureococcus_anophagefferens
Heterok_298709359_serS_Ectocarpus_siliculosus
Stramenopiles
Oomy_134129_serS_Phytophthora_sojae
Oomy_42538_serS_Phytophthora_ramorum
Oomycetes
Heterok_300120402_serS_Blastocystis_hominis
Stramenopiles
Rhodo_544214345_serS_Cyanidioschyzon_merolae
Rhodo_005713104_serS_Chondrus_crispus
Rhodo_005706196_serS_Galdieria_sulphuraria
Rhodophyta
Plant_019194762.1_serS_Ipomoea_nil
Plant_009625698.1_serS_Nicotiana_tomentosiformis
Plant_3451411_serS_Helianthus_annuus
Plant_AT5G27470_serS_Arabidopsis_thaliana
Plas. Cyto. (SUBA3)
Plant_3319776_serS_Zea_mays
Plant_108706679_serS_Oryza_sativa
Green Plants
Algae_159467701_serS_Chlamydomonas_reinhardtii
Algae_19287_serS_Ostreococcus_tauri
Green Algae
Hapto_421500_serS_Emiliania_huxleyi
Haptophyta
Crypto_67599_serS_Guillardia_theta
Cyto. (Gillian et al.)
Cryptophyta
Mam_ENSG00000031698_serS_Homo_sapiens
2 Cyto. (GO)
Mam_73959359_serS_Canis_familiaris
Mam_56090265_serS_Rattus_norvegicus
Animal_45708834_serS_Xenopus_tropicalis
Animal_51980615_serS_Danio_rerio
Opisthokonta
Kineto_71755749_serS_Trypanosoma_brucei
Kineto_71421977_serS_Trypanosoma_cruzi
Kinetoplastida
Archae_Thaum_YP_006776396.1_serS_Candidatus_Nitrososphaera_sp._AR2
Archae_Thaum_YP_001582845.1_serS_Nitrosopumilus_maritimus_SCM1
Archae_Thaum_YP_006862368.1_serS_Candidatus_Nitrososphaera_gargensis_Ga9.2
Archae_Eury_YP_003482722.1_serS_Aciduliprofundum_boonei_T469
Archae_Eury_YP_007246747.1_serS_Aciduliprofundum_sp._MAR08-339
Bact_Crenarchaeota_YP_007175493.1_serS_Caldisphaera_lagunensis_DSM_15908
Bact_Crenarchaeota_YP_003815707.1_serS_Acidilobus_saccharovorans_345-15
Bact_Crenarchaeota_YP_001540709.1_serS_Caldivirga_maquilingensis_IC-167
Archae_Kor_YP_001737372.1_serS_Candidatus_Korarchaeum_cryptofilum_OPF8
Archaea
Archae_Eury_NP_142656.1_serS_Pyrococcus_horikoshii_OT3
0.3

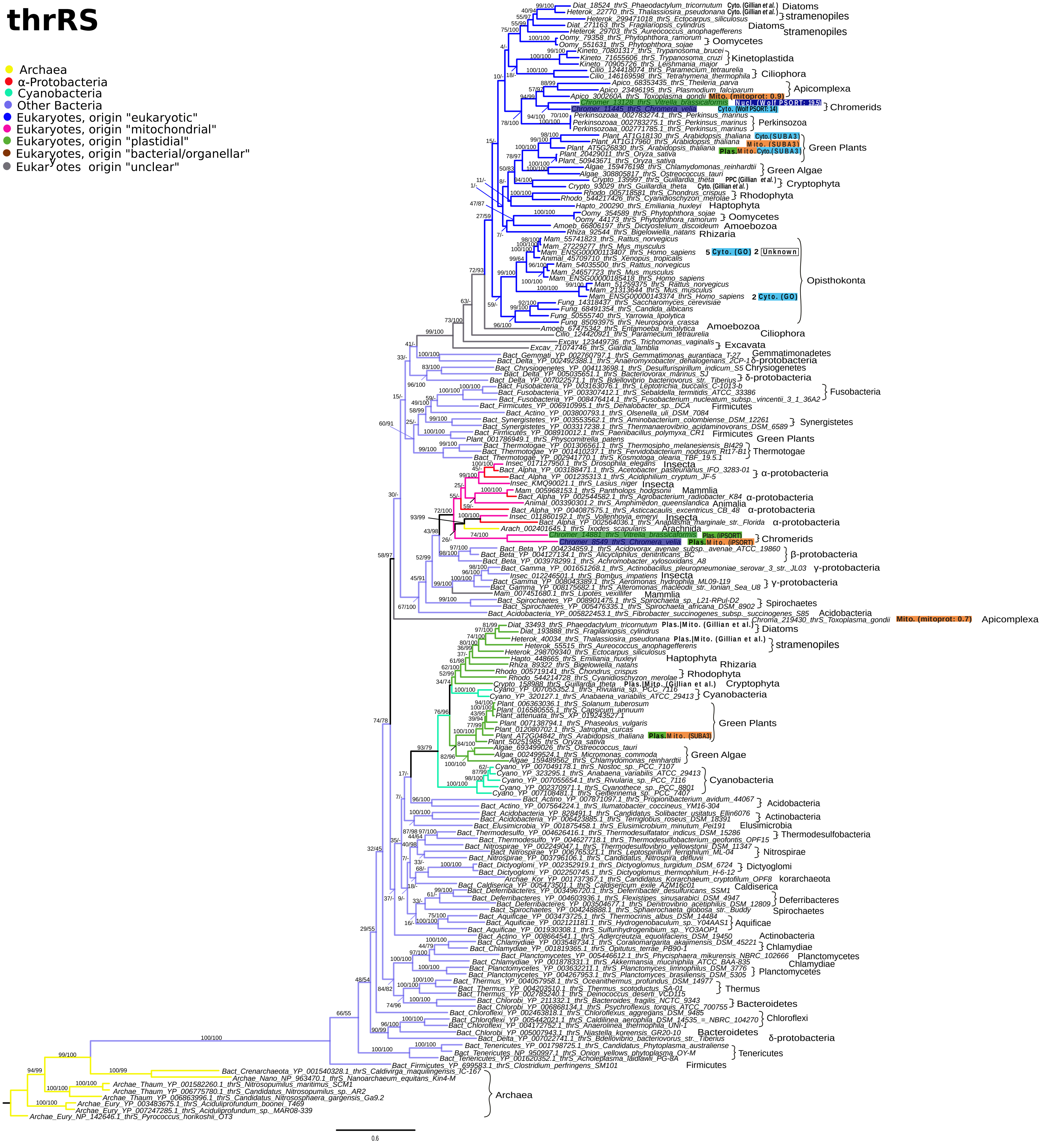
thrRS
Archaea
α-Protobacteria
Cyanobacteria
Other Bacteria
Eukaryotes, origin "eukaryotic"
Eukaryotes, origin "mitochondrial"
Eukaryotes, origin "plastidial"
Eukaryotes, origin "bacterial/organellar"
Eukaryotes origin "unclear"
Diat_18524_thrS_Phaeodactylum_tricornutum Cyto. (Gillian et al.)
Heterok_22770_thrS_Thalassiosira_pseudonana Cyto. (Gillian et al.)
Diatoms
Heterok_299471018_thrS_Ectocarpus_siliculosus
stramenopiles
Diat_271163_thrS_Fragilariopsis_cylindrus
Diatoms
Heterok_29703_thrS_Aureococcus_anophagefferens
stramenopiles
Oomy_79358_thrS_Phytophthora_ramorum
Oomy_551631_thrS_Phytophthora_sojae
Oomycetes
Kineto_70801317_thrS_Trypanosoma_brucei
Kineto_71655606_thrS_Trypanosoma_cruzi
Kineto_70905726_thrS_Leishmania_major
Kinetoplastida
Cilio_124418074_thrS_Paramecium_tetraurelia
Cilio_146169598_thrS_Tetrahymena_thermophila
Ciliophora
Apico_68353435_thrS_Theileria_parva
Apico_23496195_thrS_Plasmodium_falciparum
Apico_300260A_thrS_Toxoplasma_gondii Mito. (mitoprot: 0.9)
Apicomplexa
Chromer_13128_thrS_Vitrella_brassicaformis Nucl. (Wolf PSORT: 19.5)
Chromer_11445_thrS_Chromera_velia Cyto. (Wolf PSORT: 14)
Chromerids
Perkinsozoaa_002783274.1_thrS_Perkinsus_marinus
Perkinsozoaa_002783275.1_thrS_Perkinsus_marinus
Perkinsozoaa_002771785.1_thrS_Perkinsus_marinus
Perkinsozoa
Plant_AT1G18130_thrS_Arabidopsis_thaliana Cyto. (SUBA3)
Plant_AT1G17960_thrS_Arabidopsis_thaliana Mito. (SUBA3)
Plant_AT5G26830_thrS_Arabidopsis_thaliana Plas. Mito. Cyto. (SUBA3)
Plant_20429011_thrS_Oryza_sativa
Plant_50943671_thrS_Oryza_sativa
Green Plants
Algae_159476198_thrS_Chlamydomonas_reinhardtii
Algae_308805817_thrS_Ostreococcus_tauri
Green Algae
Crypto_139997_thrS_Guillardia_theta PPC (Gillian et al.)
Crypto_93029_thrS_Guillardia_theta Cyto. (Gillian et al.)
Cryptophyta
Rhodo_005718581_thrS_Chondrus_crispus
Rhodo_544217426_thrS_Cyanidioschyzon_merolae
Rhodophyta
Hapto_200290_thrS_Emiliania_huxleyi
Haptophyta
Oomy_354589_thrS_Phytophthora_sojae
Oomy_44173_thrS_Phytophthora_ramorum
Oomycetes
Amoeb_66806197_thrS_Dictyostelium_discoideum
Amoebozoa
Rhiza_92544_thrS_Bigelowiella_natans
Rhizaria
Mam_55741823_thrS_Rattus_norvegicus
Mam_27229277_thrS_Mus_musculus
Mam_ENSG00000113407_thrS_Homo_sapiens 5 Cyto. (GO) 2 Unknown
Animal_45709710_thrS_Xenopus_tropicalis
Mam_54035500_thrS_Rattus_norvegicus
Mam_24657723_thrS_Mus_musculus
Mam_ENSG00000185418_thrS_Homo_sapiens
Mam_51259375_thrS_Rattus_norvegicus
Mam_21313644_thrS_Mus_musculus
Mam_ENSG00000143374_thrS_Homo_sapiens 2 Cyto. (GO)
Fung_14318437_thrS_Saccharomyces_cerevisiae
Fung_68491354_thrS_Candida_albicans
Fung_50555740_thrS_Yarrowia_lipolytica
Fung_85093975_thrS_Neurospora_crassa
Opisthokonta
Amoeb_67475342_thrS_Entamoeba_histolytica
Amoebozoa
Cilio_124420921_thrS_Paramecium_tetraurelia
Ciliophora
Excav_123449736_thrS_Trichomonas_vaginalis
Excav_71074746_thrS_Giardia_lamblia
Excavata
Bact_Gemmati_YP_002760797.1_thrS_Gemmatimonas_aurantiaca_T-27
Gemmatimonadetes
Bact_Delta_YP_002492388.1_thrS_Anaeromyxobacter_dehalogenans_2CP-1
δ-protobacteria
Bact_Chrysiogenetes_YP_004113698.1_thrS_Desulfurispirillum_indicum_S5
Chrysiogenetes
Bact_Delta_YP_005035651.1_thrS_Bacteriovorax_marinus_SJ
Bact_Delta_YP_007022571.1_thrS_Bdellovibrio_bacteriovorus_str._Tiberius
δ-protobacteria
Bact_Fusobacteria_YP_003163076.1_thrS_Leptotrichia_buccalis_C-1013-b
Bact_Fusobacteria_YP_003307412.1_thrS_Sebaldella_termitidis_ATCC_33386
Bact_Fusobacteria_YP_008476414.1_thrS_Fusobacterium_nucleatum_subsp._vincentii_3_1_36A2
Fusobacteria
Bact_Firmicutes_YP_006910995.1_thrS_Dehalobacter_sp._DCA
Firmicutes
Bact_Actino_YP_003800793.1_thrS_Olsenella_uli_DSM_7084
Bact_Synergistetes_YP_003553562.1_thrS_Aminobacterium_colombiense_DSM_12261
Bact_Synergistetes_YP_003317238.1_thrS_Thermanaerovibrio_acidaminovorans_DSM_6589
Synergistetes
Bact_Firmicutes_YP_008910012.1_thrS_Paenibacillus_polymyxa_CR1
Firmicutes
Plant_001786949.1_thrS_Physcomitrella_patens
Green Plants
Bact_Thermotogae_YP_001306561.1_thrS_Thermosipho_melanesiensis_BI429
Bact_Thermotogae_YP_001410237.1_thrS_Fervidobacterium_nodosum_Rt17-B1
Bact_Thermotogae_YP_002941770.1_thrS_Kosmotoga_olearia_TBF_19.5.1
Thermotogae
Insec_017127950.1_thrS_Drosophila_elegans
Insecta
Bact_Alpha_YP_003188471.1_thrS_Acetobacter_pasteurianus_IFO_3283-01
Bact_Alpha_YP_001235313.1_thrS_Acidiphilium_cryptum_JF-5
α-protobacteria
Insec_KMQ90021.1_thrS_Lasius_niger
Insecta
Mam_005968153.1_thrS_Pantholops_hodgsonii
Mammlia
Bact_Alpha_YP_002544582.1_thrS_Agrobacterium_radiobacter_K84
α-protobacteria
Animal_003390301.2_thrS_Amphimedon_queenslandica
Animalia
Bact_Alpha_YP_004087575.1_thrS_Asticcacaulis_excentricus_CB_48
α-protobacteria
Insec_011860192.1_thrS_Vollenhovia_emeryi
Insecta
Bact_Alpha_YP_002564036.1_thrS_Anaplasma_marginale_str._Florida
α-protobacteria
Arach_002401645.1_thrS_Ixodes_scapularis
Arachnida
Chromer_14881_thrS_Vitrella_brassicaformis Plas. (iPSORT)
Chromer_8549_thrS_Chromera_velia Plas. Mito. (iPSORT)
Chromerids
Bact_Beta_YP_004234859.1_thrS_Acidovorax_avenae_subsp._avenae_ATCC_19860
Bact_Beta_YP_004127134.1_thrS_Alicycliphilus_denitrificans_BC
Bact_Beta_YP_003978299.1_thrS_Achromobacter_xylosoxidans_A8
β-protobacteria
Bact_Gamma_YP_001651268.1_thrS_Actinobacillus_pleuropneumoniae_serovar_3_str._JL03
γ-protobacteria
Insec_012246501.1_thrS_Bombus_impatiens
Insecta
Bact_Gamma_YP_008043389.1_thrS_Aeromonas_hydrophila_ML09-119
Bact_Gamma_YP_008175682.1_thrS_Alteromonas_macleodii_str._Ionian_Sea_U8
γ-protobacteria
Mam_007451680.1_thrS_Lipotes_vexillifer
Mammlia
Bact_Spirochaetes_YP_008901475.1_thrS_Spirochaeta_sp._L21-RPul-D2
Bact_Spirochaetes_YP_005476335.1_thrS_Spirochaeta_africana_DSM_8902
Spirochaetes
Bact_Acidobacteria_YP_005822453.1_thrS_Fibrobacter_succinogenes_subsp._succinogenes_S85
Acidobacteria
Chroma_219430_thrS_Toxoplasma_gondii Mito. (mitoprot: 0.7)
Apicomplexa
Diat_33493_thrS_Phaeodactylum_tricornutum Plas.|Mito. (Gillian et al.)
Diat_193888_thrS_Fragilariopsis_cylindrus
Diatoms
Heterok_40034_thrS_Thalassiosira_pseudonana Plas.|Mito. (Gillian et al.)
Heterok_55515_thrS_Aureococcus_anophagefferens
Heterok_298709340_thrS_Ectocarpus_siliculosus
stramenopiles
Hapto_448665_thrS_Emiliania_huxleyi
Haptophyta
Rhiza_89322_thrS_Bigelowiella_natans
Rhizaria
Rhodo_005719141_thrS_Chondrus_crispus
Rhodo_544214728_thrS_Cyanidioschyzon_merolae
Rhodophyta
Crypto_158988_thrS_Guillardia_theta Plas.|Mito. (Gillian et al.)
Cryptophyta
Cyano_YP_007055352.1_thrS_Rivularia_sp._PCC_7116
Cyano_YP_320127.1_thrS_Anabaena_variabilis_ATCC_29413
Cyanobacteria
Plant_006363036.1_thrS_Solanum_tuberosum
Plant_016580555.1_thrS_Capsicum_annuum
Plant_attenuata_thrS_XP_019243527.1
Plant_007138794.1_thrS_Phaseolus_vulgaris
Plant_012080702.1_thrS_Jatropha_curcas
Plant_AT2G04842_thrS_Arabidopsis_thaliana Plas. Mito. (SUBA3)
Plant_50251985_thrS_Oryza_sativa
Green Plants
Algae_693499026_thrS_Ostreococcus_tauri
Algae_002499524.1_thrS_Micromonas_commoda
Algae_159489562_thrS_Chlamydomonas_reinhardtii
Green Algae
Cyano_YP_007049178.1_thrS_Nostoc_sp._PCC_7107
Cyano_YP_323295.1_thrS_Anabaena_variabilis_ATCC_29413
Cyano_YP_007055654.1_thrS_Rivularia_sp._PCC_7116
Cyano_YP_002370971.1_thrS_Cyanothece_sp._PCC_8801
Cyano_YP_007108481.1_thrS_Geitlerinema_sp._PCC_7407
Cyanobacteria
Bact_Actino_YP_007871097.1_thrS_Propionibacterium_avidum_44067
Acidobacteria
Bact_Actino_YP_007564224.1_thrS_Ilumatobacter_coccineus_YM16-304
Bact_Acidobacteria_YP_828491.1_thrS_Candidatus_Solibacter_usitatus_Ellin6076
Bact_Acidobacteria_YP_006423885.1_thrS_Terriglobus_roseus_DSM_18391
Actinobacteria
Bact_Elusimicrobia_YP_001875458.1_thrS_Elusimicrobium_minutum_Pei191
Elusimicrobia
Bact_Thermodesulfo_YP_004626416.1_thrS_Thermodesulfatator_indicus_DSM_15286
Bact_Thermodesulfo_YP_004627718.1_thrS_Thermodesulfobacterium_geofontis_OPF15
Thermodesulfobacteria
Bact_Nitrospirae_YP_002249047.1_thrS_Thermodesulfovibrio_yellowstonii_DSM_11347
Bact_Nitrospirae_YP_006765321.1_thrS_Leptospirillum_ferriphilum_ML-04
Bact_Nitrospirae_YP_003796106.1_thrS_Candidatus_Nitrospira_defluvii
Nitrospirae
Bact_Dictyoglomi_YP_002352919.1_thrS_Dictyoglomus_turgidum_DSM_6724
Bact_Dictyoglomi_YP_002250745.1_thrS_Dictyoglomus_thermophilum_H-6-12
Dictyoglomi
Archae_Kor_YP_001737367.1_thrS_Candidatus_Korarchaeum_cryptofilum_OPF8
korarchaeota
Bact_Caldiserica_YP_005473501.1_thrS_Caldisericum_exile_AZM16c01
Caldiserica
Bact_Deferribacteres_YP_003496720.1_thrS_Deferribacter_desulfuricans_SSM1
Bact_Deferribacteres_YP_004603936.1_thrS_Flexistipes_sinusarabici_DSM_4947
Bact_Deferribacteres_YP_003504677.1_thrS_Denitrovibrio_acetiphilus_DSM_12809
Deferribacteres
Bact_Spirochaetes_YP_004248888.1_thrS_Sphaerochaeta_globosa_str._Buddy
Spirochaetes
Bact_Aquificae_YP_003473725.1_thrS_Thermocrinis_albus_DSM_14484
Bact_Aquificae_YP_002121181.1_thrS_Hydrogenobaculum_sp._Y04AAS1
Bact_Aquificae_YP_001930308.1_thrS_Sulfurihydrogenibium_sp._YO3AOP1
Aquificae
Bact_Actino_YP_008664541.1_thrS_Adlercreutzia_equolifaciens_DSM_19450
Actinobacteria
Bact_Chlamydiae_YP_003548734.1_thrS_Coraliomargarita_akajimensis_DSM_45221
Bact_Chlamydiae_YP_001819365.1_thrS_Opitutus_terrae_PB90-1
Chlamydiae
Bact_Planctomycetes_YP_005446612.1_thrS_Phycisphaera_mikurensis_NBRC_102666
Planctomycetes
Bact_Chlamydiae_YP_001878331.1_thrS_Akkermansia_muciniphila_ATCC_BAA-835
Chlamydiae
Bact_Planctomycetes_YP_003632211.1_thrS_Planctomyces_limnophilus_DSM_3776
Bact_Planctomycetes_YP_004267953.1_thrS_Planctomyces_brasiliensis_DSM_5305
Planctomycetes
Bact_Thermus_YP_004057958.1_thrS_Oceanithermus_profundus_DSM_14977
Bact_Thermus_YP_004203510.1_thrS_Thermus_scotoductus_SA-01
Bact_Thermus_YP_002785240.1_thrS_Deinococcus_deserti_VCD115
Thermus
Bact_Chlorobi_YP_211332.1_thrS_Bacteroides_fragilis_NCTC_9343
Bact_Chlorobi_YP_006868134.1_thrS_Psychroflexus_torquis_ATCC_700755
Bacteroidetes
Bact_Chloroflexi_YP_002463818.1_thrS_Chloroflexus_aggregans_DSM_9485
Bact_Chloroflexi_YP_005442021.1_thrS_Caldilinea_aerophila_DSM_14535_=_NBRC_104270
Bact_Chloroflexi_YP_004172752.1_thrS_Anaerolinea_thermophila_UNI-1
Chloroflexi
Bact_Chlorobi_YP_005007943.1_thrS_Niastella_koreensis_GR20-10
Bacteroidetes
Bact_Delta_YP_007022741.1_thrS_Bdellovibrio_bacteriovorus_str._Tiberius
δ-protobacteria
Bact_Tenericutes_YP_001798725.1_thrS_Candidatus_Phytoplasma_australiense
Bact_Tenericutes_NP_950997.1_thrS_Onion_yellows_phytoplasma_OY-M
Bact_Tenericutes_YP_001620352.1_thrS_Acholeplasma_laidlawii_PG-8A
Tenericutes
Bact_Firmicutes_YP_699583.1_thrS_Clostridium_perfringens_SM101
Firmicutes
Bact_Crenarchaeota_YP_001540328.1_thrS_Caldivirga_maquilingensis_IC-167
Archae_Nano_NP_963470.1_thrS_Nanoarchaeum_equitans_Kin4-M
Archae_Thaum_YP_001582260.1_thrS_Nitrosopumilus_maritimus_SCM1
Archae_Thaum_YP_006775780.1_thrS_Candidatus_Nitrosopumilus_sp._AR2
Archae_Thaum_YP_006863996.1_thrS_Candidatus_Nitrososphaera_gargensis_Ga9.2
Archae_Eury_YP_003483675.1_thrS_Aciduliprofundum_boonei_T469
Archae_Eury_YP_007247285.1_thrS_Aciduliprofundum_sp._MAR08-339
Archae_Eury_NP_142646.1_thrS_Pyrococcus_horikoshii_OT3
Archaea
0.6
