# Supplementary material for: Characterization of Aminoacyl-tRNA Synthetases in Chromerids
Source: Genes (Basel). 2019 Jul 31;10(8):582. doi: 10.3390/genes10080582 (PMC6723311; doi:10.3390/genes10080582)
Supplement: Supplementary file 1 [file genes-10-00582-s001.zip › Supplementary Materials/Supplmentary file sf1.doc]

**Arginyl-tRNA synthetase (ArgRS)**

Three genes coding for ArgRSs were found in *C. velia* while in contrast to one gene in *V. brassicaformis*. *C. velia* ArgRS (cvel_22194) originates in a eukaryotic nucleus, because of the position in the clade composed of all eukaryotes, forming a sister group to apicomplexans *Toxoplasma gondii* and *Cryptosporidium hominis*. A second *C. velia* (cvel_7363) ArgRS displays a bacterial origin, forms a sister group to the rhizarian *Bigelowiella natans* and is closely related to δ-proteobacteria. The last *C. velia* sequence (cvel_3008) and the only ArgRS from *V. brassicaformis* (vbra_19912) cluster with cyanobacteria suggesting their origins in a plastid (Supplementary Figure 1).

The tree contains two groups of eukaryotic sequences: the first is related to Bacteria and Archaea (Nanoarchaeota), contains opisthokonts, amoebozoans, rhizarians, and chromerids. The second group of eukaryotic sequences constitutes the crown of the tree, with the γ-proteobacteria as the sister group, with the eukaryotic clade placed on relatively long branch (Figure 2).

**Cysteinyl-tRNA synthetase (CysRS)**

Both *C. velia* and *V. brassicaformis* possess two genes coding for CysRS: *V. brassicaformis* CysRS (vbra_7006) clusters with stramenopiles, the entire clade is closely related to Thermodesulfobacteria and δ-proteobacteria, pointing out their bacterial origins. The same applies to one sequence from *C. velia* (cvel_1412) (see Supplementary Figure 1). The rest CysRSs from the chromerids cluster with remaining eukaryotes likely suggesting their origins in a eukaryotic nucleus. *C. velia* (cvel_32019) and *V. brassicaformis* (vbra_2178) sequences form a sister group to kinetoplastids and the apicomplexan *Toxoplasma gondii*, respectively. Both *C. velia* and *V. brassicaformis* lack the cyanobacterial CysRS (Supplementary Figure 1).

The tree contains two groups of eukaryotic sequences: the first one, which is related to Archaea, contains chromerids branching together with haptophyte, cryptophytes, rhizarians, stramenopiles, diatoms, and rhodophytes; these genes are closely related to bacterial counterparts (Spirochaetes, Bacteroidetes, and Thermus) (Figure 2). The other eukaryotes are placed in the crown of the tree and clustered with various bacteria (Figure 2).

**Glutamyl-tRNA synthetase (GluRS)**

Both *C. velia* and *V. brassicaformis* genomes encode one gene for GluRS: *C. velia* (cvel_31597) and *V. brassicaformis* (vbra_21912) sequences cluster together with cryptophytes, stramenopiles, rhodophytes, plants and green algae as their closest relatives. These enzymes seem to be closely related to γ-proteobacteria (Supplementary Figure 1).

The tree contains two groups of eukaryotic sequences: the first one is related to Archaea (Figure 2). The other eukaryotes are placed on the crown of the bacterial clade, which was quite distinct from the eukaryotic clade (Figure 2).

**Glutaminyl-tRNA synthetase (GlnRS)**

Three various genes coding for GlnRSs were found in *C. velia*, while two genes were found in *V. brassicaformis*. *C. velia* uses paralogs of GlnRSs (cvel_8110; cvel_1112), which very likely originates in the duplication of the gene mitochondrial in origin, like apicomplexans. The same applies to one sequence from *V. brassicaformis* (vbra_11768) (Supplementary Figure 1). The remaining *C. velia* and *V. brassicaformis* sequences form together with green plants a sister group closely related to Archaea suggesting the origin in the eukaryotic nucleus. Both *C. velia* and *V. brassicaformis* lack the cyanobacterial GlnRS (Figure 2).

Eukaryotes appear in two different positions in the tree: the first group consists of chromerids and apicomplexans, and clusters with α-proteobacteria suggesting their origin in the mitochondrion. The second eukaryotic group includes green plants, chromerids, and stramenopiles, with Archaea sequences as a sister suggesting their origin in the eukaryotic nucleus; the last group of eukaryotic sequences (the species-richest one) appears on the crown of the tree, forming a sister to Archaea related eukaryotes (Supplementary Figure 1).

**Leucyl-tRNA synthetase (LeuRS)**

Both *C. velia* and *V. brassicaformis* codes for two LeuRS genes: sequences from *V. brassicaformis* (vbra_9824), and *C. velia* (cvel_16973) likely originate in a eukaryotic nucleus, branching in the clade composed of eukaryotes with Archaea as a sister group (Supplementary Figure 1). The other *C. velia* and *V. brassicaformis* sequences cluster with cryptophytes and apicomplexans closely related to δ-proteobacteria and Bacteroidetes, pointing out their probable bacterial origin. Surprisingly cyanobacterial LeuRSs appears very closely related to opisthokonts, oomycetes, and ciliates (*Tetrahymena thermophila*). The gene from the mite *Ixodes scapularis* branches inside α-proteobacteria (Supplementary Figure 1).

**Isoleucyl-tRNA synthetase (IleRS)**

Two clades containing eukaryotes are recognized in the tree. Both *C. velia* and *V. brassicaformis* possess genes coding for IleRS in both these clades. *C. velia* (cvel_28875), and *V. brassicaformis* (vbra_7770) very likely originate in a eukaryotic nucleus, because of their position in the clade composed of eukaryotes (excavates, amoebozoans, rhodophytes, rhizarians, stramenopiles, haptophytes, archaeplastids, alveolates) with chromerids forming a sister group to apicomplexans (Supplementary Figure 1). However, this clade is rooted by various bacteria, with Archaea as the earliest branching group. The other *C. velia* (cvel_28423) and *V. brassicaformis* (vbra_11931) sequences cluster in a separate clade with rhizarians, stramenopiles, diatoms, cryptophytes, rhodophytes and haptophytes in the clade related to the cyanobacterial-plastid sequences from plants (Figure 2). However, opisthokonts and oomycetes constitute the sister clade the cyanobacterial-plastid one (Supplementary Figure 1). Both above mentioned eukaryotic clusters are rooted by bacteria; the later eukaryotic clade appears to be related to the Planctomycetes (Figure 2).

**Methionyl-tRNA synthetase (MetRS)**

Both *C. velia* and *V. brassicaformis* possess two genes coding for MetRS. They branch within separated eukaryotic clades with an unclear (unsupported) relationship. Chromerid MetRS (cvel_21221; vbra_13101) either cluster with rhizarians, stramenopiles, diatoms, cryptophytes and haptophytes ; other chromerid genes (cvel_7165; vbra_1113) constitute the clade with stramenopiles (including oomycetes), haptophytes, cryptophytes, perkinsids, chlorophytes, and apicomplexans. The positions of mentioned clades are not supported, but clades themselves are supported well (Figure 2).

The genes from chromerids (cvel_21221; vbra_13101) cluster together with alphaproteobacteria and likely represent MetRS of mitochondrial origins. This mitochondrial clade forms the sister group to the two eukaryotic subclades. Like AlaRS, there is another clade of eukaryotic sequences from ciliates, rhodophytes, green plants and algae, and animals (again exclusively primates), which appears closely related to Archaea (Supplementary Figure 1).

**Tryptophanyl-tRNA synthetase (TrpRS)**

Two various genes coding for TrpRSs were found in *V. brassicaformis* and *C. velia*: one gene (cvel_7641 and vbra_16713) originates in a eukaryotic nucleus, forming a sister group to perkinsids (Supplementary Figure 1). Two chromerid genes (cvel_20758 and vbra_20646) nicely show a cyanobacterial origin. The mitochondrial TrpRS was identified only in ciliates (Supplementary Figure 1).

The tree contains three groups of eukaryotic sequences: the first one is related to Archaea, contains chromerids branching together with perkinsids, haptophytes, cryptophytes, chlorophytes and apicomplexans (Figure 2). The second eukaryotic group includes chromerids, stramenopiles, green algae, plants, rhodophytes, haptophytes, rhizarians, and cryptophytes, which cluster together with cyanobacterial sequences. Other eukaryotes, such as opisthokonts and oomycetes, constitute the clade closely related to alphaproteobacteria (Figure 2).

**Tyrosyl-tRNA synthetase (TyrRS)**

Both *C. velia* and *V. brassicaformis* possess two genes coding for TyrRS: one gene from *V. brassicaformis* TyrRS (vbra_13490) and *C. velia* (cvel_4070) likely originates in a eukaryotic nucleus, because of the position in the clade composed of all eukaryotes rooted by Archaea (Supplementary Figure 1). The second *C. velia* and *V. brassicaformis* sequences (cvel_7146; vbra_8727) cluster with rhizarians, stramenopiles, diatoms, cryptophytes and haptophytes in a clade, closely related to alphaproteobacteria suggesting its possible mitochondrial origin (Supplementary Figure 1). Both *C. velia* and *V. brassicaformis* lack the cyanobacterial TyrRS (Figure 2).

The tree contains four groups of eukaryotic sequences: the first one is related to Archaea, the second is placed on the crown of the tree with δ-proteobacteria and Actinobacteria on the root of the clade. The third one, likely mitochondrial, contains rhizarians, stramenopiles, diatoms, cryptophytes, haptophytes and chromerids (Figure 2). The last group includes green algae, plants, and opisthokonts, and forms a paraphyletic sister group placed on a relatively long branch (Figure 2).

**Valyl-tRNA synthetase (ValRS)**

Three genes coding for ValRSs were found in *C. velia*, while two genes codes for it in *V. brassicaformis*. *C. velia* uses duplicated ValRSs (cvel_6889; cvel_16754), which likely originate in a eukaryotic nucleus, because of the position in the clade composed exclusively of eukaryotes. The same applies to the sequence from *V. brassicaformis* (vbra_19209) (Supplementary Figure 1). The remaining ValRSs from chromerids (vbra_14431 and cvel_9091) form a sister group to rhizarians and stramenopiles, closely related to Chloroflexi. Both *C. velia* and *V. brassicaformis* lack the cyanobacterial ValRS (Supplementary Figure 1).

The tree contains two groups of eukaryotic sequences: the first one is placed on the crown of the tree composed from most of the eukaryotes (Figure 2). The second eukaryotic group containing only phototrophs is distinct from the crown eukaryotic clade and is related to Chloroflexi (Supplementary Figure 1).

**Alanyl-tRNA synthetase (AlaRS)**

Three genes coding for AlaRS were found in *C. velia* in contrast to two genes in *V. brassicaformis*. *C. velia* uses duplicated AlaRSs (cvel_21018; cvel_19371), which likely originates in a eukaryotic nucleus, because of their position in the clade composed of eukaryotes. The same applies for one sequence from *V. brassicaformis* (vbra_6631) branching as a sister to the apicomplexan *Toxoplasma gondii*. The other *C. velia* and *V. brassicaformis* sequences (cvel_11640 and vbra_13386) cluster with Betaproteobacteria and Gammaproteobacteria suggesting their bacterial origin. Both *C. velia* and *V. brassicaformis* lack the cyanobacterial AlaRS (Supplementary Figure 1).

The tree contains two groups of eukaryotic sequences: the first one is related to Archaea and contains excavates, ciliates, perkinsids, green algae, plants, and animals (exclusively primates). The second group is placed on the crown of the tree, with the Planctomycetes, Chlamydiae, and Bacteroidetes on the root (Figure 2).

**Aspartyl-tRNA synthetase (AspRS)**

Three genes coding for AspRSs were found in *C. velia* in contrast to two genes in *V. brassicaformis*. *C. velia* uses duplicated AspRSs (cvel_4486; cvel_29834; cvel_13271), which likely originates in a eukaryotic nucleus, because of the position in the clade composed of all eukaryotes, with Archaea on the root. The same applies to the two sequences from *V. brassicaformis* (vbra_13004 and vbra_6695) (Supplementary Figure 1). The cyanobacterial AspRSs were identified only in green plants and green algae (Supplementary Figure 1).

The tree contains three groups of eukaryotic sequences: the first one consists of oomycetes, rhizarians, and animals (exclusively primates) and cluster with Bacteria (Chlamydiae). The second eukaryotic group includes green alga and plants, branching as a sister to cyanobacteria. The last group is related to Archaea and is composed of most of the eukaryotes (Figure 2).

**Asparaginyl-tRNA synthetase (AsnRS)**

Three genes coding for AsnRSs were found in *C. velia* while there are two genes in *V. brassicaformis*. Two genes from *C. velia* (cvel_23534; cvel_7405) and *V. brassicaformis* (vbra_5330; vbra_5336) likely originate in a eukaryotic nucleus because they branch together with other eukaryotes, however, only with those possessing plastids. Such topology may suggest their origin from the primary host nucleus (the nucleus of an alga involved in the complex endosymbiotic event). Homologs from apicomplexan parasites appear closely related to the mentioned genes (Supplementary Figure 1). The other sequence from *C. velia* (cvel_14360) also clusters with eukaryotes, particularly with stramenopiles, apicomplexans, animals in a paraphyletic assemblage with unclear origin (Supplementary Figure 1). Both *C. velia* and *V. brassicaformis* lack the cyanobacterial AsnRS (Figure 2).

The tree contains three groups of eukaryotic sequences: the sequences constituting the crown of the tree (Figure 2), the group of eukaryotes including opisthokonts, rhodophytes, amoebozoans and excavates, with Archaea on the root, which is placed in the central part of the tree. The last group of eukaryotic sequences includes chromerids, apicomplexans, and opisthokonts in a paraphyletic group sister to the Archaea related eukaryotic clade mentioned above (Figure 2).

**Glycyl-tRNA synthetase (GlyRS)**

Two genes coding for GlyRSs were found in chromerids*. C. velia* uses duplicated GlyRSs (cvel_30153; cvel_8554) likely originating in a eukaryotic nucleus because of the position in the eukaryotic clade with Archaea on the root. The same applies to one of *V. brassicaformis* sequences (vbra_22902), which has not been, however, duplicated (Supplementary Figure 1). Another sequence from *V. brassicaformis* (vbra_14962) clusters together with stramenopiles, rhizarians, and cryptophytes with Chlamydiae on the root suggesting a bacterial/organellar origin. This enzyme was identified earlier in this study as a fused (αβ)_2_-type GlyRS enzyme, which provides functions for all translationally active compartments of the cell. Both *C. velia* and *V. brassicaformis* lack the cyanobacterial GlyRS (Figure 2).

The tree contains a clade of bacterial type α_2_β_2_ sequences placed on the crown of the tree. Other sequences in the tree are of α_2_ type and constitute three groups of eukaryotic sequences: the first one is related to Archaea (Figure 2). The other eukaryotic sequences are placed throughout the bacterial α_2_ type clade and are related to Chlamydiae (e.g., *V. brassicaformis* vbra_14962), Actinobacteria (fungi) and Firmicutes (oomycetes and *Naegleria*) (Figure 2).

**Histidyl-tRNA synthetase (HisRS)**

Both *C. velia* and *V. brassicaformis* possess two genes coding for HisRS. Genes from *C. velia* HisRSs (cvel_21705) and *V. brassicaformis* (vbra_6931) originates in a eukaryotic nucleus because they are closely related recent duplicates placed in the eukaryotic clade. They form a sister group to apicomplexans *Theileria parva* and *T. gondii* (Supplementary Figure 1). The other sequences from *C. velia* (cvel_3311) and *V. brassicaformis* (vbra_20528) cluster with stramenopiles and rhizarians in the proximity of plants and green algae. Both *C. velia* and *V. brassicaformis* lack the cyanobacterial HisRS (Figure 2).

The tree contains two groups of eukaryotic sequences: the first one is related to Archaea and contains stramenopiles, cryptophytes, rhizarians, haptophytes, kinetoplastids, perkinsids, green plants, and chromerids. The second group of eukaryotic sequences is placed on the crown of the tree, with Archaea and Bacteria (*Bacteroidetes*, *Spirochaetes*, *Planctomycetes,* and *α-proteobacteria)* forming the sister group, with the eukaryotic clade placed on relatively long branch (Supplementary Figure 1).

**Lysyl-tRNA synthetase (LysRS)**

Three genes coding for LysRSs were found in *C. velia* in contrast to two genes in *V. brassicaformis*. *C. velia* uses duplicated LysRSs (cvel_7175; cvel_20258) likely originating in a eukaryotic nucleus, because of the position in the eukaryotic clade. The same applies for one sequence from *V. brassicaformis* (vbra_6201) (Supplementary Figure 1). The other *C. velia* and *V. brassicaformis* sequences cluster together with the apicomplexan *T. gondii* and the animal *Pantholops hodgsonii* closely related to alphaproteobacteria suggesting their possible mitochondrial origin (Figure 2). The cyanobacterial LysRSs were identified in stramenopiles, haptophytes, rhodophytes and rhizarians (chlorarachniophytes) (Supplementary Figure 1).

The tree contains three groups of eukaryotic sequences: the first one contains most of the eukaryotes in the tree and is rooted by Bacteria (Thermus and Aquificae) (Figure 2). The second eukaryotic group includes plants, rhodophytes, cryptophytes, stramenopiles, haptophytes, and rhizarians clustering with cyanobacterial sequences. The last group of eukaryotes forms a clade related to the alphaproteobacterial sequences (Figure 2).

**Phenylalanyl-tRNA synthetase (PheRS)**

Two genes code for α-PheRSs in *V. brassicaformis* and *C. velia*. Sequences from *C. velia* (cvel_5984) and *V. brassicaformis* sequence (vbra_3529) likely originates in a eukaryotic nucleus, because they cluster with other eukaryotes with Archaea on the root (Supplementary Figure 1). Other sequences from *C. velia* (cvel_9563) and *V. brassicaformis* (vbra_15842) constitute a well-defined and supported clade with other eukaryotes; however, its position in the tree is unclear (Supplementary Figure 1). The cyanobacterial HisRSs were identified only in stramenopiles (*Phaeodactylum tricornutum* and *Ectocarpus siliculosus*) and the rhodophyte (*Chondrus crispus*) (Figure 2).

The tree contains three groups of eukaryotic sequences: the first one is related to Archaea and contains chromerids, perkinsids, haptophytes, cryptophytes, chlorophytes, and apicomplexans. The second group of eukaryotic sequences contains that from stramenopiles and rhodophytes clustering with cyanobacterial sequences. The last group of eukaryotes, such as opisthokonts, chromerids, alveolates, stramenopiles, haptophytes, and rhizarians show unclear placement in the tree (Supplementary Figure 1).

One gene encoding for the β-PheRS was found in chromerids. Both chromerids use genes (cvel_22325; vbra_20531) originating from a eukaryotic nucleus, which constitutes clade with other eukaryotes rooted by Archaea. The cyanobacterial-like gene was found only in the oomycetes *Phytophthora sojae* (Figure 2 and Supplementary Figure 1).

**Prolyl-tRNA synthetase (ProRS)**

Two chromerid genes encoding ProRSs appear in two different positions in the tree: *V. brassicaformis* (vbra_17540) and *C. velia* (cvel_17161) sequences cluster with other eukaryotes forming Archaea related clade (Figure 2). However, eukaryotes are also placed in the sister clade rooted by Bacteria (Firmicutes, Tenericutes, Dictyoglomi), and in the two different locations in the big bacterial clade on the root of the tree. Opisthokonts and oomycetes are in the crown group in this clade, chromerids (cvel_26651; vbra_8238) are placed with other eukaryotes (animals, plants) in the middle of this bacterial clade, however, far away from cyanobacteria or alphaproteobacterial. The origins of eukaryotic sequences located inside bacterial sequences remain unclear (Supplementary Figure 1).

**Seryl-tRNA synthetase (SerRS)**

Three genes coding for SerRSs were found in *C. velia* in contrast to two genes in *V. brassicaformis*. *C. velia* use duplicated gene (cvel_28365; cvel_4888) likely originating in a eukaryotic nucleus, because of its phylogenetic position within other eukaryotes rooted by archaeal sequences. The same applies to one sequence from *V. brassicaformis* (vbra_2397) (Supplementary Figure 1). The position of the second group of chromerid sequences grouping with stramenopiles, rhizarians and apicomplexan in the proximity of alphaproteobacterial suggests their possible mitochondrial origin (Figure 2). Both *C. velia* and *V. brassicaformis* lack the cyanobacterial SerRS (Figure 2).

The tree contains three groups of eukaryotic sequences: the first one is related to Archaea and contains most of the eukaryotes (Figure 2). The second eukaryotic group includes opisthokonts (exclusively primates), rhodophytes, oomycetes, rhizarians, green algae and plants, and clusters with Bacteria (Planctomycetes, Dictyoglomi, and Spirochaetes). The last group of eukaryotic sequences includes chromerids, stramenopiles, rhizarians, haptophytes, and apicomplexans, and likely originates from alphaproteobacteria (Figure 2).

**Threonyl-tRNA synthetase (ThrRS)**

Both *C. velia* and *V. brassicaformis* encode two genes for ThrRS: one group of sequences from *V. brassicaformis* (vbra_13128) and *C. velia* (cvel_11445) cluster with apicomplexans, is placed within eukaryotes forming a clade with Gemmatimonadetes and δ-proteobacteria on the root (see Supplementary Figure 1). The second group of chromerid sequences (cvel_8549; vbra_14881) appears closely related to alphaproteobacteria (Figure 2). The cyanobacterial genes were identified only in stramenopiles, haptophytes, rhodophytes, cryptophytes, rhizarians, green algae and plants (Supplementary Figure 1).

The tree contains two groups of eukaryotic sequences: the first one is placed on the crown of the tree, contains most of the eukaryotes and is rooted by bacterial counterparts (Gemmatimonadetes, Chrysiogenetes, and δ-proteobacteria) (Figure 2). The second group of eukaryotic sequences cluster with cyanobacteria. Several eukaryotic sequences are also found to be related to alphaproteobacteria (Supplementary Figure 1).
